# FIBCD1 is an endocytic GAG receptor associated with a novel neurodevelopmental disorder

**DOI:** 10.15252/emmm.202215829

**Published:** 2022-08-02

**Authors:** Christopher W Fell, Astrid Hagelkruys, Ana Cicvaric, Marion Horrer, Lucy Liu, Joshua Shing Shun Li, Johannes Stadlmann, Anton A Polyansky, Stefan Mereiter, Miguel Angel Tejada, Tomislav Kokotović, Venkat Swaroop Achuta, Angelica Scaramuzza, Kimberly A Twyman, Michelle M Morrow, Jane Juusola, Huifang Yan, Jingmin Wang, Margit Burmeister, Biswa Choudhury, Thomas Levin Andersen, Gerald Wirnsberger, Uffe Holmskov, Norbert Perrimon, Bojan Žagrović, Francisco J Monje, Jesper Bonnet Moeller, Josef M Penninger, Vanja Nagy

**Affiliations:** ^1^ Ludwig Boltzmann Institute for Rare and Undiagnosed Diseases Vienna Austria; ^2^ CeMM, Research Center for Molecular Medicine of the Austrian Academy of Sciences Vienna Austria; ^3^ Department of Neurology Medical University of Vienna Vienna Austria; ^4^ VBC – Vienna BioCenter Campus IMBA, Institute of Molecular Biotechnology of the Austrian Academy of Sciences Vienna Austria; ^5^ Department of Neurophysiology and Neuropharmacology, Centre for Physiology and Pharmacology Medical University of Vienna Vienna Austria; ^6^ Department of Psychiatry and Behavioral Sciences, Feinberg School of Medicine Northwestern University Chicago IL USA; ^7^ Department of Genetics, Harvard Medical School Howard Hughes Medical Institute Boston MA USA; ^8^ Institute of Biochemistry University of Natural Resource and Life Sciences Vienna Austria; ^9^ Department of Structural and Computational Biology, Max Perutz Labs University of Vienna Vienna Austria; ^10^ MM Shemyakin and Yu A Ovchinnikov Institute of Bioorganic Chemistry Russian Academy of Sciences Moscow Russia; ^11^ Research Unit on Women's Health‐Institute of Health Research INCLIVA Valencia Spain; ^12^ Mercy Kids Autism Center Saint Louis MO USA; ^13^ GeneDx Gaithersburg MD USA; ^14^ Department of Pediatrics Peking University First Hospital Beijing China; ^15^ Joint International Research Center of Translational and Clinical Research Beijing China; ^16^ Michigan Neuroscience Institute University of Michigan Ann Arbor MI USA; ^17^ Departments of Computational Medicine & Bioinformatics, Psychiatry and Human Genetics University of Michigan Ann Arbor MI USA; ^18^ Department of Cellular and Molecular Medicine UCSD La Jolla CA USA; ^19^ Clinical Cell Biology, Department of Pathology Odense University Hospital Odense Denmark; ^20^ Pathology Research Unit, Department of Clinical Research and Department of Molecular Medicine University of Southern Denmark Odense Denmark; ^21^ Apeiron Biologics AG, Vienna BioCenter Campus Vienna Austria; ^22^ Cancer and Inflammation Research, Department of Molecular Medicine University of Southern Denmark Odense Denmark; ^23^ Danish Institute for Advanced Study University of Southern Denmark Odense Denmark; ^24^ Department of Medical Genetics, Life Science Institute University of British Columbia Vancouver BC Canada

**Keywords:** extracellular matrix, FIBCD1, glycosaminoglycans, genetics, neurodevelopmental disorder, Genetics, Gene Therapy & Genetic Disease, Neuroscience

## Abstract

Whole‐exome sequencing of two patients with idiopathic complex neurodevelopmental disorder (NDD) identified biallelic variants of unknown significance within *FIBCD1*, encoding an endocytic acetyl group‐binding transmembrane receptor with no known function in the central nervous system. We found that FIBCD1 preferentially binds and endocytoses glycosaminoglycan (GAG) chondroitin sulphate‐4S (CS‐4S) and regulates GAG content of the brain extracellular matrix (ECM). *In silico* molecular simulation studies and GAG binding analyses of patient variants determined that such variants are loss‐of‐function by disrupting FIBCD1‐CS‐4S association. Gene knockdown in flies resulted in morphological disruption of the neuromuscular junction and motor‐related behavioural deficits. In humans and mice, FIBCD1 is expressed in discrete brain regions, including the hippocampus. *Fibcd1* KO mice exhibited normal hippocampal neuronal morphology but impaired hippocampal‐dependent learning. Further, hippocampal synaptic remodelling in acute slices from *Fibcd1* KO mice was deficient but restored upon enzymatically modulating the ECM. Together, we identified FIBCD1 as an endocytic receptor for GAGs in the brain ECM and a novel gene associated with an NDD, revealing a critical role in nervous system structure, function and plasticity.

## Graphical abstract

Mutations in the gene *FIBCD1* were identified in two unrelated patients with undiagnosed neurodevelopmental disorders. FIBCD1 was identified as a novel receptor at the interface of hippocampal neurons and the extracellular matrix (ECM).

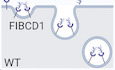

## Introduction

Neurodevelopmental disorders (NDDs) are a heterogeneous group of nervous system diseases that present with a variety of clinical symptoms, including global developmental delays, structural brain anomalies, muscular impairments, autism spectrum disorder (ASD), attention‐deficit/hyperactivity disorder (ADHD), intellectual disability (ID) and seizures (Parenti *et al*, 2020). Many NDDs have a genetic basis that affect critical developmental events such as neurogenesis, migration, axon outgrowth and guidance, synaptogenesis and synaptic function and plasticity (van Bokhoven, 2011; Vissers *et al*, 2016; Parenti *et al*, 2020; Fell & Nagy, 2021). All of these important cellular developmental milestones depend critically on instructive cues provided by the brain extracellular matrix (ECM; Smith *et al*, 2015).

Beyond development, the ECM is a dynamic microenvironment required for proper development and maintenance of CNS function in adults (Dityatev *et al*, 2010; Smith *et al*, 2015). It is structurally heterogeneous and composed primarily of glycans and glycoconjugates, including proteoglycans. Most proteoglycans in the brain are chondroitin sulphate proteoglycans (CSPGs), comprising of chondroitin sulphate (CS) glycosaminoglycan (GAG) chains conjugated to different core proteins. Spatiotemporally regulated distributions of CSPGs with variable GAG sulphate modifications correlate with specific and discrete developmental stages as part of the dramatic ECM reorganisation that accompanies and regulates brain maturation (Kitagawa *et al*, 1997; Miller & Hsieh‐Wilson, 2015; Smith *et al*, 2015). CSPGs participate in axonal outgrowth, synaptic remodelling, cellular migration and closure of the critical period of circuit development, where they condense into perineuronal nets (PNNs) that restrict synaptic plasticity and participate in memory formation, retention and extinction in adults (Gogolla *et al*, 2009; Dityatev *et al*, 2010; Sorg *et al*, 2016). The ECM is thought to play both a causal and modulatory role in many neurological disorders, including schizophrenia, Alzheimer's disease, epilepsy, autism and stroke (Soleman *et al*, 2013). Astroglial CSPG scars, which form after stroke, spinal cord injury or other injuries, prohibit axonal regeneration (Pekny & Nilsson, 2005). Therefore, understanding ECM biology is critical for rational drug design to treat many nervous system disorders and injuries.

Chondroitin sulphate proteoglycans and other ECM molecules regulate cellular behaviour by binding to specific receptors, though few CSPG receptors have been identified and associated with specific functions. Receptor protein tyrosine phosphatase sigma (PTPσ) and leucocyte common antigen‐related (LAR), as well as the Nogo receptor family members, Nogo66 receptor‐1 and 3 (NgR1 and NgR3), bind to and mediate CSPG inhibition of axonal regeneration (Shen *et al*, 2009; Dickendesher *et al*, 2012; Xu *et al*, 2015), while the adhesion protein Contactin‐1 (CNTN1) recognises CS‐4,6S (CS‐E), though the function of this interaction in the brain is poorly understood (Mikami *et al*, 2009; Mizumoto *et al*, 2012). Variants in CSPG receptors have thus far not been implicated in NDDs or psychiatric disorders.

Here, we report deleterious variants in *Fibrinogen C Domain Containing 1* (*FIBCD1*), identified by whole‐exome sequencing (WES) of two unrelated patients diagnosed with severe ASD and NDD. FIBCD1 is a type 2 receptor with high homology to ficolins and consists of a short N‐terminal cytoplasmic tail, transmembrane domain, coiled‐coil region through which FIBCD1 forms homotetramers, polycationic region and a C‐terminal extracellular fibrinogen‐related domain (FReD), which participates in ligand interactions (Schlosser *et al*, 2009). FIBCD1 acts as a pattern recognition receptor for the aminopolysaccharide chitin, abundant on fungal cell walls. Crystal structure analysis of the FReD revealed potential binding sites for additional sulphated, acetylated ligands, such as GAGs. In humans, *FIBCD1* is expressed in mucosal epithelial tissues, with highest expression in the human respiratory and gastrointestinal tracts, testes, placenta and brain (Jepsen *et al*, 2018). Despite high expression levels in the brain, the function of FIBCD1 in the CNS is unknown.

## Results

### Identification of biallelic human germline *FIBCD1* variants

**Figure 1:**
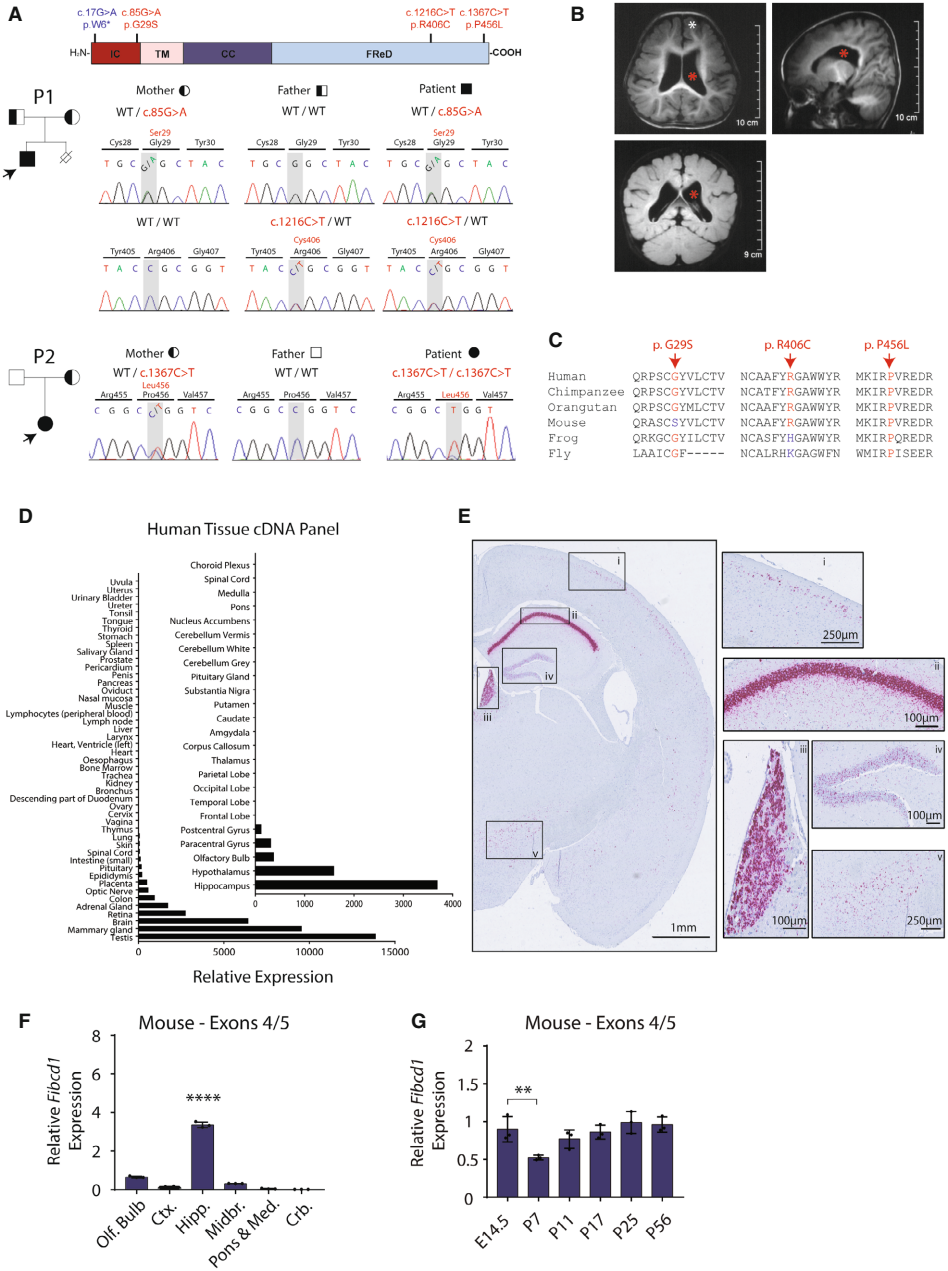
Expression of *FIBCD1* in human tissues and properties of *FIBCD1* variants identified in two cases of undiagnosed neurodevelopmental disorders ATop, schematic of FIBCD1 protein, with labelled intracellular domain (IC, red), transmembrane domain (TM, pink), coiled coil (CC, dark blue) and FReD (light blue). Location of patient variants denoted in red; blue variant denotes the control used in later experiments. Left, family pedigrees of P1 (top) and P2 (bottom) showing affected proband (filled, arrow) and carriers (half‐filled). Right, representative traces of Sanger sequencing to confirm segregation within the family. P1 variants are inherited in autosomal recessive manner; P2 exhibits inheritance by uniparental disomy.BP2 MRI images (axial, sagittal and coronal plane) showing ventriculomegaly (red asterisk), slightly thickened cortex and bilateral enlarged gyri (white asterisk).CAmino acid sequence conservation sites of patient variants Gly29Ser, Arg406Cys and Pro456Leu in different species, as labelled.D
*FIBCD1* expression in various human visceral tissues and brain regions (inset). Expression is plotted relative to the tissue with lowest detectable expression (trachea; inset, choroid plexus). *n* represents technical replicates (*n* = 2).E
*In situ* hybridisation with probe pairs specific to *Fibcd1* mRNA (purple) in mouse whole‐brain coronal section, left hemisphere shown. Insets of high *Fibcd1‐*expressing regions are (i) cortex, (ii) pyramidal cell layer of hippocampus, (iii) medial habenula, (iv) granule cell layer of the dentate gyrus and (v) hypothalamus. Scale bar sizes are as indicated, (*n* = 3).FRelative mRNA expression levels of mouse *Fibcd1* (primers binding to exons 4 and 5) normalised to *Gapdh,* in the indicated adult brain regions, analysed by RT–qPCR (*n* = 3). Olf.Bulb, olfactory bulb; Ctx., cortex; Hipp., hippocampus; Midbr., midbrain; Pons & Med, pons and medulla; Crb., cerebellum.GRelative mRNA expression levels of mouse *Fibcd1* (primers binding to exons 4 and 5) normalised to *Gapdh* in the hippocampus of the indicated developmental time points, analysed by RT–qPCR (*n* = 3). Data information: Panel (E) is representative of three independent experiments from three individual mice; for panels (F and G), each data point represents an individual mouse. Data are represented as mean, and error bars represent SD. *P*‐values were calculated by one‐way ANOVA comparing each sample to the hippocampus region (F) or the time point E14.5 (G). **P ≤ 0.01; ****P ≤ 0.0001.

Two unrelated patients presented with severe complex disorder of suspected genetic origin. Clinical synopsis of both patients reveals that they suffer primarily from nervous system dysfunctions diagnosed early in life, with distinct and shared symptoms. Patient 1 (P1) is a 12‐year‐old non‐verbal Caucasian male from a non‐consanguineous family, diagnosed with severe ASD, delayed verbal cognition, anxiety and ADHD. He has high pain tolerance, fine motor coordination deficits and mild facial dysmorphia. Additionally, he experiences frequent allergic rhinitis and sinusitis (Table 1). There is no history of neurological disease in the family; however, several members of the maternal family have learning disabilities. As part of his clinical diagnostic evaluation, WES was performed at GeneDx, USA (www.genedx.com), and the following rare variants (with minimal allele frequency of < 0.01) were prioritised: compound heterozygous *FIBCD1* Chr9:133805421 C > T; c.85 G > A; p.(G29S) and Chr9:133779621 G > A; c.1216C > T; p.(R406C) (Fig 1A), with CADD scores of 6.832 and 25.1, respectively, and a *de novo* variant in *CSMD3* Chr8: 113933925 T > C; c.1564 A > G; p.(K522E) with a CADD score of 24.7. While *CSMD3* variants have been reported in association with NDDs, most published missense variants have population data in gnomAD (Karczewski *et al*, 2020) or internal data at GeneDx, reducing the likelihood that this variant is related to the phenotype (Wu *et al*, 2018; GeneDx, Inc. personal communication). Therefore, the *FIBCD1* variants were prioritised for further analysis. Sanger sequencing determined one *FIBCD1* variant was inherited from each of the parents (Fig 1A). There were no other identified variants with confirmed association with human disease that would match the phenotype or inheritance pattern in the patient.

**Table 1:** Comparison of clinical findings and genetics of reported patients.

	P1	P2
**Background**		
Sex	M	F
Current age	12 y.o.	3 y.o.
Ethnicity	Caucasian	Chinese
FIBCD1	Compound Het.	UPD with mosaicism
	c.85G > A; c.1216C > T	c.1367C > T
	p.G29S; p.R406C	p.P456L
**Neurology**		
Diagnosis	Severe ASD	Severe NDD
Psychological evaluation	Borderline delayed cognition	Delayed social and cognitive abilities
	Anxiety	
	ADHD combined type	
Sitting and walking	Normal	Delayed
Language	Non‐verbal	Non‐verbal
Epilepsy	–	–
Intellectual disability	–	Too young to be evaluated
Sensory	High pain tolerance, sensitive touch	n/a
Motoric	Fine motor coordination deficits	n/a
**Neuroradiology**		
MRI	n/a	Slightly thickened cortex
		Decreased white matter volume
		Ventriculomegaly
		Bilateral enlarged frontal gyri
**Dysmorphias**		
Craniofacial	Triangular shaped head	Microcephaly
	Hypertelorism	Micrognathia
	Almond‐shaped eyes	Low set ears
	Posteriorly rotated and low set ears	
	Epicanthal folds	
**Other systems**		
Cardiovascular	–	Patent ductus arteriosus, resolved at 6 months
Respiratory	–	Recurrent pneumonia
Immune	Allergic rhinitis, sinusitis	–

ADHD, attention‐deficit/hyperactivity disorder; ASD, autism spectrum disorder; Het, heterozygous; NDD, neurodevelopmental disorder; UPD, uniparental disomy.

Patient 2 (P2) is a non‐verbal 3‐year‐old Chinese female from a non‐consanguineous family with no history of genetic neurological disease. She presented with delayed social and cognitive abilities and delayed sitting and walking. Magnetic resonance imaging (MRI) revealed thickened cortex, decreased white/grey matter ratio, bilateral enlarged frontal gyri and ventriculomegaly (Fig 1B). The patient also has microcephaly and dysmorphic facial features and recurrent pneumonia (Table 1). Clinical genetic testing was performed and revealed inheritance of chromosome 9 by uniparental disomy (UPD) with mosaicism. WES revealed homozygous variants of unknown significance in: *FIBCD1* Chr9:133779470 G > A; c.1367 C > T; p.(P456L) with a CADD score of 29, *UNC13B* Chr9:35376187; c.1531 T > C; p.(C511R) with a CADD score of 28.4, and *RIC1* Chr9:5765523; c.2951 C > T; p.(A984V) with a CADD score of 28.6. Variants within *UNC13B* and *RIC1* were deprioritised due to a lack of clinical similarities with published cases (Patel *et al*, 2017; Unlu *et al*, 2020; Wang *et al*, 2021), and *FIBCD1* variants (Fig 1A) were prioritised for further functional validation. All the *FIBCD1* variants named above are located in highly conserved regions (Fig 1C). Together, the clinical synopsis of the patients suggests a complex neurodevelopmental disorder with distinct and common symptoms that include delayed cognition, difficulty with language, mild facial dysmorphisms and some respiratory/immune dysfunctions (Table 1).

### *FIBCD1* is expressed in neurons of human and mouse brain

**Figure EV1:**
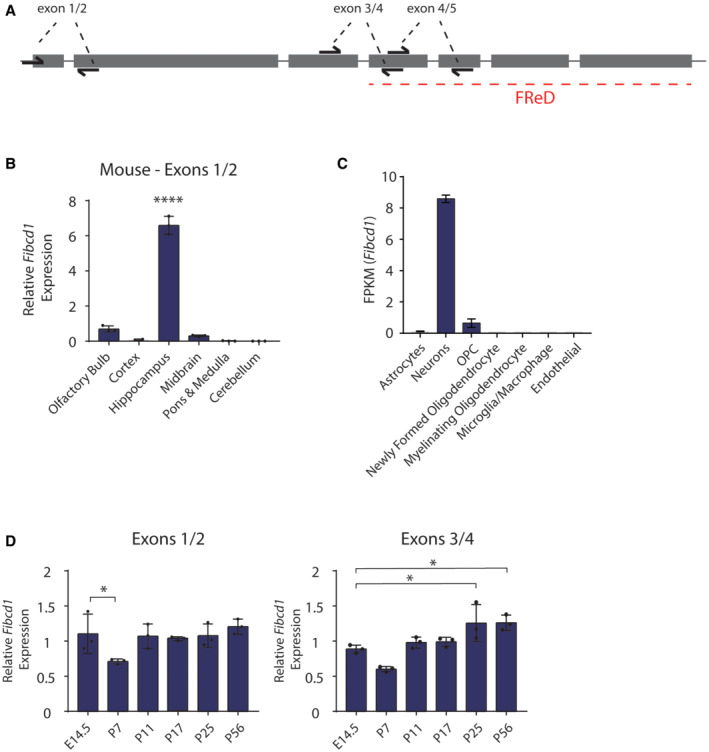
*Fibcd1* expression in the adult and developing mouse brain ASchematic of mouse *Fibcd1* exons (grey rectangles), introns (grey lines) and location of primer pair binding (“exons 1/2, 3/4 and 4/5”) used for RT–qPCR. Exon sizes are to scale; introns and primers are not. The exons coding for FIBCD1 FReD is indicated by a red dashed line.BRelative mRNA expression levels of mouse *Fibcd1* (primers binding to exon 1 and 2) normalised to *Gapdh* in the indicated brain regions, analysed by RT–qPCR (*n* = 3).C
*Fibcd1* expression in bulk populations of sorted mouse brain cell population, from brainrnaseq.org. OPC, oligodendrocyte precursor cell.DRelative mRNA expression levels of mouse *Fibcd1* (primers binding to exons 1 and 2 and exons 3 and 4) normalised to *Gapdh* in the hippocampus of the indicated time points, analysed by RT–qPCR (*n* = 3). Data information: For panels (B and D), each data point represents an individual mouse. Data are presented as mean, and error bars represent SD. *P*‐values were calculated by one‐way ANOVA comparing each sample with the hippocampus region (B) or the time point E14.5 (D). *P < 0.05; ****P < 0.0001.

Profiling human *FIBCD1* (hereafter *hFIBCD1*) expression with a cDNA array from 48 different tissues determined the brain to be the third highest *hFIBCD1‐*expressing tissue (Fig 1D) with the strongest expression in the hippocampus (Fig 1D, inset) (see Table 2 for all primer sequences). Additionally, it is expressed in the hypothalamus, olfactory bulb and areas of the cerebral cortex. In mice, *in situ* hybridisation (ISH) using complementary DNA probe pairs against mouse *Fibcd1* (hereafter *mFibcd1*) mRNA in adult coronal brain sections revealed strong hybridisation signal in the pyramidal cell layer of hippocampal CA1 and medial habenula, with a somewhat weaker signal in granule cells of the dentate gyrus, dispersed cells in superficial layers of the neocortex and the hypothalamus (Fig 1E). *mFibcd1* was expressed in the hippocampus throughout development, highest in the prenatal brain and dropping to lower levels at postnatal day 7 (P7) before returning to high embryonic levels at P25 (Figs 1F and G, and EV1B and D). In a publicly available dataset of bulk RNA sequencing of sorted mouse brain cell populations, brainrnaseq.org (Zhang *et al*, 2016), we noted *mFibcd1* expression to be highest in neurons and virtually absent from all other cell types (Fig EV1C).

**Table 2:** Materials used in this study.

**Genotyping primers:**	
*Fibcd1* WT	CGCTGGTCTTGCTGGAAG
	TCTTCTCTTCCCTCTGCACA
*Fibcd1* KO	GCAGCGCATCGCCTTCTATC
	TGGCACAGGTTAAGGAATT
**Primers for qPCR:**	
*Gapdh*	GTCGGTGTGAACGGATTTGG
	GACTCCACGACATACTCAGC
*mFibcd1(ex1‐2)*	CTGGAAGATGGTCCACGAG
	CCGTGCACAGGACATAACTG
*mFibcd1(ex3‐4)*	TCAAGGCTGACCTTCAGAGG
	GAAGCCAGCTGGGTAGTGAG
*mFibcd1(ex4‐5)*	CAGCTGGCTTCCAGGTCTAC
	CCAACCTCGGAAAAAGTTCA
*hFibcd1*	CAGGACGATGGCGTCTACTC
	GATCCTCTTGAGCCCTAGCC
**Antibodies for immunoblots:**	
b‐Actin	A5316 (Sigma)
CS‐0S (1B5)	270,431‐CS (Amsbio)
CS‐4S (2B6)	270,432‐CS (Amsbio)
CS‐6S (3B3)	270,433‐CS (Amsbio)
Anti‐V5 tag	Ab15828 (Abcam)
**Fluorescent sugars for flow cytometry:**	
Fluoresceinamine‐labelled sodium chondroitin sulphate A (A1)	AMS.CSR‐FACS‐A1 (Amsbio)
Fluoresceinamine‐labelled sodium chondroitin polysulphate (P1)	AMS.CSR‐FACS‐P1 (Amsbio)
Fluoresceinamine‐labelled sodium dermatan sulphate (B1)	AMS.CSR‐FADS‐B1 (Amsbio)
**Antibodies/dyes for immunofluorescence:**	
MAP2	Millipore 05–346
FLAG (M2)	Sigma F1804
Alexa Fluor® 546 anti‐mouse	Thermo A‐11003
Goat F(ab) anti‐mouse (IgG)	Abcam (ab6668)
Alexa Fluor® 647 AffiniPure Goat anti‐horseradish peroxidase	Jackson Immunoresearch
Mouse anti‐nc82 (Bruchpilot)	Developmental Hybridoma Studies Bank
DAPI	Carl Roth
WFA‐488	Vector Laboratories (FL‐1351)
***Drosophila* reagents**	
*Stock*	*RRID/source*
y[1] w[*]; P{w [+mC] = r4‐GAL4}3	BDSC_33832
y[1] v[1]; P{y[+t7.7] v[+t1.8] = TRiP.HMJ30271}attP40	BDSC_63703
w1118; P{GD2280}v4128/TM3	FlyBase_FBst0464025
P{KK105143}VIE‐260B	FlyBase_FBst0474536
y[1] w[*]; P{w[+m*] = nSyb‐GAL4.S}3	BDSC_51635
y[1] w[*]; P{w[+mC] = tubP‐GAL4}LL7/TM3, Sb[1] Ser[1]	BDSC_5138
PBac{UAS‐empty}VK00037	Chillian *et al.* (2020), *Star Protocols*
y[1] v[1]; P{TRiP.JF01355}attP2	BDSC_31603

### FIBCD1 deficiency leads to neurological defects in flies and mice

**Figure EV2:**
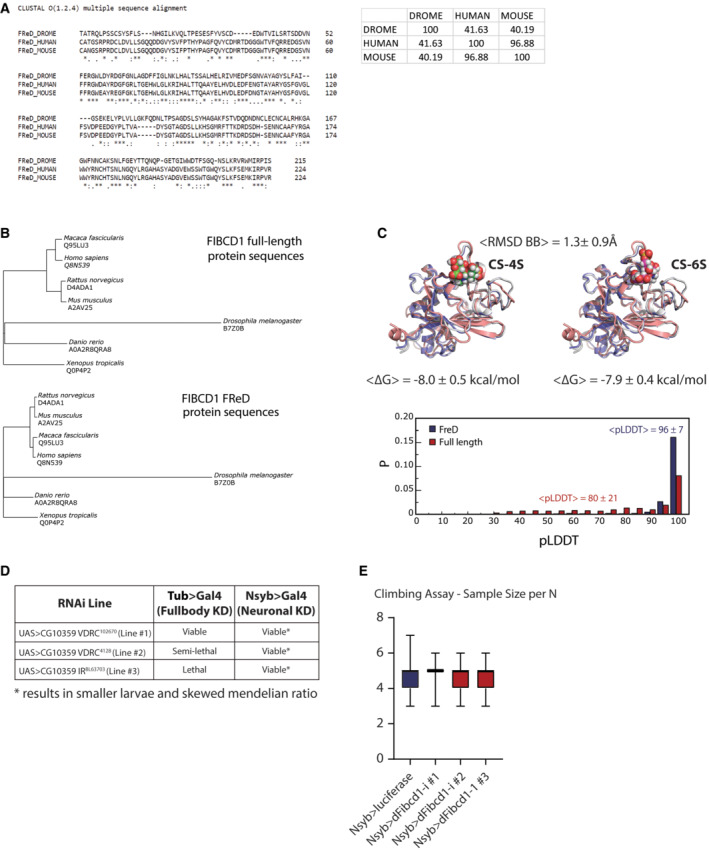
Description of *dFibcd1* AAlignment of fly (DROME), human and mouse FReD protein sequences. Inset shows percent identity matrix (% homology) between fly, human and mouse FReD protein sequences.BPhylogenetic trees based on multiple‐sequence alignments of either full‐length FIBCD1 (upper) or FReD amino acid sequences (lower) for the six species with available AlphaFold structures and *D. melanogaster* (AlphaFold structure predicted *de novo*).CUpper, 3D structures of FReD as predicted by AlphaFold for human (*H. sapiens*, blue), mouse (*M. musculus*, grey) and fly (*D. melanogaster*, pink) with CS‐4S (left) and CS‐6S (right) docked to the human variant. The average pairwise backbone RMSD and standard deviation over all possible pairs chosen from among the seven species studied are indicated above the structures. The average predicted binding free energies and standard deviations between FReD and CS‐4S (left) or CS‐6S (right) over all seven species studied are given below the structures. Lower, distributions of AlphaFold predicted local distance difference test (pLDDT) scores for predictions of either full‐length FIBCD1 or FReD alone pooled over all seven species studied. The high confidence of the FReD structure predictions is reflected in the extreme value of the average pLDDT score (96 ± 7).DSummary of 3 *D. melanogaster* RNAi lines crossed to full body GAL4 driver (tubulin) or neuron‐specific (Nsyb) and the effects on viability.ENumber of flies analysed for the climbing assay in Fig 2D. Box plots depict data mean and upper and lower quartile; whiskers are the minimum and maximum number

To investigate the physiological role of FIBCD1 *in vivo*, we studied the phenotypic outcomes of FIBCD1 deficiency in two organismal models: *Drosophila melanogaster* and *Mus musculus*. First, we identified the *D. melanogaster* gene *CG10359* as a potential orthologue of *FIBCD1*. While *CG10359* has no assigned function, it is annotated in Flybase (FBgn0035452) with GO terms such as “chitin binding” and “extracellular region” of cellular component. Based on the protein sequences, InterPro predicts a C‐terminal fibrinogen‐like domain similar to FIBCD1, with a high degree of amino acid sequence homology with human and mouse FIBCD1 (Fig EV2A). Furthermore, the structures of the FReDs in several different species, including *H. sapiens*, *M. fascicularis*, *R. norvegicus*, *M. musculus*, *D. rerio*, *X. tropicalis* and *D. melanogaster* (Fig EV2B), as predicted by AlphaFold Protein Structure Database (Jumper *et al*, 2021; Varadi *et al*, 2022), are found to be extremely similar to each other according to backbone RMSD values (1.3 ± 0.9 Å on average, Fig EV2C), supporting the possibility of their evolutionarily conserved function.

**Figure 2:**
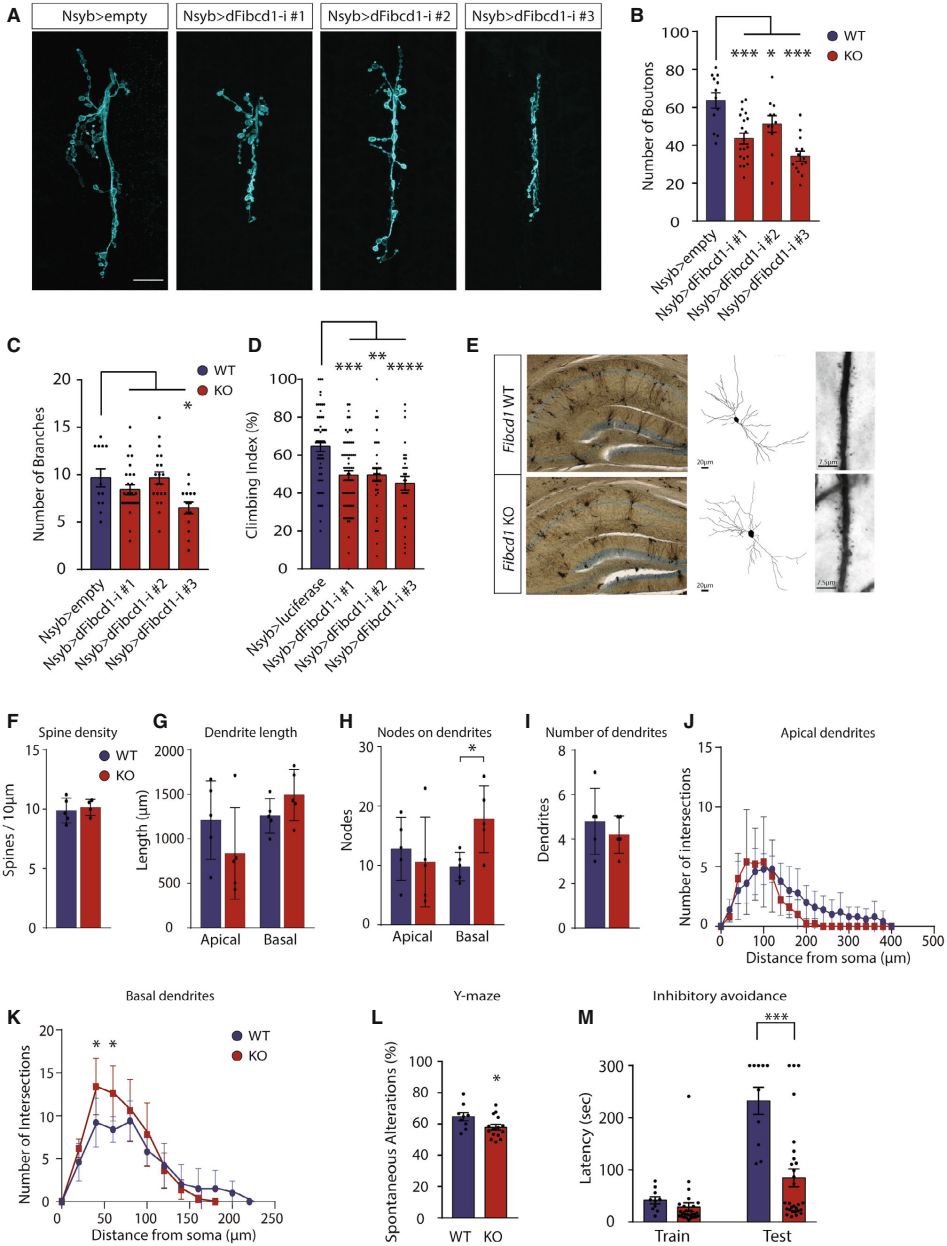
Neurological deficits in FIBCD1‐deficient mice and flies AImmunofluorescent images of control and neuronal (Nsyb) *CG10359* (*dFibcd1*) RNAi‐mediated knockdown *D. melanogaster*, 3^rd^ instar larvae NMJ (NMJ6/7) stained with anti‐horseradish peroxidase antibodies. Empty control and RNAi‐mediated knockdown of *CG10359* (*dFibcd1‐i*) lines #1, 2 and 3 shown. Scale bar = 20 μm. Representative images of three independent experiments.B, CQuantification of (A), control and *CG10359* knockdown lines NMJ neuron bouton number (B) and NMJ neuron axon branch points (C). *n*(empty) = 12; *n*(line #1) = 20; *n*(line #2) = 11; *n*(line #3) = 14.DNegative geotaxis assay of adult *Drosophila* control and RNAi lines #1, #2 and #3 compared to control lines expressing RNAi targeting luciferase. Climbing index represents the percentage of flies that crossed the 5 cm vial mark within 5 s after gentle tapping to the bottom of the vial. *N* is the number of tested vials: *n*(luciferase) = 53; *n*(line #1) = 63; *n*(line #2) = 36; *n*(line #3) = 31.For flies per vial, see Fig EV2D.ERepresentative coronal section images of Golgi–Cox staining of *Fibcd1* WT and KO hippocampi (left), Neurolucida tracing of hippocampal CA1 pyramidal neurons (middle) and apical dendrites with spines (right). Scale bars as indicated.F–IQuantifications of (F) dendritic spine density, (G) total length of apical and basal dendrites, (H) dendritic nodes in apical and basal dendrites, and (I) number of dendrites (*n* = 5).J, KSholl analysis of apical and basal dendrites (*n* = 5).LPercentage of mouse spontaneous alterations in the Y‐maze (*n*(*Fibcd1* WT) = 9; *n*(*Fibcd1* KO) = 15).MLatency to enter the dark (foot shock) chamber during the inhibitory avoidance task at training and testing (24 h post‐training) days (*n*(*Fibcd1* WT) = 8; *n*(*Fibcd1* KO) = 15). Data information: For panels (B–D), each data point represents an individual NMJ; for panels (F–I, L and M), each data point represents an individual mouse. Data are represented as mean, and error bars represent SEM. *P*‐values were calculated using two‐way ANOVA (panels B and C), one‐way ANOVA (panel D) or unpaired Student's *t*‐test (panels F–I, L and M). For panels (J and K), *P*‐values were calculated using two‐way ANOVA and differences at individual distances in the Sholl analysis were corrected for multiple comparisons by Bonferroni's multiple comparisons test. *P ≤ 0.05; **P ≤ 0.01; ***P ≤ 0.001; ****P ≤ 0.0001.

To assess the function of *CG10359* (hereafter *dFibcd1*), we knocked down *dFibcd1* by crossing three independent RNAi constructs targeting *dFibcd1* (downstream of *UAS* promoter sequence, hereafter as lines #1, #2 and #3) with lines expressing *GAL4* under the control of either the tubulin (tub) promoter for whole‐body RNAi expression or the *neuronal Synaptobrevin* promoter (*Nsyb*) for neuronal expression of RNAi. As full body knockdown of *dFibcd1* was lethal or semi‐lethal in 2 of 3 lines (Fig EV2D), we proceeded only with neuronal knockdown of *dFibcd1*, which affected neuronal development visualised by abnormal morphology at the larva neuromuscular junction (NMJ; Fig 2A). All three neuronal knockdown lines exhibited reduced number of pre‐synaptic boutons (Fig 2B), and line #3 further exhibited reduced degree of neuronal branching (Fig 2C). To assess whether these developmental defects led to neurological phenotypes in adults, we assessed fly climbing behaviour by negative geotaxis assay. We found that neuronal knockdown of *dFibcd1* resulted in reduced climbing ability when compared to controls in line with delayed walking abilities noted for P2 (Figs 2D and EV2E).

**Figure EV3:**
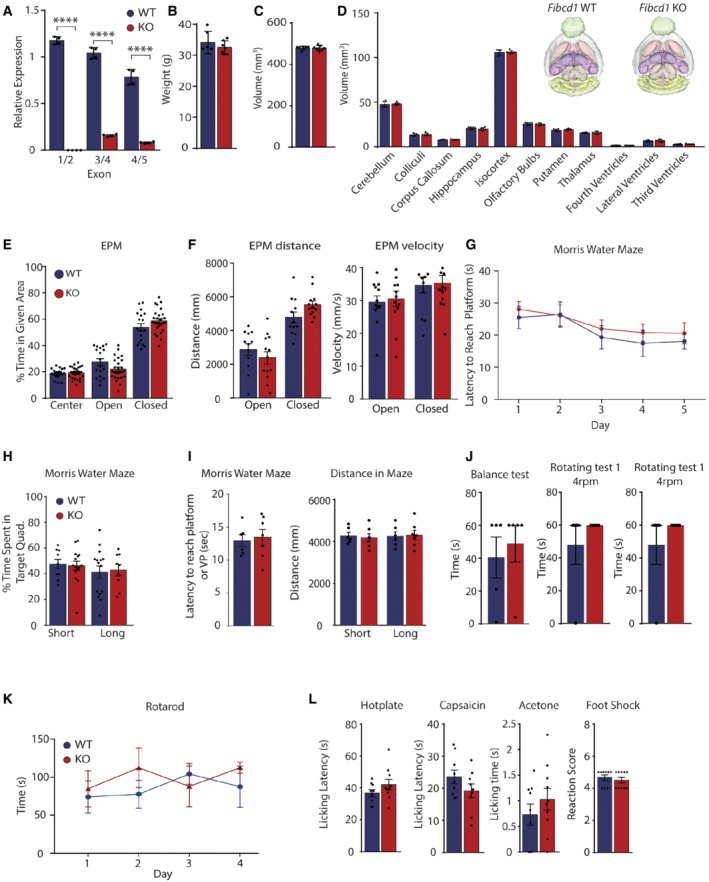
Phenotyping FIBCD1‐deficient mice ART–qPCR of *Fibcd1* WT and KO adult mouse hippocampi (*n* = 4) using primer pairs binding to indicated exons (see Fig EV1A).B–DBody weight (B) total brain volume (C) and brain volumes of denoted regions (D) of the indicated genotypes as assessed by MRI volumetric analysis. *n*(*Fibcd1* WT) = 6; *n*(*Fibcd1* KO) = 7. Insets are 3D representative MRI renditions of control (left) and *Fibcd1* KO (right) adult male brains with analysed brain regions pseudo‐coloured.EPercentage of time mice spent in the centre, open and closed arms of the elevated plus maze (EPM). *n*(*Fibcd1* WT) = 18; *n*(*Fibcd1* KO) = 26.FThe distance (left) and velocity (right) travelled by the mice in the open and closed arm of the EPM. *n*(*Fibcd1* WT) = 18; *n*(*Fibcd1* KO) = 26.GAverage latency of eight trials for each cohort to reach the target platform during the five training days in the Morris water maze (MWM). *n*(*Fibcd1* WT) = 9; *n*(*Fibcd1*KO) = 15.HPercentage of time spent in the target quadrant during the short‐ and long‐term probe trial in the MWM. *n*(*Fibcd1* WT) = 9; *n*(*Fibcd1* KO) = 15.ILeft, latency to reach the visible platform (VP) in the MWM; and right, distance travelled during the short‐ and long‐term probe trials in the MWM. *n*(*Fibcd1* WT) = 6; *n*(*Fibcd1* KO) = 7).JLeft, latency of the animal to fall off the beam in the non‐rotating Rotarod performance test; right, latency to fall of the 4 rpm rotating beam of two independent trials (*n* = 5).KAverage latency of four consecutive trials to fall off the 4–40 rpm rotating beam in a Rotarod performance test (*n* = 5).LAcute pain responses to hotplate, intraplantar capsaicin injections or acetone drop quantified as time to first response or time spent licking or biting the injected paw, respectively, and reaction score to electrical foot shock. *n*(*Fibcd1* WT) = 9; *n*(*Fibcd1* KO) = 10. Data information: For panels (A–L), each data point represents an individual mouse. Data are shown as mean values, and error bars represent SEM. *P* values were calculated using unpaired Student's *t*‐test. ****P < 0.0001.

To investigate the function of FIBCD1 in mammals, we obtained *Fibcd1* KO mice (MGI:5007144; Tang *et al*, 2010) and validated a lack of *mFibcd1* expression in KO hippocampi by qPCR (Fig EV3A) (see Table 2 for primer sequences). The KO mice were healthy and viable and exhibited no obvious abnormalities: normal body weight (Fig EV3B), normal overall brain volume (Fig EV3C) and no volumetric differences between any of the brain regions examined as assessed by 15.2T MRI (Fig EV3D).

To ascertain whether the gross morphological aberrations noted at the *D. melanogaster* NMJ are also evident in the FIBCD1‐deficient mouse brain, we performed Golgi–Cox staining of 100‐μm coronal sections of *Fibcd1* WT and KO brains and focused our analysis in the FIBCD1‐rich hippocampal CA1 pyramidal cells (Fig 2E). We did not detect differences between *Fibcd1* WT and KO littermates in the density of spines on the proximal apical dendrites (Fig 2F). Except for an increase in the number of branches (nodes) of basal dendrites in KOs as compared to controls, we did not detect any significant differences in the length of basal dendrites or the total length and number of branches of apical dendrites (Fig 2G–I). Sholl analysis used to determine morphological differences between neurons confirmed the slightly increased basal dendritic complexity 40–60 μm away from the soma, and no significant difference in apical dendritic arborisation between *Fibcd1* WT and KO neurons (Fig 2J and K).

To assess a role for *mFibcd1* in hippocampal function, *Fibcd1* WT and KO adult mice were subjected to several behavioural hippocampal‐dependent learning tasks. Firstly, we noted there were no differences in baseline anxiety levels as measured by the elevated plus maze (EPM) between the two cohorts of mice, in contrast to the increased levels of anxiety noted for P1 (Fig EV3E). There were also no differences in the distance and velocity of exploratory behaviour of the mice during the EPM assessment (Fig EV3F), in contrast to the *dFibcd1* fly model and delayed walking skills noted for P2. However, we found that while *Fibcd1* KO mice were able to perform above chance in spontaneous alternation of the Y‐maze, they were significantly impaired in spatial working memory as compared to their control littermates (Fig 2L). Further, KO animals were significantly impaired in fear‐associated learning in the inhibitory avoidance (IA) task as compared to WTs (Fig 2M). Performances in the Morris water maze (MWM), on the contrary, revealed no difference in the acquisition of spatial learning nor short‐ or long‐term memory retention between *Fibcd1* WT and KO mice (Fig EV3G and H). We did not note any deficiencies in the speed or distance swam during the MWM testing between the cohorts (Fig EV3I). To directly assess whether there are any balance, grip strength, coordinated movement or locomotion deficiencies in the mice as was observed in flies and P2, we assessed motor abilities on a Rotarod performance test. Mice were first placed on the beam without rotation, where we noted no difference in the latency to fall off between *Fibcd1* WT and KO littermates (Fig EV3J and K). Two trials with constant 4 rpm rotation also showed no difference in latency to fall off the beam. Finally, there was also no difference in four consecutive trials on an accelerating 4–40 rpm beam in the latency to fall between the two cohorts confirming KO mice do not exhibit any locomotion or coordinated movement deficiencies. As P1 exhibited higher pain tolerance and sensitivity to touch, we further tested somatosensory perception in the *Fibcd1* KO mice. Nociceptive responses to noxious chemicals, heat stimulation or mild foot shock in sensory nervous system processing of acute pain were indistinguishable between WT and KO littermates (Fig EV3L). To summarise, abrogation of *dFibcd1* in flies suggests a role in neurodevelopment and locomotion, and *mFibcd1* appears to be critical to specific hippocampal‐dependent learning but not in coordinated movement or sensory function in mice.

### FIBCD1 deficiency impacts synaptic remodelling that is rescuable by CSPG digestion

To validate our behavioural findings and ascertain FIBCD1's role at the synapse, we next performed field recordings in acute hippocampal slices from adult *Fibcd1* WT and KO mice. As the GAG‐rich ECM strongly influences synaptic plasticity, and potential binding sites for GAGs were identified in the extracellular domain of FIBCD1 (Shrive *et al*, 2014), we performed the following electrophysiological recordings in the presence or absence of chondroitinase ABC (ChABC), a bacterial enzyme used to degrade CSPGs or penicillinase (Pen) as a negative control.

**Figure 3:**
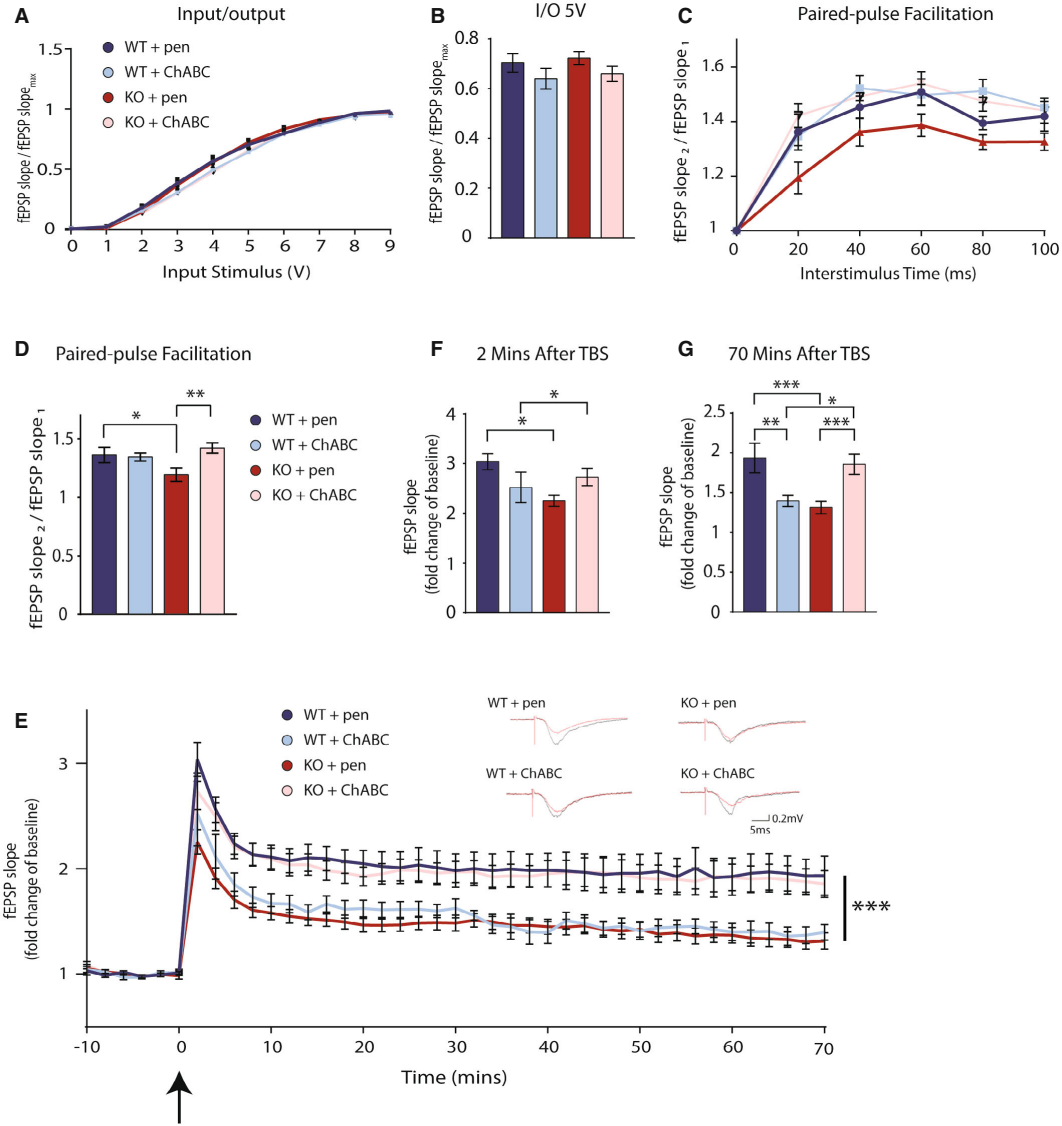
Impaired synaptic remodelling in FIBCD1‐deficient mice is rescued by ChABC treatment A, BInput/output assessment of synaptic transmission in CA3‐CA1 Schaffer collateral pathway of adult mouse hippocampal slices. *Fibcd1* WT (blue) and KO (red) hippocampal slices, pre‐treated with penicillinase (pen) or chondroitinase ABC (ChABC). *n*(WT + pen) = 22; *n*(KO + pen) = 27; *n*(WT + ChABC) = 21; *n*(KO + ChABC) = 30.C, DPaired‐pulse facilitation in CA3‐CA1 Schaffer collateral pathway of acute hippocampal slices from *Fibcd1* WT and KO mice. Pre‐treatment with pen or ChABC as labelled. *n*(WT + pen) = 17; *n*(KO + pen) = 20; *n*(WT + ChABC) = 19; *n*(KO + ChABC) = 25.ELong‐term potentiation in CA3‐CA1 Schaffer collateral pathways of acute hippocampal slices. Theta‐burst stimulation (TBS) is at time 0 indicated by the arrow. *n*(WT + pen) = 9; *n*(KO + pen) = 15; *n*(WT + ChABC) = 6; *n*(KO + ChABC) = 12. Insets are representative traces.F, GLTP fold change of baseline at 2 (F) and 70 (G) minutes post‐theta‐burst stimulation (TBS) in adult mouse hippocampal slices. *n*(WT + pen) = 9; *n*(KO + pen) = 15; *n*(WT + ChABC) = 6; *n*(KO + ChABC) = 12. Data information: Each *n* represents an individual slice preparation from seven different animals per condition. Data are plotted as mean, and error bars represent SEM. *P* values were calculated by one‐way ANOVA. *P ≤ 0.05; **P ≤ 0.01; ***P ≤ 0.001.

We first examined the baseline synaptic properties of the CA3 Schaffer collateral to CA1 circuit, a key pathway implicated in the formation and maintenance of spatial memories (Wilson & Tonegawa, 1997). We measured input/output relationships but found no significant differences among all conditions (Fig 3A and B), indicating that the ChABC treatment does not alter the properties of basal synaptic transmission in agreement with the previous literature (Bukalo *et al*, 2001). We next examined paired‐pulse‐induced facilitation, a form of short‐term pre‐synaptic plasticity directly related to the probability of neurotransmitter release (Nicoll & Malenka, 1999). We observed no differences between Pen‐ and ChABC‐treated WT slices, in agreement with the previous literature (Bukalo *et al*, 2001). However, slices obtained from KO mice treated with Pen showed reduced paired‐pulse facilitation compared with Pen‐treated WT slices (Fig 3C and D). Remarkably, this reduction was restored to WT levels in the ChABC‐treated KO slices (Fig 3C and D). Finally, we examined the effects of theta‐burst stimulation (TBS)‐induced long‐term potentiation (LTP) of CA1 synaptic strength such as the kind recorded during learning events in mice. Consistent with the previous literature (Bukalo *et al*, 2001; Kochlamazashvili *et al*, 2010), ChABC treatment reduced, but did not abolish, potentiation in WT slices, starting at the first recorded pulse (Fig 3E–G, light blue vs. dark blue traces). In slices from KO mice pre‐treated with Pen, we noted reduced potentiation compared with Pen‐treated WT slices (i.e. baseline differences; Fig 3E–G, dark blue vs. dark red traces), similar to ChABC‐treated WT slices (light blue trace), but, remarkably, this deficit in LTP was similarly rescued by pre‐treating KO slices with ChABC (Fig 3E–G, pink trace). Together, these data confirm that FIBCD1 is essential for normal hippocampal synaptic function in adult mice and suggest that such deficits in pre‐ and postsynaptic forms of plasticity in the KO hippocampus underlie the learning deficits described above (Fig 2L and M), via dysregulation of ECM signalling.

### FIBCD1 binds to glycosaminoglycans

To characterise the molecular function of FIBCD1, we first identified its endogenous ligand. Previous work has shown FIBCD1 to bind and facilitate the endocytosis of acetylated structures including N‐acetyl‐glucosamine, a component of chitin (Schlosser *et al*, 2009). As stated above, the only indication thus far of a potential endogenous ligand has come from the determination of the crystal structure of the extracellular FReD, which revealed potential binding sites for sulphated, acetylated ligands such as GAGs.

**Figure 4:**
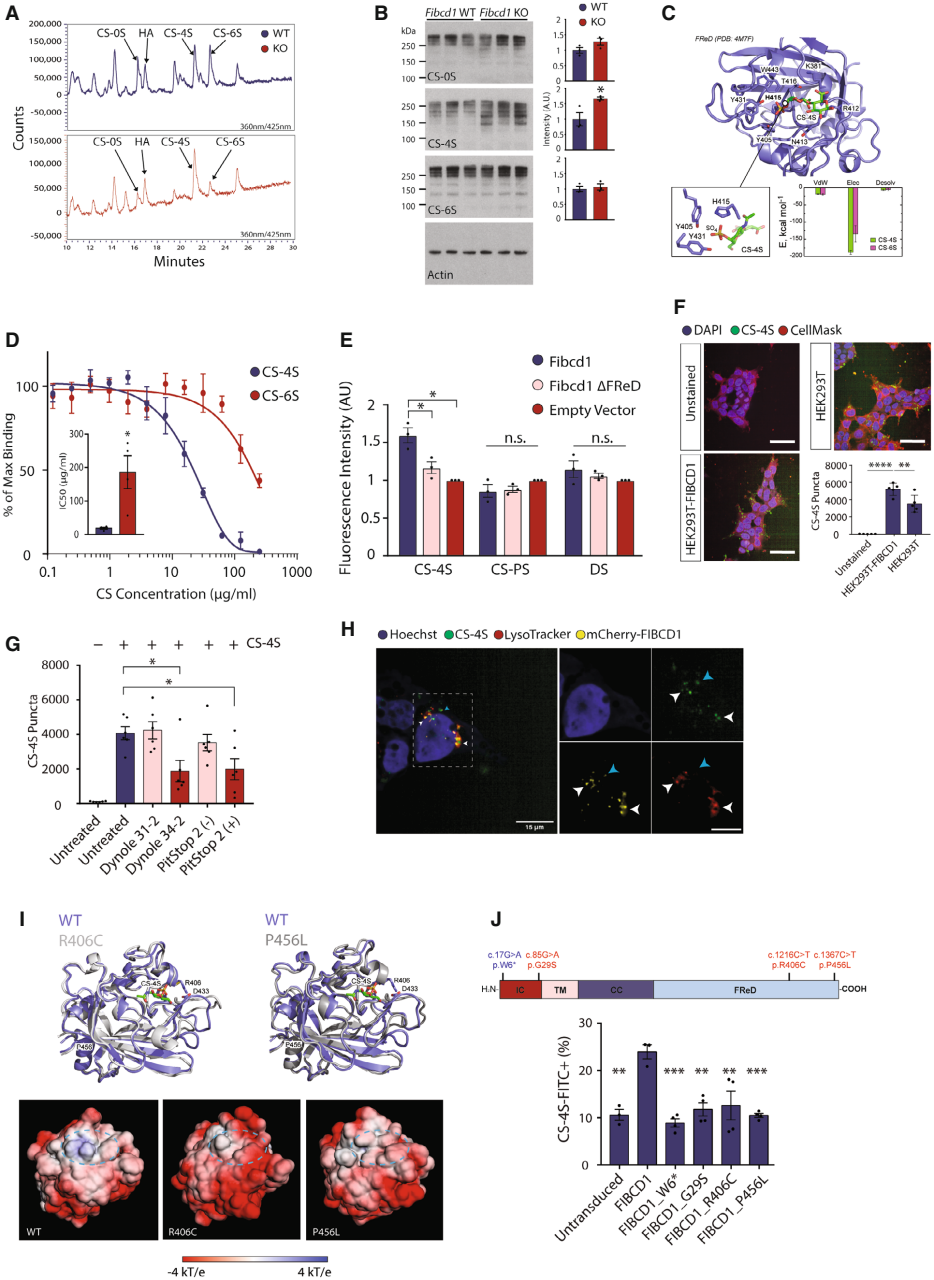
FIBCD1 is an endocytic receptor for hippocampal glycosaminoglycans AHPLC traces representative of three independent experiments of variously sulphated GAGs (as labelled) in adult *Fibcd1* WT (top, blue) and KO (bottom, red) CA1 pyramidal cell layer hippocampi. Unsulphated CS, CS‐0S; hyaluronic acid, HA; 4‐O‐sulphated CS, CS‐4S; 6‐O‐sulphated CS, CS‐6S.BImmunoblot analysis (left) and quantification of signal intensity (right) of *Fibcd1* WT (blue) vs. *Fibcd1* KO littermates (red) adult hippocampi with antibodies against CS‐0S, CS‐4S, CS‐6S and actin as a loading control. Each lane represents an independent animal (*n* = 3). Protein marker sizes are indicated.CTop binding pose for *in silico* docking of CS‐4S to FIBCD1 FReD (PDB 4M7F). Inset (left) is the orientation of CS‐4S within the FReD binding pocket and (right) binding free energy of CS‐4S vs. CS‐6S. Van der Waals (vdW), electrostatic (Elec) and desolvation (Desolv) components of binding free energy change.DCompetitive ELISA with increasing concentrations of CS‐4S (blue circles) or ‐6S (red circles) incubated with recombinant FIBCD1 FReD and acetylated BSA. Inset is IC50 concentrations for CS‐4S and CS‐6S (*n* = 4).EFlow cytometric analysis of N2a cells expressing full‐length mFIBCD1, mFIBCD1ΔFReD or empty vector control incubated with FITC‐tagged chondroitin‐4‐sulphate (CS‐4S), polysulphated chondroitin sulphate (CS‐PS) or dermatan sulphate (DS) (*n* = 3).FConfocal images depicting internalisation of FITC‐tagged CS‐4S by FIBCD1‐overexpressing HEK293T lines compared with untransduced cells and unstained cells. Left, representative images; right, quantification. Data are plotted as total puncta per condition (*n* = 5). Cells are further stained with CellMask Orange (cellular membrane) and Hoechst (nuclei). Scale bar = 50 μm.GInternalisation of FITC‐tagged CS‐4S by HEK293T‐FIBCD1 cells treated with inhibitors of endocytosis, Dynole 34–2 and PitStop 2 (+) vs. their respective negative control compounds with no inhibitory properties, Dynole 31–2 and PitStop 2 (−) (*n* = 6).HRepresentative (of two independent experiments) images of HEK293T cells overexpressing mCherry‐FIBCD1 fusion protein (yellow) stained with Hoechst (nuclei, blue), lysosomal vesicles (LysoTracker, red) and FITC‐CS‐4S (green). White arrows indicate co‐localisation of CS‐4S, lysosomal vesicles and FIBCD1; blue arrow indicates co‐localisation of FIBCD1 and CS‐4S but not lysosomal vesicles. Scale bar = 15 μm. Inset, digital zoom of HEK293T images showing co‐localisation. Scale bar = 7.5 μm.ITop, superposition ribbon diagrams of the WT FReD domain (dark blue) with R406C (left) and P456L (right) mutants (in grey). The loops surrounding the ligand binding site (389–399 and 423–448) exhibit the largest structural rearrangement in both mutants. Bottom, comparison of the electrostatic potential mapped onto the solvent‐accessible surface between WT and the two variant FReDs.JTop, schematic depiction of FIBCD1 protein and location of patient variants (red) and W6* control (blue). Bottom, flow cytometric analysis of untransduced HEK293T cells (*n* = 3), or expressing constructs with full‐length wild‐type human FIBCD1 (*n* = 3), FIBCD1 with the W6* early stop variant as control (FIBCD1_W6*; *n* = 4), or the three patient variants (as labelled, *n* = 4) incubated with FITC‐tagged CS‐4S represented as percentage of CS‐4S‐FITC relative to unstained control. Data information: For panel (B), each data point represents hippocampal protein isolates from an individual mouse; for panel (D) inset, each data point represents a technical replicate; for panels (E, F, G and J), each data point represents an individual cell preparation. Data are shown as mean values ± SEM. *P* values were calculated using one‐way ANOVA (panels E, F, G, J) or paired Student's *t*‐test (panels B, D). *P ≤ 0.05; **P ≤ 0.01; ***P ≤ 0.001; ****P ≤ 0.0001.

To investigate whether FIBCD1 interacts with components of the brain ECM *in vivo*, we analysed the composition of the ECM in the absence of FIBCD1. We surveyed the hippocampal glycome by high‐performance liquid chromatography (HPLC) of *Fibcd1* WT and KO mice and detected alterations in various GAG moieties in the KO hippocampi, most notably a relative increase in CS‐4S and a decrease in CS‐6S compared with controls (Fig 4A). We next immunoblotted for various CS species in hippocampal protein lysates pre‐digested with ChABC, which reveals CS “stub” epitopes detectable by antibodies. We observed a significant increase in CS‐4S stub abundance in lysates from KO animals, whereas the ‐0S and ‐6S stubs were unchanged (Fig 4B).

**Figure EV4:**
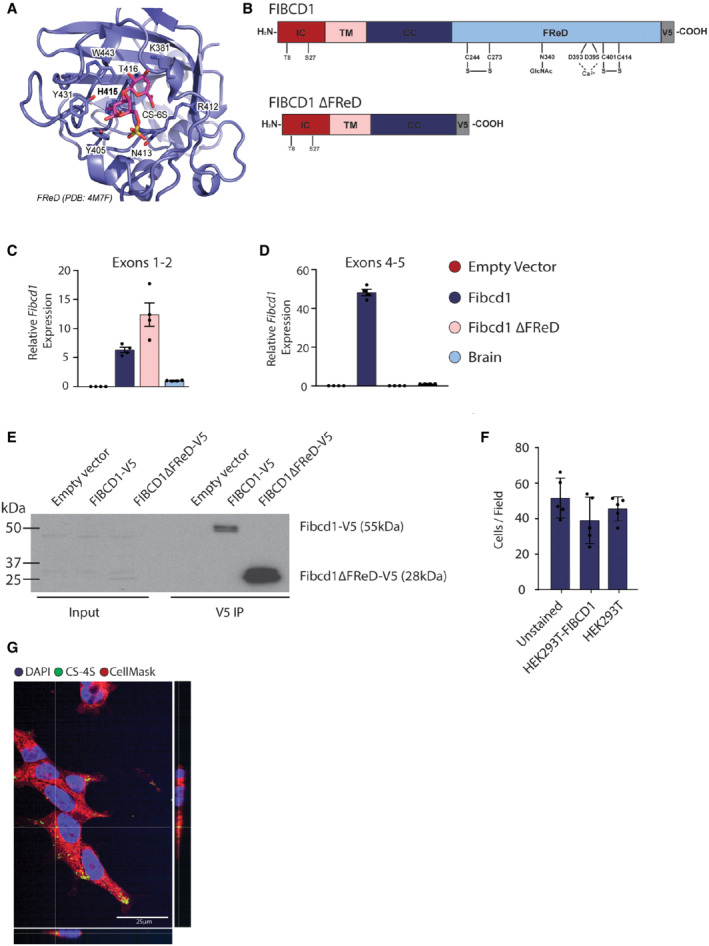
Docking site of CS‐6S in FIBCD1 FReD and validation of mFIBCD1 overexpressing N2a cell lines ATop binding pose for *in silico* docking of CS‐6S to FIBCD1 FReD (PDB 4M7F).BSchematic representation of FIBCD1 domains, IC, intracellular domain (red); TM, transmembrane domain (pink); CC, coiled‐coil domain (dark blue); FReD (light blue); and location of V5‐tag (grey) in full‐length mFIBCD1 cDNA and truncated mFIBCD1 lacking the FReD (FIBCD1 ΔFReD).C, DRelative mRNA expression levels of *Fibcd1* in the N2a cells overexpressing full‐length (*Fibcd1*) or truncated *FIBCD1* (*Fibcd1 ΔFReD*) and adult mouse WT brain for comparison, analysed by RT–qPCR (*n* = 4) using primers binding to exons 1 and 2 before the FReD domain (C) or to exons 4 and 5 spanning the sequence encoding part of the FReD (D). Note the complete absence of endogenous *Fibcd1* expression in the “empty vector” (red bar) control and the complete absence of expression when using primers complementary to exon 4/5 (D), which span the FReD (see Fig EV1A) in the *Fibcd1* ΔFReD construct (C, pink bar), validating the generated cell lines. *Gapdh* was used as housekeeping control, and values obtained from a control brain sample were set to 1.EValidation of transgenic N2a cell line at the protein level by immunoprecipitation with anti‐V5 antibody as bait. Input (left) and V5‐immunoprecipitated (right) lysates from N2a cells expressing V5‐tagged full‐length mFIBCD1 (mFIBCD1‐V5, predicted size of 55 kDa), V5‐tagged mFIBCD1 lacking the FReD (V5‐FIBCD1 ΔFReD, predicted size of 28 kDa) or the empty vector as negative control. Protein marker sizes are indicated.FNumber of HEK293T cells per field during the CS‐4S internalisation experiments, linked to Fig 4F (*n* = 5).GRepresentative immunofluorescent images of HEK293T‐FIBCD1 cells stained with FITC‐CS‐4S (green), CellMask (red) and Hoechst (blue); bottom and right panels are orthogonal views. Scale bar = 25 μm. Data information: For panels (C, D and F), each data point represents an individual cell preparation and data are plotted as mean with error bars representing SD.

To further investigate the relationship of FIBCD1 to CS‐4S and CS‐6S, top binding poses for GAGs including CS‐4S and CS‐6S were identified using *in silico* molecular docking and an X‐ray structure of the human extracellular FReD (PDB 4M7F), followed by post‐rescoring of docking solutions as described previously (Ribeiro Ede Jr *et al*, 2014). According to the scoring function, CS‐4S exhibits a better fit to the FReD as compared to CS‐6S (45.3 vs. 43.3), with the orientations of the two ligands on the FReD surface being nearly orthogonal to each other (Figs 4C and EV4A). Importantly, the orientation of CS‐4S, with its sulphate group packing tightly into a pocket formed by Y405, H415, and Y431 residues of the FReD, leads to a more favourable electrostatic interaction and subsequently lower binding free energy (ΔΔG value of −1.3 kJ/mol) as predicted by a linear model, published elsewhere (Kurkcuoglu *et al*, 2018). Notably, all species AlphaFold structures are predicted to strongly bind both CS‐4S and CS‐6S, with highly similar binding free energies (−8.0 ± 0.5 kcal/mol with CS‐4S on average, and −7.9 ± 0.4 kcal/mol with CS‐6S on average) further indicating functional conservation (Fig EV2C).

To characterise binding affinities of FIBCD1 to CS‐4S and CS‐6S, we performed competitive ELISA experiments as described previously (Schlosser *et al*, 2009). Using a previously reported FIBCD1 ligand, acetylated BSA, and increasing concentrations of CS‐4S or CS‐6S, we determined a preference of FIBCD1 to bind CS‐4S over CS‐6S, with an approximately 10‐fold lower IC_50_ of CS‐4S compared with CS‐6S (Fig 4D).

To assess FIBCD1 binding to GAGs in a cellular context, we cloned V5‐tagged full‐length *mFibcd1* cDNA and a truncated version without the FReD (*Fibcd1* ^*ΔFReD*^; Fig EV4B). We overexpressed the two *mFibcd1* constructs in the mouse N2a cell line and by RT–qPCR and immunoblot analyses confirmed the overexpression of FIBCD1 and V5‐reactive bands at predicted molecular weights (Fig EV4C–E). We then incubated the cells with fluoresceinamine (FITC)‐tagged CS‐4S, polysulphated CS (CS‐PS) and dermatan sulphate (DS) and acquired the cells by flow cytometry. We determined that cells expressing full‐length WT *mFibcd1* showed increased V5^+^/FITC^+^ fluorescence intensity compared with cells expressing empty vector or *Fibcd1* ^*ΔFReD*^, while this was not the case for cells incubated with CS‐PS or DS (Fig 4E). To investigate whether FIBCD1 facilitates internalisation of GAGs, we incubated HEK293T cells stably overexpressing *hFibcd1*with FITC‐tagged CS‐4S and observed an increased uptake of CS‐4S in FIBCD1‐expressing cells compared with untransduced controls (Figs 4F and EV4F–G), which was abrogated by compounds that inhibit endocytosis, Dynole and PitStop (Fig 4G). Internalised CS‐4S co‐localised with both FIBCD1 and LysoTracker, which stains lysosomal vesicles (Fig 4H), indicating that FIBCD1 facilitates endocytosis of CS‐4S to the lysosomes. In summary, we conclude that FIBCD1 is an endocytic receptor for GAGs of the brain ECM, with a preference for CS‐4S, that regulates the composition of the brain ECM.

### Identified patient *FIBCD1* variants are loss‐of‐function variants

**Figure EV5:**
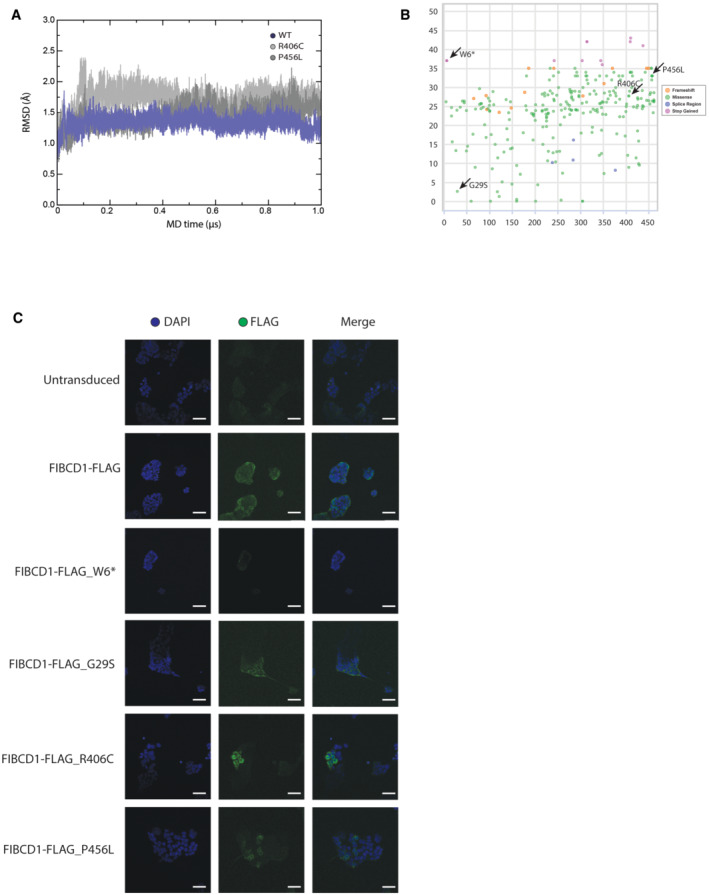
Additional human FIBCD1 data ATime course of the backbone root‐mean‐square deviation (RMSD) from the starting configuration for WT (blue), R406C (pink) and P456L (red) MD simulations.BMissense, frameshift, splice region and stop gain variants extrapolated from the gnomAD database present in the population, colour code is indicated in the figure. Each dot represents one distinct variant, amino acid position and CADD score indicated on *x*‐ and *y*‐axis. Denoted with arrows are the variants discussed in the present study.CValidation of FLAG‐FIBCD1 expression in stably expressing HEK293T cells by immunofluorescence. Note the absence of signal in untransduced cells and cells expressing truncated (W6*) FIBCD1. Shown are DAPI (blue), anti‐FLAG (green) and merge. Scale bar = 50 μm. Representative of two independent experiments.

To determine whether the germline *FIBCD1* variants identified in P1 and P2 affect protein folding or function, we performed all‐atom MD simulations in the microsecond range of the two *FIBCD1* variants contained within the FReD (p.R406C and p.P456L) and the WT as control. Both WT and patient variant conformations stayed relatively close to the initial structure, with the backbone root‐mean‐square deviation (RMSD) being the highest for R406C, intermediate for P456L and the lowest for WT (Fig EV5A), but never exceeding 2.5 Å. In order to compare WT and the two mutant structures, the dominant MD conformations were identified using structural clustering. The dominant P456L and R406C structures deviated from the dominant WT structure by 1.6 and 1.5 Å backbone RMSD, respectively, while being relatively more similar to each other (1.2 Å). The largest structural rearrangements induced by the variants took place in the 389–399 and 423–448 loop regions, which surround the ligand binding site (Fig 4I). Here, the R406C variant had a direct effect due to a disruption of the salt bridge between R406 and D433, which in the WT likely stabilised the mutual arrangement of the two loop regions. In the case of the P456L variant, the effect was allosteric, whereby perturbation of the conformational dynamics of the C‐terminus, likely due to the removal of the sterically restricted P456, was transmitted towards the upstream 423–448 loop region. Importantly, a similar structural effect of both variants was connected to a similar perturbation of the electrostatic properties on the protein surface in the vicinity of the ligand binding site. In particular, both variants significantly increased the negative charge density of the surface patch surrounding the ligand binding site, in contrast to the WT where the corresponding surface was positively charged (Fig 4I, lower). We hypothesised that this perturbation significantly weakens the binding of negatively charged ligands such as GAGs.

To substantiate these observations, we generated human HEK293T cell lines stably overexpressing FLAG‐tagged human WT FIBCD1 cDNA and each of the patient variants G29S, R406C and P456L, as well as a control W6* variant located in the gnomAD database (Fig EV5B), which generates a premature STOP codon at the 6^th^ amino acid residue of FIBCD1. We confirmed FLAG immunoreactivity in each overexpressing cell line, except W6* (Fig EV5C), and again tested the cells' binding to FITC‐tagged CS‐4S by flow cytometry. Consistent with mFIBCD1 (Fig 4E), we determined that cells expressing hFIBCD1 showed increased FITC^+^ cells relative to unstained controls or cells expressing FIBCD1_W6* negative control (Fig 4J). This was not the case for cells expressing any of the patient variants, which exhibited a similar percentage of FITC^+^ cells as the untransduced control and cells expressing FIBCD1_W6* (Fig 4J). Together, these data suggest that while the *FIBCD1* variants identified in patients did not affect protein expression or folding, they disrupted binding of FIBCD1 to GAGs such as CS‐4S. Further, the molecular docking experiments suggest that the disturbed binding of the R406C and P456L variants may be due to a disruption of the surface electrostatic charge of the CS binding pocket of FIBCD1's FReD. Therefore, we conclude that P1 and P2 harbour variants deleterious to FIBCD1 function.

**Figure 5:**
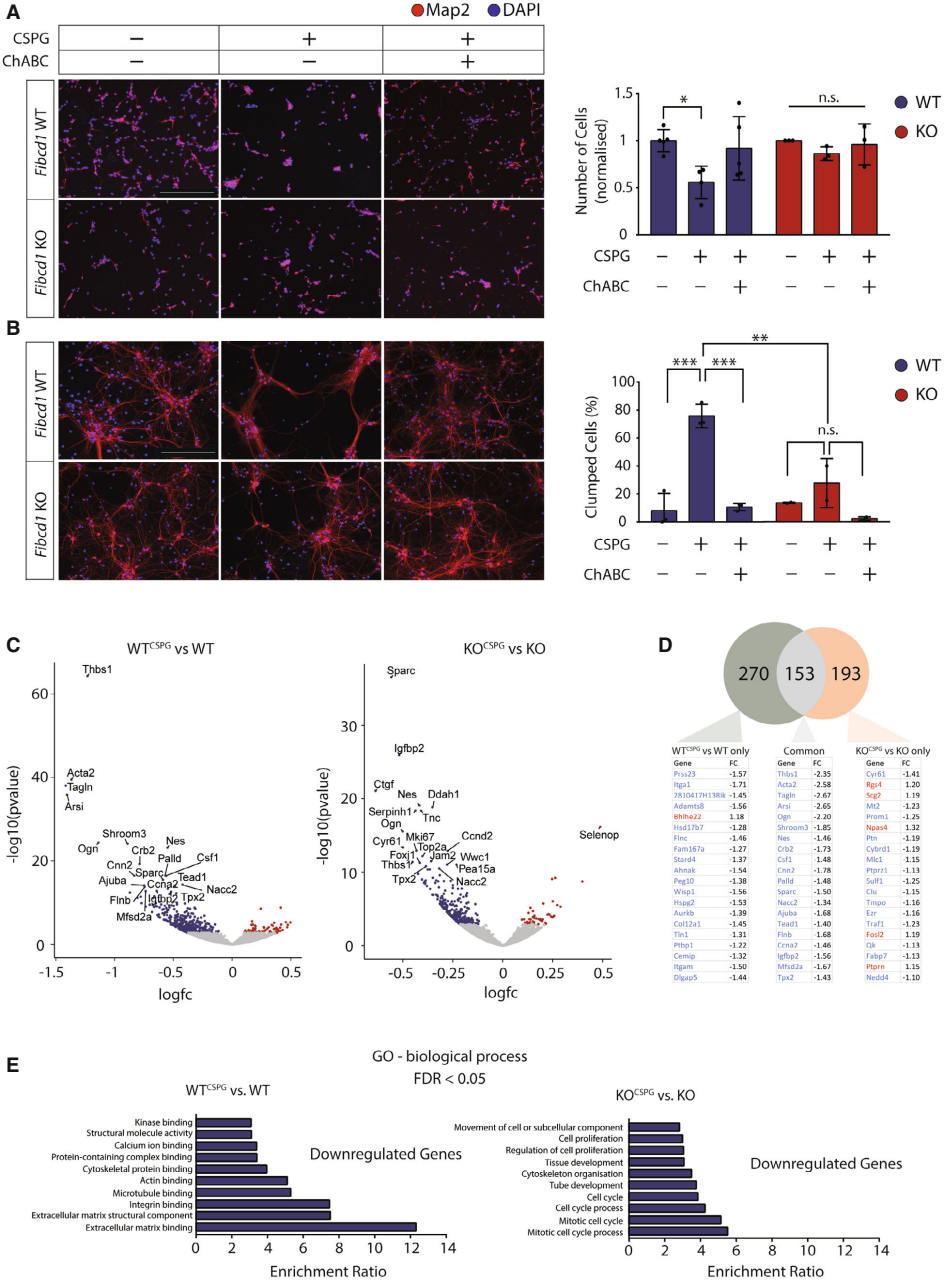
FIBCD1 mediates responses of primary hippocampal cultures to CSPGs ALeft, representative image of immunofluorescent staining (MAP2, red; DAPI, blue) of primary hippocampal cultures at 2 days *in vitro* (DIV), plated on +/− CSPG coating with and without prior digestion with ChABC, as indicated. Right, quantification of DIV2 images, showing the number of protruding cells per field normalised to untreated condition. *n*(*Fibcd1* WT) = 4; *n*(*Fibcd1* KO) = 3. Scale bar = 250 μm.BLeft, representative images of DIV14 neurons, same conditions as in (A). Right, quantification of DIV14 images, representing the percentage of cells per field that are clumped. *n*(*Fibcd1* WT) = 3; *n*(*Fibcd1* KO) = 2. Scale bar = 250 μm.CVolcano plots of differential gene expression of transcriptomes at DIV3 hippocampal cultures comparing (left) WT^CSPG^ vs. WT and KO^CSPG^ vs. KO (FDR < 0.05; right). Significantly upregulated and downregulated genes are shown in red and blue, respectively. The top 20 DEGs are labelled.DAbove, Venn diagram of significant DEGs unique to WT^CSPG^ vs. WT (green, 270 genes), KO^CSPG^ vs. KO (orange, 193 genes) and common between the two (grey, 153 genes). Below, lists of the 20 most significant DEGs and their fold change for each comparison, showing downregulated DEGs in blue and upregulated in red. *n*(*Fibcd1* WT) = 5; *n*(*Fibcd1* KO) = 4. *n* represents a prep of cells.EGO term enrichment analysis for significantly downregulated genes (FDR < 0.05) in (left) WT^CSPG^ vs. WT and (right) KO^CSPG^ vs. KO. Data information: For panels (A and B), each data point represents an individual preparations of primary cell culture. Data are represented as mean, and error bars represent SEM. *P*‐values were calculated by one‐way ANOVA. *P ≤ 0.05; **P ≤ 0.01; ***P ≤ 0.001.

### FIBCD1 mediates GAG signalling in neurons

CS polysaccharides in various sulphated forms (including CS‐4S) are usually found to be conjugated to CSPGs, which have important signalling functions (Gama *et al*, 2006; Smith *et al*, 2015). We sought to determine whether FIBCD1 mediates CSPG signalling in hippocampal neurons. To this end, we plated cultured E18.5 *Fibcd1‐*WT and KO mouse hippocampal neurons on a coating of primary CSPGs, which contain a mixture of sulphated GAGs. At DIV2, we found reduced attachment of WT but not KO neurons on CSPG coatings, which was reversed by cleaving the CS chains with the enzyme ChABC (Fig 5A). Additionally, CSPGs induced aggregation of cultured WT neurons at DIV14 (in agreement with previous literature (Jin *et al*, 2018)), but cultured KO neurons did not aggregate (Fig 5B). These results suggest that FIBCD1 mediates CSPG signalling in cultured hippocampal neurons.

To investigate FIBCD1‐dependent transcriptional responses to CSPGs, we isolated RNA from primary hippocampal neurons plated on coverslips coated with CSPGs (*Fibcd1* WT^CSPG^, *Fibcd1* KO^CSPG^) and without CSPGs (*Fibcd1* WT, *Fibcd1* KO) at DIV3. We performed bulk RNA sequencing with poly‐A enrichment using 4–5 biological replicates per condition. We reasoned an early time point after plating would more likely reflect cellular developmental effects of CSPG‐FIBCD1 signalling rather than secondary effects such as increased cell stress, soma aggregation or dendritic fasciculation. Hierarchical clustering showed small intragroup differences and distinct separation between groups by genotype (WT or KO) and treatment (+/− CSPG; Appendix Fig S1A). Comparison of differentially expressed genes (DEGs, FDR < 0.05) between KO and WT cells (without CSPG) revealed 462 significant DEGs with *Fibcd1* being the most downregulated DEG, as expected (Appendix Fig S1B). We noted that a number of the top enriched DEGs in the KO vs. WT condition to be genes were specifically expressed in non‐neuronal cells (e.g. *Pdgfra*, *Olig2*), suggesting that DEGs may be reflecting differences between WT and KO cultures in numbers of glia, which are technically challenging to control for. We therefore explored our data further comparing only between conditions within the same genotype, i.e. WT^CSPG^ vs. WT and KO^CSPG^ vs. KO, which allowed us to isolate the DEGs dependent on FIBCD1 activity.

Comparison between WT^CSPG^ vs. WT revealed 462 significant DEGs, of which the majority (396) were downregulated and KO^CSPG^ vs. KO revealed 345 significant DEGs, of which the majority (301) were also downregulated (Fig 5C). We cross‐referenced DEGs identified in the WT and KO datasets to reveal a set of genes that are responding to CSPGs in both genotypes and those that are dependent on *Fibcd1* expression (Fig 5D). Gene ontology (GO) term enrichment analysis for downregulated genes in WT^CSPG^ cells revealed terms such as “extracellular matrix binding” and “extracellular matrix structural component” (Fig 5E). Intriguingly, the third‐most enriched term was “integrin binding”, reflecting a number of integrin subunits and integrin‐related genes that are significantly downregulated in WT cells upon CSPG treatment (Appendix Fig S1C). Among the genes dysregulated in response to CSPGs only in the WT cultures are genes coding for integrin subunits (*Itga1*, *Itgam*), integrin binding and/or modulation (*Adamts8, Tln1*; Collins‐Racie *et al*, 2004; Nieswandt *et al*, 2007), genes involved in the synthesis or degradation of ECM components (*Adamts8, Hspg2, Cemip, Col12a1*; Yoshino *et al*, 2018) and, finally, genes involved in binding to the ECM and adhesion of cells to each other and to the ECM (*Flnc, Wisp1, Tln1*; Desnoyers *et al*, 2001; Nieswandt *et al*, 2007; Manso *et al*, 2017; Begay *et al*, 2018; Haage *et al*, 2018). These genes represent the transcriptional fingerprint of primary hippocampal neurons mediated by CSPG‐FIBCD1 interaction and suggest that FIBCD1 both engages with the ECM and facilitates transcriptional regulation of ECM components.

## Discussion

Here, we report deleterious variants in the gene *FIBCD1* in two unrelated patients presenting with undiagnosed neurodevelopmental disorders. *FIBCD1* is a gene of largely unknown function in humans. Accordingly, here we show that *FIBCD1* is highly expressed in human and mouse brain and demonstrate that it binds to and functions as a regulator of glycosaminoglycans of the brain ECM. Further functional characterisation in several animal models demonstrates broad roles in hippocampal synaptic and behavioural function. Together, we propose *FIBCD1* loss‐of‐function variants underlie neurodevelopmental symptoms, at least in part, by disrupting brain ECM content critical for normal neuronal and synaptic functions.

Two patients (P1 and P2) with deleterious variants in *FIBCD1* exhibited symptoms of severe neurodevelopmental dysfunction, including delayed social, cognitive and verbal abilities, ASD, ADHD, facial dysmorphias, delayed sitting and walking milestones and structural brain anomalies. P2 was too young at last examination to be fully evaluated for ASD or ID; however, P1 is more affected than P2. Intriguingly, signs of immune system symptoms such as recurring allergic rhinitis, sinusitis and pneumonia in both patients are in line with the literature describing FIBCD1 in immune responses (Jepsen *et al*, 2018). In addition to *FIBCD1* variants, P2's exome sequencing revealed additional variants of unknown significance in *UNC13B* and *RIC1. UNC13B* encodes a pre‐synaptic protein highly expressed in the brain, MUNC13‐2, that has recently been associated with partial focal epilepsy (Wang *et al*, 2021), which is not a symptom found in P2, and was therefore dismissed as potentially causative in this case. Variants in *RIC1* gene have recently been associated with autosomal recessive CATIFA syndrome marked by cleft lip, cataract, tooth abnormality, intellectual disability, facial dysmorphism and attention‐deficit/hyperactivity disorder (OMIM: 618761; Patel *et al*, 2017; Unlu *et al*, 2020). With the exception of P2's micrognathia, she exhibits none of the other hallmark symptoms of CATIFA syndrome. However, the contribution of the *RIC1* variant to the overall clinical pathology of the patient cannot be ruled out, even if unlikely. While the clinical synopsis of both patients suggests a complex neurodevelopmental disorder, with common symptoms that include delayed cognition, difficulty with language, mild facial dysmorphisms and some respiratory/immune dysfunctions, the patients differ in key aspects of their symptoms. P1 has severe ASD and ADHD including sensory dysfunctions and fine motor deficits. P2 on the contrary is more affected, with structural brain anomalies, including microcephaly, as well as delayed locomotion and sitting abilities. Clinical differences even in monogenic NDDs are common and can be accounted for by various factors, including age, sex and ethnicity. As mentioned above, P2 has additional potentially contributing variants that may explain the severity of her disorder.

FIBCD1 was first identified as a cDNA clone with high homology to ficolins, lectin‐type pattern recognition receptors of the innate immune system (Schlosser *et al*, 2009). It has been shown to assemble into homotetrameric, transmembrane structures and expressed in tissues including the brain, trachea, small intestine and lung mucosal membrane, particularly after fungal infection (Jepsen *et al*, 2018). FIBCD1 binds with high affinity to chitin and mediates the endocytosis of acetylated structures (Schlosser *et al*, 2009). Using a transgenic mouse overexpressing FIBCD1 in intestinal tissues, FIBCD1 was shown to regulate the gut mycobiome content (Moeller *et al*, 2019) and lung immune responses to fungal infection (Bhattacharya *et al*, 2021) presumably through its chitin‐binding properties. Several reports revealed FIBCD1 association with cancer, with its overexpression linked to poor prognosis in gastric cancer (Jiang *et al*, 2018) and hepatocellular carcinoma (Wang *et al*, 2020). A recent study identified FIBCD1 as a myokine regulator of myofiber size in the diaphragm muscle (Graca *et al*, 2022).

We now demonstrate an important role of FIBCD1 in nervous system development and function. We show that knockdown of a putative *FIBCD1* orthologue in flies, *CG10359*, resulted in morphological defects of the neuromuscular junction and corresponding deficiencies in locomotor behaviours. Furthermore, FIBCD1‐deficient mice exhibited impaired performance in hippocampal‐dependent learning tasks. We identified FIBCD1 as a neuronal receptor for GAGs found in the brain ECM, with dysregulation of CS‐4S/‐6S noted in hippocampi of *Fibcd1* KO mice. Importantly, the variants identified in the patients reported here disrupt the association between FIBCD1 and CS‐4S demonstrating they are deleterious to protein function. Further, we found that FIBCD1 mediates neuronal responses to CSPGs and a transcriptional programme associated with cell–cell and cell–matrix interactions. Finally, we found that FIBCD1 deficiency significantly impaired both short‐ and long‐term forms of synaptic plasticity of the kind important for learning and memory deficits that could be fully rescued by enzymatic modulation of the ECM.

While demonstrating some functions of FIBCD1 in the nervous systems of two different animal models, we also observed notable differences between them and with the patient symptomatology. For example, full‐body *dFibcd1* knockdown in *D. melanogaster* was lethal, while *Fibcd1* KO mice were viable and overtly normal in body and brain weights and gross brain structure, possibly suggesting that FIBCD1 has a more specialised role in mammals. Neuronal *dFibcd1* knockdown in *D. melanogaster* resulted in dramatic morphological aberrations; however, only slight morphological changes were noted in hippocampal pyramidal neurons of *Fibcd1* KO mice. Nevertheless, FIBCD1 deficiency leads to specific hippocampal‐dependent learning deficiencies. Other behaviours and neuronal functions in KO mice appeared normal, for example nociceptive, motor or sensory function as were levels of anxiety, unlike the features noted in P1. A recent preprint, for example, reports elevated amygdala levels of *Fibcd1* mRNA in response to fear conditioning in mice (preprint: Reis *et al*, 2021) suggesting a potential role in anxiety yet to be delineated. We also did not detect any structural abnormalities in the brains of the *Fibcd1* KO mice as was noted in P2; however, morphological alterations of the brain were not a shared feature in the two patients. Additionally, microcephaly is often difficult to model in mice; however, in the case of P2, it could also come from additional rare variants. There were also no locomotion deficiencies noted in the mouse model, in contrast to the fly model and P2. As *Fibcd1* is strongly expressed in the hippocampus, we have focused on hippocampal‐dependent learning and identified specific behaviours that are deficient in the KO mouse but not a global dysfunction of this brain region. It would be of great interest to delineate the molecular mechanism that is regulated by FIBCD1 and/or ECM composition for fear conditioning as opposed to spatial learning. We also noted dispersed expression of *Fibcd1* in other brain regions (e.g. cortex and hypothalamus) that may regulate behaviours we did not assay for. While our studies suggest that the genetic variants in the patients would lead to loss of function in ECM binding, it is possible that FIBCD1 has additional, uncharacterised functions that can account for the difference between the KO mice and the patients. Thus, knocking out the gene in mice of flies may not fully model the genetic variants in humans. In spite of these differences, it is clear that FIBCD1 is an important signalling molecule in the nervous system, potentially regulating different molecular pathways between species. As additional cases with deleterious *FIBCD1* variants are reported, it will be of great interest to characterise the extent of the clinical variability we report here.

Molecular modelling analysis has suggested R406C (P1) and P456L (P2) lead to FIBCD1 loss‐of‐function by disrupting the binding pocket's electrostatic charge, diminishing the affinity to its GAG ligand, which is consistent with our cellular assay for FIBCD1:CS‐4S binding. However, it is less clear how the other P1 variant, G29S, disrupts binding of FIBCD1 to CS‐4S. While we find the glycine at this residue is largely conserved among other species, the mouse orthologue contains the same substitution of glycine to serine as in P1. How the function of G29 residue diverges from mouse to human and whether it is important for structural conformation of FIBCD1, targeting or downstream signalling remain to be elucidated. Nevertheless, we demonstrated all three *FIBCD1* variants to be deleterious to protein function of FIBCD1 and in view of the data in the model organisms and cell culture are likely to be causative of the patients' symptoms.

FIBCD1 is an endocytic lectin, previously reported to bind chitin on cellular walls of pathogens and to regulate the innate immune system (Schlosser *et al*, 2009; Moeller *et al*, 2019). We provide evidence that FIBCD1 also has endogenous ligands in the brain and regulates ECM composition through endocytosis, receptor‐mediated signalling, or both. Indeed, transcriptomic changes upon CSPG stimulation of *FIBCD1* WT and KO primary hippocampal cultures reveal a novel ligand‐dependent signalling function for FIBCD1, primarily encompassing genes involved in ECM binding and structure. Consistent with a recent study showing FIBCD1 to regulate expression of integrin subunits in muscle cells (Graca *et al*, 2022), a number of neuronal DEGs were integrin subunits or integrin‐related genes, molecules well known for interacting with the ECM and signalling during neuronal development and synaptic activity (Dityatev & Schachner, 2003; Dityatev *et al*, 2010). Considering that closely related proteins containing FReDs have been shown to directly interact with integrins (Thomsen *et al*, 2011), it is tempting to speculate a physical FIBCD1–integrin interaction. We cross‐referenced the DEGs present in both the WT^CSPG^ vs. WT and KO^CSPG^ vs. KO datasets to identify the genes specifically regulated by FIBCD1 binding to CSPGs. We identified a number of genes coding for integrin subunits or integrin binding and/or modulation, as well as genes involved in the synthesis or degradation of ECM components and, finally, genes involved in binding to the ECM and adhesion of cells to the ECM. While the functions of many of these genes have been elucidated in a non‐neuronal context, it is likely that their function is largely conserved in neurons, and therefore, these genes make up the transcriptional fingerprint regulated by FIBCD1's interaction with CSPGs in primary hippocampal neurons.

The LTP deficits noted in hippocampal circuits likely underly the behavioural learning deficiencies in the mice and could be generalised to synapse function in other brain regions, contributing to some of the clinical symptoms of the patients. The complete rescue of LTP and PPI deficits by ChABC pre‐treatment is interesting, but the mechanism remains elusive. It is tempting to speculate that the increased levels of CS‐4S observed in the KO mouse hippocampi are due to a lack of FIBCD1 endocytic activity over time (therefore CS‐4S intracellular degradation), which is inhibitory to synaptic remodelling required for PPI and LTP, and that digestion with ChABC “restores” CS‐4S abundance to basal levels. However, the fact that the WT slices treated with ChABC have impaired LTP and PPI means that the mechanism is likely more complicated. We additionally demonstrated a signalling role of FIBCD1 in cultured hippocampal neurons in response to CSPGs, identifying a FIBCD1‐dependent transcriptional fingerprint that includes integrins, ECM components and their modifiers, all of which have a well‐established role in healthy and pathologic hippocampal synaptic plasticity (McGeachie *et al*, 2011). The exact mechanism of FIBCD1 regulation of ECM composition and synaptic plasticity is likely dependent on developmental age and brain region and neuronal cell type and remains to be elucidated.

To conclude, FIBCD1 is a receptor for GAGs of the ECM and mediator of ECM signalling, disruptions to which are associated with aberrant synaptic function and likely leading to a complex NDD.

## Materials and Methods

### Patients and whole‐exome sequencing

All procedures were performed following informed consent and approval from patients and relatives and obtained in accordance with the Declaration of Helsinki. The cohort was curated in a collaborative effort and with the aid of GeneMatcher (Sobreira *et al*, 2015).

#### Patient 1

gDNA from the proband and parents was captured using the IDT xGen Exome Research Panel v1.0. NGS using an Illumina system with 100 bp or greater paired‐end reads. Aligned reads (GRCh37) were analysed for sequence variants using a custom‐developed analysis tool. Additional details have been previously described (Retterer *et al*, 2016). The general assertion criteria for variant classification are publicly available on the GeneDx ClinVar submission page (http://www.ncbi.nlm.nih.gov/clinvar/submitters/26957/).

#### Patient 2

Procedures were in accordance with the ethical standards and approval of the Medical Ethics Committee of Peking University First Hospital, IRB number No. [2005]004. Patients were sequenced and analysed as described previously (Yan *et al*, 2021), with sequencing performed by Joy Oriental Co. (Beijing, China).

### Animals

#### Mus musculus

All mice were housed at the Comparative Medicine Mousehouse (Vienna BioCenter, Vienna, Austria). *Fibcd1tm1Lex* mice (MGI: 5007144; Tang *et al*, 2010) were bred on a C57BL/6J genetic background. Only age‐ and sex‐matched littermates from respective crosses were used. All mice were housed at the Institute of Molecular Biotechnology (IMBA, Vienna, Austria), in a 12‐h light/dark cycle, with food and water *ad libitum*. Experiments were approved by the Bundesministerium für Wissenschaft, Forschung und Wirtschaft (BMWFW‐66.009/0048‐WF/V/3b/2018), and carried out according to EU‐directive 2010/63/EU.

#### Drosophila melanogaster

Flies were age‐, light‐, sex‐ and temperature‐matched. All crosses were raised at 25°C on standard molasses food.

### *In situ* hybridisation

Brains were dissected from two 8‐ to 10‐week‐old C57B6J mice, fixed in 4% paraformaldehyde, dehydrated and paraffin‐embedded. 3.5‐μm‐thick frontal sections were *in situ*‐hybridised with an enhanced RNAScope 2.5 high‐definition procedure (310035, ACD Bioscience), as described previously (Lassen *et al*, 2017).

### RT–qPCR

Mouse tissues/cells were collected into TRIzol (Invitrogen), reverse‐transcribed with iScript cDNA synthesis kit (Bio‐Rad) and amplified with GoTaq qPCR master mix (Promega) on a CFX384 system (Bio‐Rad). Data were normalised to *Gapdh*. Human cDNA panels were obtained from OriGene: TissueScan, Human Brain cDNA Array (#HBRT101) and Human Normal cDNA Array (#HMRT304). Statistics were calculated by one‐way ANOVA.

### *In Silico* modelling of FIBCD1

#### Docking solutions

*In silico* docking was performed using GOLD version 5.2.2 (Jones *et al*, 1997) and the FReD X‐ray structure (PDB: 4M7F; aa 239–458; Shrive *et al*, 2014). The post‐rescoring of docking solutions (100 in total) was done as described previously (Ribeiro Ede Jr *et al*, 2014). The binding free energy of CS‐4S and CS‐6S to FReD was estimated using PRODIGY‐LIGAND (Kurkcuoglu *et al*, 2018) after complex refinement using HADDOCK2.2 web server (van Zundert *et al*, 2016).

#### Patient variant simulations

The initial protein configuration was taken from the FReD X‐ray structure with R406C and P456L variants introduced using PyMOL (Schrodinger, 2015). The structures were subjected to all‐atom molecular dynamic (MD) simulations in the microsecond range using GROMACS 5.1.4 (Abraham *et al*, 2015) and Amber99SB‐ILDN force field (Lindorff‐Larsen *et al*, 2010) as described previously (Sponga *et al*, 2021), with the following differences: box‐size = 6 × 6 × 6 nm^3^, TIP3P water (Jorgensen, 1981) and no position restraints during production run. Root‐mean‐squared deviations (RMSD) from the starting configuration were calculated over backbone atoms (GROMACS *rms* utility). Conformational clustering (GROMACS *cluster* utility) was performed with the backbone RMSD cut‐off for neighbouring structures of 0.9 Å—a minimum value at which only a single dominant state was identified for WT. Electrostatic potential was calculated and mapped onto the protein solvent.

#### Evolutionary conservation

All existing full‐length FIBCD1 structures in AlphaFold Protein Structure Database (https://alphafold.ebi.ac.uk/search/text/FIBCD1; April 2022) were analysed. These correspond to proteins from vertebrate organisms, including human (*Homo sapiens*), macaque (*Macaca fascicularis*), mouse (*Mus musculus*), rat (*Rattus norvegicus*), fish (*Danio rerio*), and frog (two proteins from *Xenopus laevis* and one protein from *Xenopus tropicalis*). A *Drosophila melanogaster* FIBCD1 full‐length structure was modelled *de novo* using the ColabFold framework (preprint: Mirdita *et al*, 2022) for running AlphaFold2 and the corresponding protein sequence (UniProt ID: B7Z0B3). Structure prediction by AlphaFold2 (AlphaFold2‐ptm) was performed using the pdb template mode (pdb70 database) and a subsequent relaxation in Amber. For multiple‐sequence alignment (MSA), MMseqs2 (https://mmseqs.com) was used. Other parameters were set to their default values in ColabFold.

The final set used for the analysis consisted of seven full‐length FIBCD1 AlphaFold structures, whereby the two proteins from *X. laevis* were omitted in order to include a single structure for each evolutionary branch. Sequence ranges corresponding to the FRED domain in each protein were taken from UniProt. Phylogenetic trees based on MSA distances were generated for the final protein set using a stand‐alone version of Clustal Omega (Sievers *et al*, 2011). Structural alignment of FRED domains and calculations of the corresponding root‐mean‐square deviation (RMSD) matrix for backbone atoms were performed in PyMOL using the *align* function. MSA‐ and RMSD‐derived trees were visualised using T‐REX server (Schrodinger, 2015). Structures of FRED/CS complexes for all proteins were built using a structural alignment of the FRED domain against the previously obtained complexes of the human FRED and CS‐4S/6S. Relaxation of complex structures and estimation of electrostatic interaction energies were carried out using HADDOCK 2.2 web server (van Zundert *et al*, 2016). Final estimation of ΔG values for each complex using the corresponding HADDOCK electrostatic energies was done using PRODIGY‐LIGAND (Kurkcuoglu *et al*, 2018).

### Binding assays

Characterisation of FIBCD1 binding specificity to CS‐4S and CS‐6S was performed through ELISA‐based inhibition experiments as described previously (Schlosser *et al*, 2009). Statistics were calculated by Student's *t*‐test.

### MRI

Male mice (12 months) were anaesthetised with 1.5% isoflurane and imaged in the Preclinical Imaging Facility at VBC Facilities with a 15.2 T MRI (Bruker BioSpec, Ettlingen, Germany) and BFG6S‐100 actively shielded gradient system (1 T/m maximum gradient strength). Four‐channel receiver coil (Bruker BioSpin) was used. A T1‐weighted multi‐slice multi‐echo (MSME) 3D sequence was used with TR/TE 50/8 ms, 1.8 × 1.2 × 0.8 cm^3^ field of view, 50 × 50 × 50 μm^3^ spatial resolution and 16 averages. 3D reconstruction was generated by manually segmenting each image using Amira 5.6 (Visualization Science Group). The delineation of different brain structures was performed in the axial plane and subsequently controlled in the two other planes. Paxinos mouse brain atlas was used as a reference (Paxinos & Watson, 2006). The brain surface and structures were delineated based on the MRI signal intensity differences. Values were averaged, and unpaired Student's *t*‐test was used to determine statistical significance.

### Behavioural assays

#### *Drosophila* negative geotaxis assay

Female *Nsyb‐Gal4* animals were crossed with *UAS‐RNAi* lines targeting *CG10359*. Female offspring were tested at 10 days after eclosion. Flies were knocked out with CO_2_, sorted into batches of 3–7, recovered for 25 h, flipped into empty vials and given 10–15 m to recover. The climbing index is the percentage of flies that pass the 5‐cm mark in 5 s after gently tapping to the bottom of a vial. Statistics were calculated by one‐way ANOVA.

#### Mice

All experiments were conducted using C57BL/6 mice at the pcPHENO, VBCF. Experiments were performed in accordance with the ARRIVE guidelines. Female and male mice were included in the trials, with no sex differences noted for any tests. Exclusion criteria for all assays were specified *a priori*; however, no animal was excluded.

Elevated Plus Maze was performed as described previously using an automated activity system (TSE‐Systems; Nagy *et al*, 2019). Briefly, mice were placed in the centre of a grey “plus”‐shaped plexiglass arena, consisting of two‐walled arms, and two open arms. Exploratory behaviour of mice was recorded over a 5‐min period. Distance travelled and average velocity during the test were compared with controls and used as a readout for locomotion deficits. Unpaired Student's *t*‐test was used to determine significance.

Morris water maze was performed as described previously (Nagy *et al*, 2019). Briefly, mice were trained to swim in a pool with opaque water to find a submerged platform using the visual cues for orientation. Mouse performance was video‐tracked using the software Topscan 3.0 (Cleversys Inc., VA, USA). On Day 1, the visual capacity and swim speed were recorded by allowing mice 1 min of exploration time to seek a visible platform. Coordinated swim movements and latency to reach the platform were recorded to inform about visual acuity and locomotion capacities of the mice. Mice were then trained to find the hidden platform for eight trials for 5 consecutive days. Short‐term memory probe test was performed after the last trial, on Day 8, without the platform for 1 min. The same trial was repeated in the morning of Day 11 to test for long‐term memory. Unpaired Student's *t*‐test was used to analyse the short‐ and long‐term memory data and two‐way ANOVA with Sidak's multiple comparison for the latency to reach the platform.

Y‐Maze consisting of a T‐shaped apparatus, where each walled arm partitioned by removable guillotine doors, was performed as described previously (Deacon & Rawlins, 2006). Briefly, animal is placed in different arms for each of the seven trials, and their choice to alternate goal arms was manually scored. Statistics were calculated by one‐sample *t*‐test and unpaired Student's *t*‐test.

Inhibitory avoidance was performed in a custom‐made apparatus consisting of two chambers separated by an automatic door. The start chamber is white‐walled and brightly lit; the other is dark‐walled with an electrified grid. The animal received a 2‐s 0.3 mAmp foot shock from the grid once it entered the dark chamber. The latency to enter the cark compartment from the start chamber 24 h later is manually recorded and interpreted as memory of the foot shock. Animals were trained only once. Unpaired Student's *t*‐test was used to determine significant difference between the cohorts.

Hot plate assay was performed by placing the animal on a hot plate (Ugo Basile) at 50°C and next day at 52°C and manually observed for first reaction for a maximum of 60 s. Counted reactions included the following: jumping, licking, shaking or lifting of the hind paws. For reaction to capsaicin, 1 μg of capsaicin (Sigma; M2028) diluted in 15 μl PBS was injected intraplantar in the hind paw and animal was observed for 5 min and timed for duration of the reactions described above. Reaction to acetone was recorded as duration of cumulative licking or biting of the hind paw following acetone application three times at intervals of 30 s. Unpaired Student's *t*‐test was used to determine significant difference between the cohorts.

For the accelerating rotarod performance, mice were first given a 1‐min pre‐trial with no rotation on the rotating rotarod apparatus (Ugo Basile). Next, mice were challenged with two trials of 4 rpm rotation for 1 min. Finally, mice were given four trials with 4–40 rpm acceleration of the rotation for up to 5 min or until the mouse fell off or showed passive rotation without walking. Latency to fall off and speed reached were automatically recorded. Significance was determined by two‐way ANOVA with Sidak's multiple comparison.

### HPLC

CS was extracted from defatted, pronase‐digested, microdissected hippocampi (CA1) and digested using ChABC (Sigma‐Aldrich #C3667). The resulting GAGs were labelled with 2‐aminobenzamide by reductive amination and analysed as described previously (Takegawa *et al*, 2011). Identity of glycosaminoglycan‐derived disaccharides was inferred from retention time alignment with the major constituents of CS sodium salt from shark cartilage (Sigma‐Aldrich, C4384), and bovine trachea (Sigma‐Aldrich #C9819).

### Tissue culture

#### Cell lines

HEK293T and N2a cell lines were obtained from ATCC: CRL‐3216, CCL‐13. They were regularly checked for mycoplasma but not authenticated by STR profiling. They were maintained in DMEM (Sigma #D5796) supplemented with 10% FCS (Sigma #F0804), pen/strep (Biowest #L0022) and sodium pyruvate (Thermo Fisher #11360070).

#### Primary neurons

E18.5 pups were sacrificed and hippocampi dissected into Hank's buffered saline solution (HBSS, Gibco #14185045). The tissue was minced, trypsinised (0.025%) and triturated with heat‐polished glass pipettes. Plating was in Neurobasal medium (Thermo Fisher #21103049) with 10% FCS, 2 mM L‐glutamine (Gibco #25030149), B27 (Gibco #17504001), 10 mM HEPES (Gibco #15630056) and penicillin/streptomycin. Fifty percent of media was exchanged to FCS‐free medium after 24 h and then every 36 h. CSPG (Merck #CC117) coatings were performed as described previously (Shen *et al*, 2009; Jin *et al*, 2018).

### FIBCD1 overexpression

*mFibcd1* cDNA with 3' V5 tag G‐blocks (IDT) was cloned with ΔFReD construct using XhoI‐EcoRI restriction enzymes into a custom pMSCV‐IRES‐mCherry plasmid. Q5 site‐directed mutagenesis kit (NEB #E0554) was used to introduce point variants. N2a cells were lentivirus‐transduced and FACS‐sorted for mCherry^+^ cells. *hFIBCD1* cDNA (OriGene #RC206180) was subcloned by Gateway cloning into a custom plasmid (via pDONR201) with 3' 3xFLAG tags and blasticidin resistance. HEK293T cells were lentivirus‐transduced and selected with 14 μg/ml blasticidin.

### Western blot

Hippocampi were homogenised in ChABC buffer (40 mM Tris–HCl, pH 8.0, 40 mM sodium acetate) containing Benzonase and Halt protease/phosphatase inhibitors (Thermo Scientific) and pelleted, and the supernatant containing soluble protein fraction was separated from the pellet (insoluble fraction), which was resuspended in ChABC buffer. One aliquot of each fraction was incubated with ChABC for 12 h at 37°C, then heated for 5 min at 95°C, separated by SDS–PAGE and transferred onto PVDF membranes. Blocking was for 1 h with 5% milk in TBST overnight at 4°C with primary antibodies (1:100, anti‐CS‐0S, 1B5; anti‐CS‐4S, 2B6, antiCs‐6S, 3B3; Amsbio). Blots were washed 3 × 5 min in TBST and incubated with HRP‐conjugated secondary anti‐mouse‐IgG‐H&L chain or anti‐rabbit‐IgG‐F(ab')2 (GE Healthcare) antibody for 1 h at RT, washed 3 × 5 min in TBST and visualised.

### Immunoprecipitation

N2a cells expressing mFIBCD1, FIBCD1ΔFReD or empty vector were washed twice with PBS and lysed in Hunt buffer (20 mM Tris–HCl, pH 8.0, 100 mM sodium chloride, 1 mM EDTA, 0.5% NP‐40) with Halt protease/phosphatase inhibitors (Thermo) in 3 consecutive freeze and thaw steps, and pelleted, and the supernatant was collected. Lysates were precleared for 1 h with magnetic Protein G Dynabeads (Invitrogen) and immunopurified with anti‐V5 agarose beads (Sigma) overnight at 4°C. After five washing steps in Hunt buffer, input and immunoprecipitation samples were separated by SDS–PAGE, blotted and stained with anti‐V5 antibody (ab15828, 1:2,000 dilution), and Western blotting was performed as described above.

### Microscopy

At DIV2 and DIV14, primary neurons were PBS‐washed, fixed in 4% PFA (+4% glucose) for 10 min at RT, then quenched with 10 mM glycine/PBS for 10 min at RT. After 2× 0.01% Triton‐X/PBS (PBST) washes, permeabilisation was with 0.25% Triton‐X/PBS for 3 min and blocked in 5% goat serum for 1 h. Primary antibodies were incubated overnight at 4°C and washed 3× in PBST. Secondary antibodies were incubated for 1 h at RT and washed 3× with PBST before mounting. Eight to 10 semi‐random fields were acquired per coverslip at 40× magnification. During analysis, experimenters were blinded to condition (i.e. +/− CSPG) and genotype (WT vs. KO), and the number of MAP2^+^ clumped cells (~10 soma clustered together) was calculated as a percentage of all MAP2^+^ cells in an image. DIV2 quantifications are reported normalised to the untreated control; for DIV14, the number of clumped MAP2^+^ cells is reported as a percentage of all MAP2^+^ cells per field. All MAP2^+^ cells in each image were included for analysis, which was usually in the range of 100–300 cells.

For HEK293T FLAG staining, cells were PBS‐rinsed and fixed in 4% PFA (4% glucose) for 10 min at RT, then 10 min with PBS (10 mM glycine). The cells were washed 2× with PBS, permeabilised with 0.25% Triton‐X/PBS, blocked for 1 h with 5% goat serum and incubated with primary antibodies (anti‐FLAG, 1:1,000) overnight at 4°C, and washed 2×, and then, secondary antibodies Alexa Fluor (1:500) and DAPI (1:2,000) were added for 1 h at RT, washed again 2×, then mounted and imaged on a Zeiss LSM980.

For CS‐4S internalisation, cells were seeded in black CellCarrier Ultra Microplates (Perkin Elmer) and, next day, incubated with 100 μg/ml FITC‐tagged 4‐O‐sulphated CS (Amsbio #AMS.CSR‐FACS‐A1), diluted in PBS (0.8 mM CaCl_2_) and incubated at 37°C for 45 min. The cells were then fixed in 4% PFA for 10 min, washed and further stained with CellMask Orange Plasma Membrane Stain (Invitrogen #C10045, 1:3,000) and Hoechst (Invitrogen #H3570, 1:2,000) for 10 min at 37°C. The cells were washed again and imaged on an Opera Phenix (Perkin Elmer). Approximately 50 fields per well were acquired in a fully automated fashion, and there were approximately 30–50 cells per field on average. Images were analysed with a custom analysis pipeline in the Harmony analysis software (PerkinElmer). Briefly, the pipeline used the “Find nuclei” module to identify nuclei in the blue (Hoechst) channel, followed by cell boundary segmentation with the “Find cytoplasm” module using the red (CellMask) channel. Next, the pipeline masked the images, setting all pixels outside of the segmented cells to black. Finally, the pipeline used the “Find spots” module to identify CS‐4S puncta in the green (FITC) channel, which were quantified. Importantly, the masking step ensured that only CS‐4S puncta within cells were quantified. For all of the aforementioned imaging experiments, one‐way ANOVA was used to calculate statistical significance.

For co‐localisation experiments, HEK293T‐mCherry‐FIBCD1 cells were incubated with 100 μg/ml CS‐4S and 100 nM LysoTracker Deep Red (Thermo #L12492) for 45 min in the incubator. The cells were fixed and acquired as before.

For Golgi‐cox staining, 5 *Fibcd1* WT and 5 KO adult mouse littermate brains were impregnated and stained with the FD Rapid GolgiStain Kit (FD NeuroTechnologies, Inc. **#** PK401) according to the manufacturer's instructions. Using a vibratome (Leica VT1000S), 100‐μm sections were generated and mounted on slides and with coverslips. Olympus BX51 microscope was used to generate 40× brightfield images of 1 CA1 region pyramidal neuron per brain that were manually traced in the Neurolucida software, version 9 (MBF Bioscience). The Neurolucida Explorer (MBF Bioscience) software was used to analyse number of dendrites, nodes and total length of apical and basal dendrites. Sholl analysis (Neurolucida Explorer) was performed to analyse number of intersections per 20 μm concentric circles starting from the centre of the cell body. For spine density analysis, images were taken on a Axio Imager.Z2 microscope with a 63× magnification using Zen Blue software (ZEISS microscopy). Two apical dendrite branches of 40–60 μm length per mouse (*n* > 3) were manually counted and averaged using the Imagej/Fiji software (Schindelin *et al*, 2012). For Sholl analysis, significance was calculated by two‐way ANOVA and differences at individual distances in the Sholl analysis were corrected for multiple comparisons by Bonferroni's multiple comparisons test. For all other morphological parameters, Student's *t*‐test was used.

### Flow cytometry

N2a cells expressing mFIBCD1‐V5, mFIBCD1‐V5^ΔFReD^ or empty vector were washed once with PBS and incubated for 4 h with 100 μg/ml labelled GAGs: 4‐O‐sulphated CS (AMS.CSR‐FACS‐A1, AMSBIO), polysulphated CS (AMS.CSR‐FACS‐P1) or dermatan sulphate (AMS.CSR‐FADS‐B1) in DMEM. Cells were collected and acquired on FACS LSRFortessa (BD). The experiment was performed in three independent replicates.

HEK 293Ts expressing 3xFLAG‐tagged hFIBCD1, hFIBCD1_W6*, hFIBCD1_G29S, hFIBCD1_R406C, and hFIBCD1_P456L were seeded and, next day, washed with PBS, trypsinised, pelleted and resuspended in 10 μg/ml 4‐O‐sulphated chondroitin sulphate (Amsbio) in fresh PBS (0.8 mM CaCl_2_), and incubated at 37°C for 45 min. Samples were washed in ice‐cold PBS (0.8 mM CaCl_2_) and acquired on LSRFortessa Cell Analyser (BD). The experiment was performed in two independent replicates and analysed by FlowJo v10.6.1 (FlowJo LLC). For statistics, one‐way ANOVA with multiple comparisons was used.

### RNA sequencing

RNA was isolated using RNeasy Mini Kit (Qiagen #74104). Library prep, sequencing and alignment were done at the VBC NGS Facility (Austria), with poly‐A enrichment and sequencing on an Illumina HiSeq 3000/4000, 50 bp single‐read. DESeq2 package (Love *et al*, 2014) was used to identify DEGs, excluding pseudogenes and allosome‐located DEGs. Galaxy web platform (Jalili *et al*, 2020) and WebGestalt (Liao *et al*, 2019) over‐representation analysis method were used for data analysis.

### Acute hippocampal slice preparation and electrophysiological recordings

Memory‐related synaptic plasticity and electrophysiological recordings were studied *ex vivo* in hippocampal slices as previously described (Simon *et al*, 2001; Rammes *et al*, 2009; Kim *et al*, 2012; Monje *et al*, 2012; Cicvaric *et al*, 2016, 2018). Mouse brains were rapidly extracted and immersed in a frosty artificial cerebrospinal fluid solution (aCSF) containing (in mM): 125 NaCl, 2.5 KCl, 25 NaHCO_3_, 2 CaCl_2_, 1 MgCl_2_, 25 d‐glucose and 1.25 NaH_2_PO_4_ (all from Sigma‐Aldrich). aCSF was continuously bubbled with a mixture of 95% O_2_ and 5% CO_2_. 300‐μm slices were transferred to a submerged recovery chamber and rested for > 1 h submerged in aCSF at 30 ± 2°C. For enzymatic treatments, slices were transferred to chambers containing aCSF (0.1% BSA) with 0.2 U/ml of either Penicillinase (Pen; Sigma‐Aldrich, #61305) or Proteus vulgaris chondroitinase ABC (ChABC; Sigma‐Aldrich, #C3667), and incubated for 2 h at 37°C. Slices were then rinsed with aCSF (32 ± 1°C) and transferred to a recovery chamber. The CA3‐CA1 Schaffer collateral pathway was stimulated electrically via a home‐made bipolar tungsten electrode insulated to the tip (50 μm tip diameter) and using an ISO‐STIM 01D isolator stimulator (NPI Electronics, Tamm, Germany). Evoked field excitatory postsynaptic potentials (fEPSPs) were recorded at the CA1 area using aCSF‐filled glass micropipettes (2–4 MΩ) located about 400 μm away from the stimulating electrode. Input/output curves were obtained by delivering increasing pulses of voltage (100 μs in duration) between 0 and 9 V with a delta of 1 V and 10 s between pulses. The strength of synaptic transmission was determined in each case from the decaying slope of recorded fEPSPs. For paired‐pulse‐induced synaptic facilitation, two pulses of voltage with a strength eliciting 40% of the maximum inducible fEPSP amplitude as determined by input/output measurements (40% fEPSP_max_) were delivered at variable interpulse intervals ranging between 20 and 100 ms with a delta increment of 20 ms (pulse pairs delivered every 10 s). The decaying slopes of the evoked fEPSPs for each consecutive pair of pulses were measured, and the strength of synaptic potentiation was determined from the 2^nd^/1^st^ fEPSP slope ratio. To study long‐term potentiation, basal synaptic transmission (baseline) was examined for at least 20 min by recording stable fEPSPs in response to 40% fEPSP_max_ stimulating voltage pulses (100 μs duration; fEPSPs elicited at 0.03 Hz). After recording a steady baseline, a theta‐burst stimulation (TBS) protocol was applied, consisting of five trains of 40% fEPSP_max_ stimulating voltage pulses at 100 Hz (100 μs/pulse, with 4 s intertrain interval). Postsynaptic signal in response to baseline stimulating conditions was measured for 35–70 min as indicated in figure legends. Synaptic potentiation was determined by examining the temporal course of the decaying fEPSP slopes following TBS, normalised to baseline values. Data from fEPSP slopes attained when measuring long‐term potentiation were averaged for every 2 min. All recordings were made using an AxoClamp‐2B amplifier (Bridge mode) and a Digidata‐1440 interface (Axon Instruments). Data (5–22 slices/condition) were analysed using the pClamp‐10 Program software (CA/Molecular Devices, USA). Statistics were calculated by two‐ or three‐way ANOVA where appropriate and *P*‐values adjusted by Tukey's multiple comparisons test.

## Author contributions

**Christopher W Fell:** Conceptualization; data curation; formal analysis; investigation; visualization; writing – original draft; writing – review and editing. **Astrid Hagelkruys:** Data curation; formal analysis; investigation; visualization; methodology; writing – review and editing. **Ana Cicvaric:** Data curation; formal analysis; investigation; visualization. **Marion Horrer:** Data curation; formal analysis; investigation. **Lucy Liu:** Data curation; formal analysis; investigation; visualization. **Joshua Shing Shun Li:** Data curation; formal analysis; investigation; visualization. **Johannes Stadlmann:** Data curation; formal analysis; investigation; visualization. **Anton A Polyansky:** Data curation; formal analysis; investigation; visualization. **Stefan Mereiter:** Data curation; formal analysis; investigation; visualization. **Miguel Angel Tejada:** Data curation; formal analysis; investigation; visualization. **Tomislav Kokotović:** Data curation; formal analysis. **Venkat Swaroop Achuta:** Investigation. **Angelica Scaramuzza:** Investigation. **Kimberly A Twyman:** Data curation. **Michelle M Morrow:** Data curation; investigation; visualization. **Jane Juusola:** Data curation; investigation; visualization. **Huifang Yan:** Data curation; investigation. **Jingmin Wang:** Data curation; investigation. **Margit Burmeister:** Supervision. **Biswa Choudhury:** Investigation. **Thomas Levin Andersen:** Investigation; visualization. **Gerald Wirnsberger:** Investigation. **Uffe Holmskov:** Resources; supervision. **Norbert Perrimon:** Resources; supervision. **Bojan Žagrović:** Resources; supervision. **Francisco J Monje:** Resources; supervision. **Jesper Bonnet Moeller:** Resources; data curation; formal analysis; supervision; investigation; visualization. **Josef M Penninger:** Resources; supervision; funding acquisition. **Vanja Nagy:** Conceptualization; resources; data curation; formal analysis; supervision; funding acquisition; investigation; visualization; writing – original draft; project administration; writing – review and editing.

In addition to the CRediT author contributions listed above, the contributions in detail are:

VN and CWF conceptualised the study. CWF, AH, AC, LL, JSSL, MAT, TK, VSA, MB, BC, SM, JS, AAP, AS, MMM, JJ, JBM and VN performed formal analysis. JMP and VN acquired funding. CWF, AH, AC, LL, JSSL, MAT, MH, SM, JS, AAP, AS, KAT, HY, JW, TLA, GW, JBM and VN investigated the study. NP, BŽ, FJM, JBM, JMP and VN provided resources. UH, NP, BŽ, FQM, JBM, JMP and VN supervised the study. CWF, AH, AC, MH, LL, JSSL, SM, MAT, JS, AAP, JBM and VN visualised the study. CWF and VN wrote the manuscript with contributions from all authors.

## Disclosure and competing interests statement

JJ and MMM are employees of GeneDx, Inc.

**The paper explained**

**Problem**

The extracellular matrix (ECM) of the brain shapes development and regulates a wide range of functional processes in adult life. Mechanisms of ECM signalling are poorly understood as is the relationship of ECM dysregulation to human disease.

**Results**

Through whole exome sequencing, we have identified two unrelated patients with undiagnosed neurodevelopmental disorders harbouring variants of unknown significance in the gene FIBCD1, which has no known function in the central nervous system. Using knockdown fly and knockout mouse models, as well as a variety of *in silico* and in vitro experimental techniques, we have shown that FIBCD1 is a receptor for the ECM. Specifically, FIBCD1 binds and facilitates the endocytosis of an ECM glycosaminoglycan, chondroitin sulphate, which was disrupted by the variants identified in the patients. Disruption of FIBCD1 led to morphological disruptions at the neuromuscular junction in flies and motor‐related behavioural deficits. We show in humans and mice that FIBCD1 is primarily expressed in the hippocampus. KO mice exhibited impaired performance in hippocampal‐dependent learning tasks. Synaptic remodelling in acute hippocampal slices was also disrupted but rescued by enzymatic modulation of the ECM content.

**Impact**

Through a patient‐led study, further elucidated the workings of the ECM as it relates to development and functioning of the brain. Using cellular and organismal models, we have provided experimental evidence of three FIBCD1 variants being loss of function and associated to a neurodevelopmental disorder.

## Supporting information




Appendix

Expanded View Figures PDF

## Data Availability

RNA‐seq data are available on Gene Expression Omnibus GSE201289 (https://www.ncbi.nlm.nih.gov/geo/query/acc.cgi?acc=GSE201289).
