# Supplementary material for: FIBCD1 is an endocytic GAG receptor associated with a novel neurodevelopmental disorder
Source: EMBO Mol Med. 2022 Aug 2;14(9):e15829. doi: 10.15252/emmm.202215829 (PMC9449597; doi:10.15252/emmm.202215829)

APPENDIX

Table of Contents:

Appendix Figure S1

Appendix Figure S1: **Additional RNA-seq analysis**

(A) z-score hierarchical clustering for each sample in *Fibcd1* KO vs WT, WT^{CSPG} vs WT and KO^{CSPG} vs KO. Colours represent scaled expression values, with blue for low and red for high expression levels. Z-score is indicated in the legend. (B) Volcano plot depicting DEGs at DIV2 hippocampal cultures, comparing *Fibcd1* KO vs WT, showing significantly upregulated (red) and downregulated (blue) genes. Top 20 DEGs are labelled. (C) Volcano plot depicting DEGs at DIV2 hippocampal cultures comparing WT^{CSPG} vs WT, all genes that fall into "integrin binding" GO term category are labelled.

Appendix Figure S1

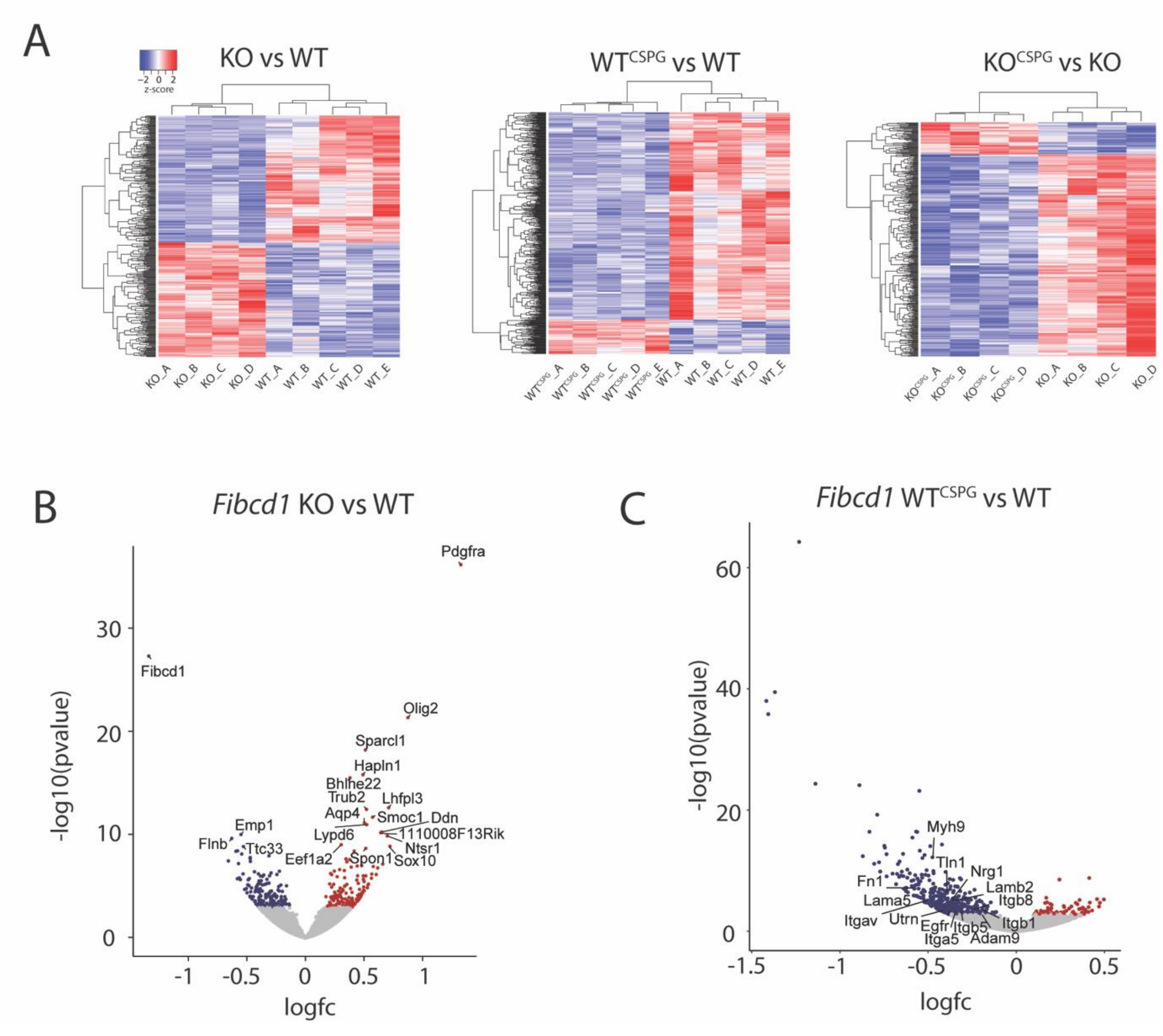

Supplement: Supplementary file 1 — Appendix [file EMMM-14-e15829-s003.pdf]
