# Supplementary material for: FIBCD1 is an endocytic GAG receptor associated with a novel neurodevelopmental disorder
Source: EMBO Mol Med. 2022 Aug 2;14(9):e15829. doi: 10.15252/emmm.202215829 (PMC9449597; doi:10.15252/emmm.202215829)
Supplement: Supplementary file 2 — Expanded View Figures PDF [file EMMM-14-e15829-s001.pdf]

Expanded View Figures

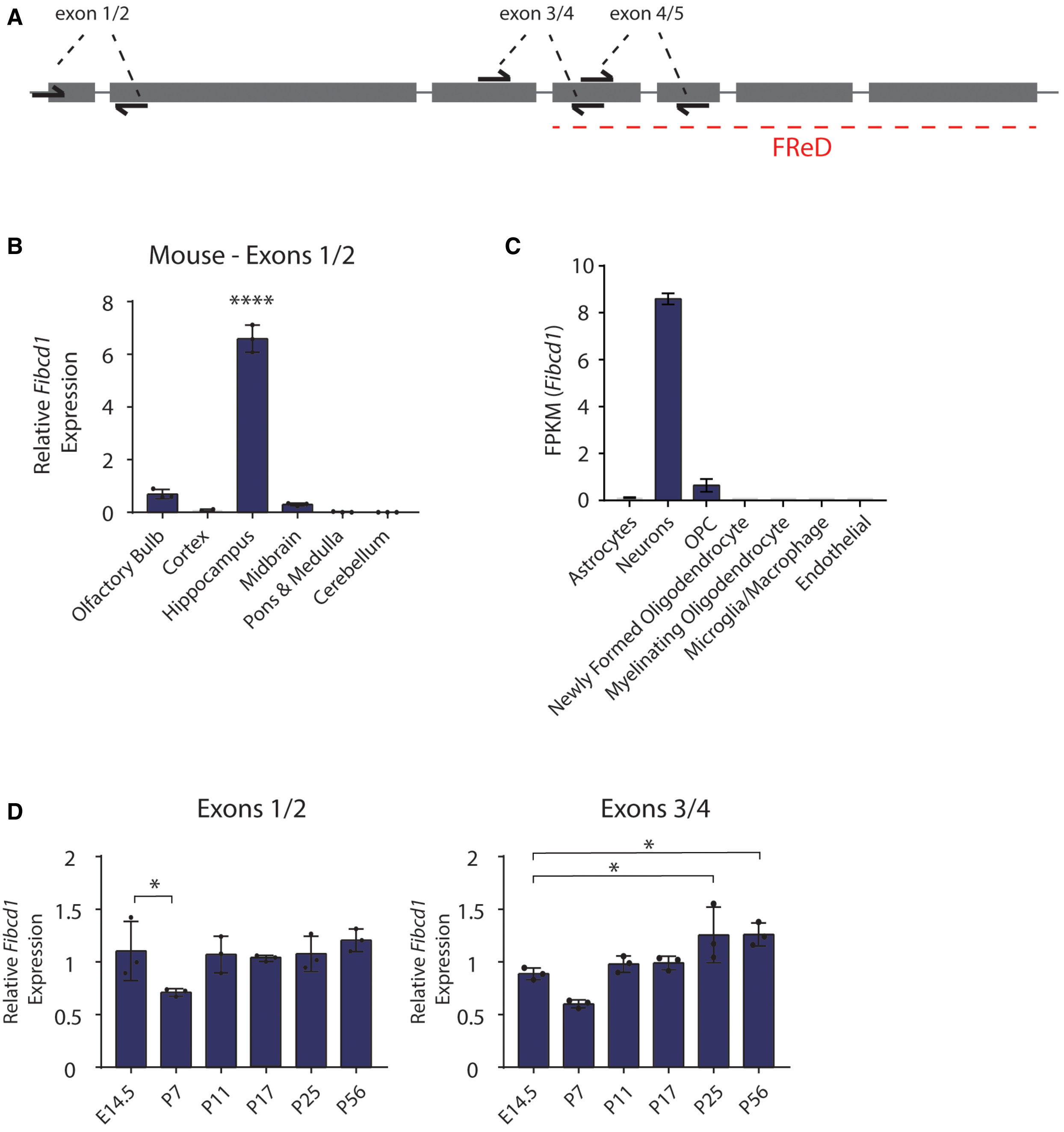

Figure EV1.

 © 2022 The Authors

Figure EV1. *Fibcd1* expression in the adult and developing mouse brain.

A Schematic of mouse *Fibcd1* exons (grey rectangles), introns (grey lines) and location of primer pair binding ("exons 1/2, 3/4 and 4/5") used for RT–qPCR. Exon sizes are to scale; introns and primers are not. The exons coding for FIBCD1 FReD is indicated by a red dashed line.
B Relative mRNA expression levels of mouse *Fibcd1* (primers binding to exon 1 and 2) normalised to *Gapdh* in the indicated brain regions, analysed by RT–qPCR ($n = 3$).
C *Fibcd1* expression in bulk populations of sorted mouse brain cell population, from brainrnaseq.org. OPC, oligodendrocyte precursor cell.
D Relative mRNA expression levels of mouse *Fibcd1* (primers binding to exons 1 and 2 and exons 3 and 4) normalised to *Gapdh* in the hippocampus of the indicated time points, analysed by RT–qPCR ($n = 3$).

Data information: For panels (B and D), each data point represents an individual mouse. Data are presented as mean, and error bars represent SD. *P*-values were calculated by one-way ANOVA comparing each sample with the hippocampus region (B) or the time point E14.5 (D). *$P < 0.05$; ****$P < 0.0001$.

Figure EV2. Description of *dFibcd1*.

A Alignment of fly (DROME), human and mouse FReD protein sequences. Inset shows percent identity matrix (% homology) between fly, human and mouse FReD protein sequences.
B Phylogenetic trees based on multiple-sequence alignments of either full-length FIBCD1 (upper) or FReD amino acid sequences (lower) for the six species with available AlphaFold structures and *D. melanogaster* (AlphaFold structure predicted *de novo*).
C Upper, 3D structures of FReD as predicted by AlphaFold for human (*H. sapiens*, blue), mouse (*M. musculus*, grey) and fly (*D. melanogaster*, pink) with CS-4S (left) and CS-6S (right) docked to the human variant. The average pairwise backbone RMSD and standard deviation over all possible pairs chosen from among the seven species studied are indicated above the structures. The average predicted binding free energies and standard deviations between FReD and CS-4S (left) or CS-6S (right) over all seven species studied are given below the structures. Lower, distributions of AlphaFold predicted local distance difference test (pLDDT) scores for predictions of either full-length FIBCD1 or FReD alone pooled over all seven species studied. The high confidence of the FReD structure predictions is reflected in the extreme value of the average pLDDT score (96 ± 7).
D Summary of 3 *D. melanogaster* RNAi lines crossed to full body GAL4 driver (tubulin) or neuron-specific (Nsyb) and the effects on viability.
E Number of flies analysed for the climbing assay in Fig 2D. Box plots depict data mean and upper and lower quartile; whiskers are the minimum and maximum number

A

```
CLUSTAL O(1.2.4) multiple sequence alignment

FReD_DROME      TATRQLPSSCSYSFLS---NHGILKVQLTPESESFYVSCD-----EDWTVILSRTSDDVN    52
FReD_HUMAN      CATGSRPRDCLDVLLSGQQDDGVYSVFPTHYPAGFQVYCDMRTDGGGWTVFQRREDGSVN    60
FReD_MOUSE      CANGSRPRDCLDVLLSGQQDDGVYSIFPTHYPAGFQVYCDMRTDGGGWTVFQRREDGSVN    60
                 *. . * .*   :**   :.*: .:  *    .* * **      .***:  * ...**

FReD_DROME      FERGWLDYRDGFGNLAGDFFIGLNKLHALTSSALHELRIVMEDFSGNVAYAGYSLFAI--    110
FReD_HUMAN      FFRGWDAYRDGFGRLTGEHWLGLKRIHALTTQAAYELHVDLEDFENGTAYARYGSFGVGL    120
FReD_MOUSE      FFRGWEAYREGFGKLTGEHWLGLKRIHALTTQAAYELHVDLEDFDNGTAYAHYGSFGVGL    120
                * ***  **:***.*:*:.::**:::****:.* :**:: :***....*** *. *.:

FReD_DROME      ---GSEKELYPLVLLGKFQDNLTPSAGDSLSYHAGAKFSTVDQDNDNCLECNCALRHKGA    167
FReD_HUMAN      FSVDPEEDGYPLTVA-----DYSGTAGDSLLKHSGMRFTTKDRDSDH-SENNCAAFYRGA    174
FReD_MOUSE      FSVDPEEDGYPLTVA-----DYSGTAGDSLLKHSGMRFTTKDRDSDH-SENNCAAFYRGA    174
                   . *:: ***.:      : : :*****  *:* :*:* *:*.*:  * ***  ::**

FReD_DROME      GWFNNCAKSNLFGEYTTQNQP-GETGIWWDTFSGQ-NSLKRVRWMIRPIS      215
FReD_HUMAN      WWYRNCHTSNLNGQYLRGAHASYADGVEWSSWTGWQYSLKFSEMKIRPVR      224
FReD_MOUSE      WWYRNCHTSNLNGQYLRGAHASYADGVEWSSWTGWQYSLKFSEMKIRPVR      224
                 *:.** .*** *:*    :     *: *.:::*   ***  .  ***:
```

	DROME	HUMAN	MOUSE
DROME	100	41.63	40.19
HUMAN	41.63	100	96.88
MOUSE	40.19	96.88	100

B

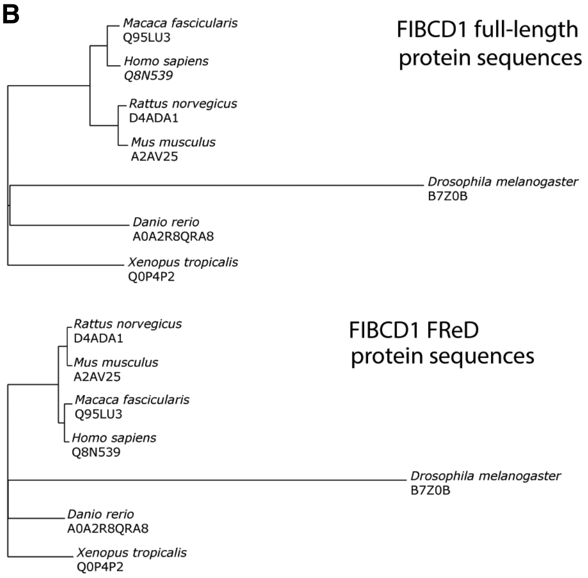

C

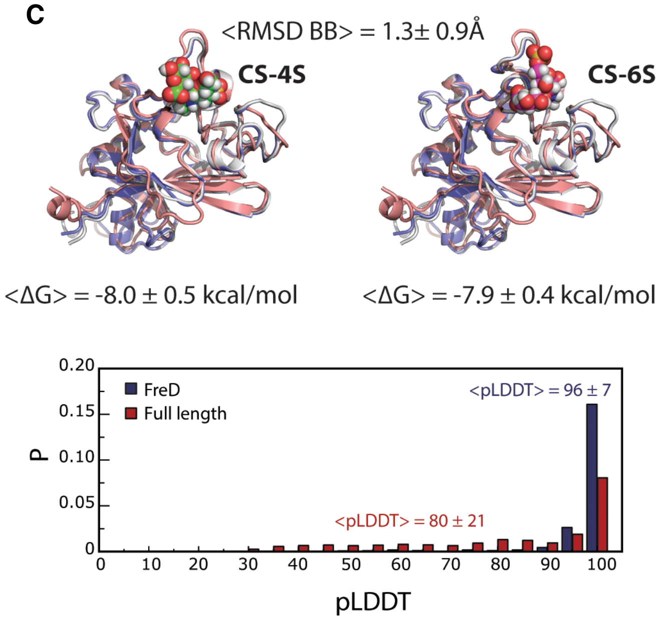

D

RNAi Line	Tub>Gal4 (Fullbody KD)	Nsyb>Gal4 (Neuronal KD)
UAS>CG10359 VDRC[102670] (Line #1)	Viable	Viable*
UAS>CG10359 VDRC[4128] (Line #2)	Semi-lethal	Viable*
UAS>CG10359 IR[BL63703] (Line #3)	Lethal	Viable*

* results in smaller larvae and skewed mendelian ratio

E

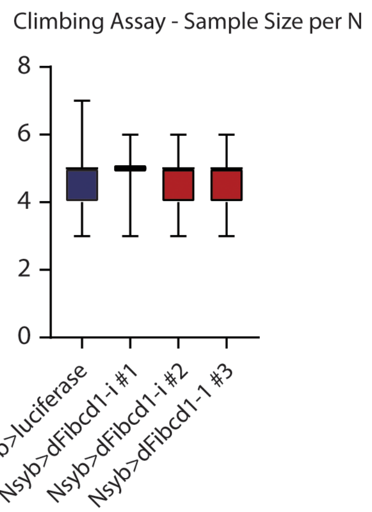

Figure EV2.

© 2022 The Authors

Figure EV3. Phenotyping FIBCD1-deficient mice.

A RT–qPCR of *Fibcd1* WT and KO adult mouse hippocampi (*n* = 4) using primer pairs binding to indicated exons (see Fig EV1A).

B–D Body weight (B) total brain volume (C) and brain volumes of denoted regions (D) of the indicated genotypes as assessed by MRI volumetric analysis. *n*(*Fibcd1* WT) = 6; *n*(*Fibcd1* KO) = 7. Insets are 3D representative MRI renditions of control (left) and *Fibcd1* KO (right) adult male brains with analysed brain regions pseudo-coloured.

E Percentage of time mice spent in the centre, open and closed arms of the elevated plus maze (EPM). *n*(*Fibcd1* WT) = 18; *n*(*Fibcd1* KO) = 26.

F The distance (left) and velocity (right) travelled by the mice in the open and closed arm of the EPM. *n*(*Fibcd1* WT) = 18; *n*(*Fibcd1* KO) = 26.

G Average latency of eight trials for each cohort to reach the target platform during the five training days in the Morris water maze (MWM). *n*(*Fibcd1* WT) = 9; *n* (*Fibcd1*KO) = 15.

H Percentage of time spent in the target quadrant during the short- and long-term probe trial in the MWM. *n*(*Fibcd1* WT) = 9; *n*(*Fibcd1* KO) = 15.

I Left, latency to reach the visible platform (VP) in the MWM; and right, distance travelled during the short- and long-term probe trials in the MWM. *n*(*Fibcd1* WT) = 6; *n*(*Fibcd1* KO) = 7).

J Left, latency of the animal to fall off the beam in the non-rotating Rotarod performance test; right, latency to fall of the 4 rpm rotating beam of two independent trials (*n* = 5).

K Average latency of four consecutive trials to fall off the 4–40 rpm rotating beam in a Rotarod performance test (*n* = 5).

L Acute pain responses to hotplate, intraplantar capsaicin injections or acetone drop quantified as time to first response or time spent licking or biting the injected paw, respectively, and reaction score to electrical foot shock. *n*(*Fibcd1* WT) = 9; *n*(*Fibcd1* KO) = 10.

Data information: For panels (A–L), each data point represents an individual mouse. Data are shown as mean values, and error bars represent SEM. *P* values were calculated using unpaired Student's *t*-test. ****$P < 0.0001$.

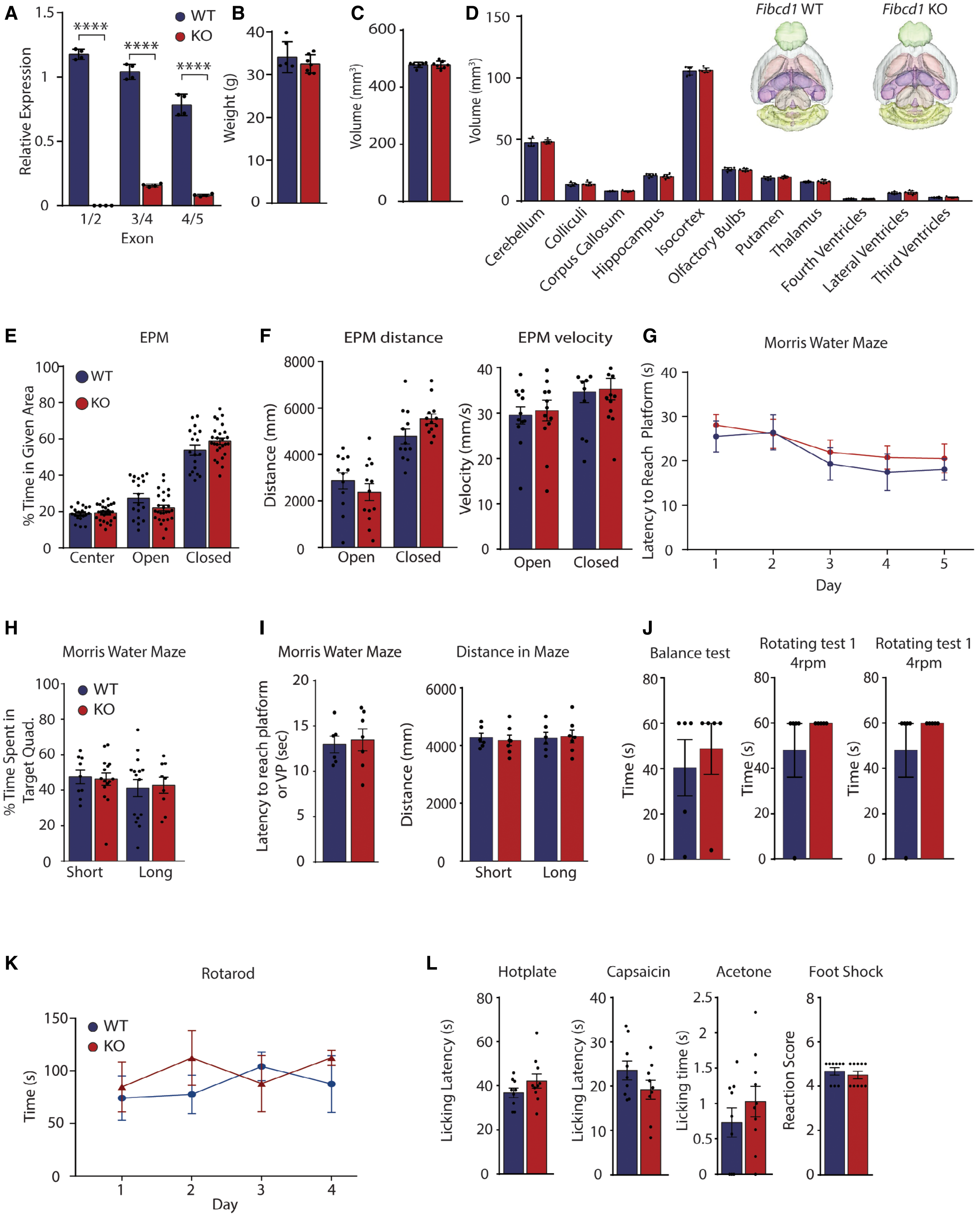

Figure EV3.

© 2022 The Authors

Figure EV4. Docking site of CS-6S in FIBCD1 FReD and validation of mFIBCD1 overexpressing N2a cell lines.

A Top binding pose for *in silico* docking of CS-6S to FIBCD1 FReD (PDB 4M7F).

B Schematic representation of FIBCD1 domains, IC, intracellular domain (red); TM, transmembrane domain (pink); CC, coiled-coil domain (dark blue); FReD (light blue); and location of V5-tag (grey) in full-length mFIBCD1 cDNA and truncated mFIBCD1 lacking the FReD (FIBCD1 ΔFReD).

C, D Relative mRNA expression levels of *Fibcd1* in the N2a cells overexpressing full-length (*Fibcd1*) or truncated *FIBCD1* (*Fibcd1 ΔFReD*) and adult mouse WT brain for comparison, analysed by RT–qPCR ($n = 4$) using primers binding to exons 1 and 2 before the FReD domain (C) or to exons 4 and 5 spanning the sequence encoding part of the FReD (D). Note the complete absence of endogenous *Fibcd1* expression in the "empty vector" (red bar) control and the complete absence of expression when using primers complementary to exon 4/5 (D), which span the FReD (see Fig EV1A) in the *Fibcd1* ΔFReD construct (C, pink bar), validating the generated cell lines. *Gapdh* was used as housekeeping control, and values obtained from a control brain sample were set to 1.

E Validation of transgenic N2a cell line at the protein level by immunoprecipitation with anti-V5 antibody as bait. Input (left) and V5-immunoprecipitated (right) lysates from N2a cells expressing V5-tagged full-length mFIBCD1 (mFIBCD1-V5, predicted size of 55 kDa), V5-tagged mFIBCD1 lacking the FReD (V5-FIBCD1 ΔFReD, predicted size of 28 kDa) or the empty vector as negative control. Protein marker sizes are indicated.

F Number of HEK293T cells per field during the CS-4S internalisation experiments, linked to Fig 4F ($n = 5$).

G Representative immunofluorescent images of HEK293T-FIBCD1 cells stained with FITC-CS-4S (green), CellMask (red) and Hoechst (blue); bottom and right panels are orthogonal views. Scale bar = 25 µm.

Data information: For panels (C, D and F), each data point represents an individual cell preparation and data are plotted as mean with error bars representing SD.

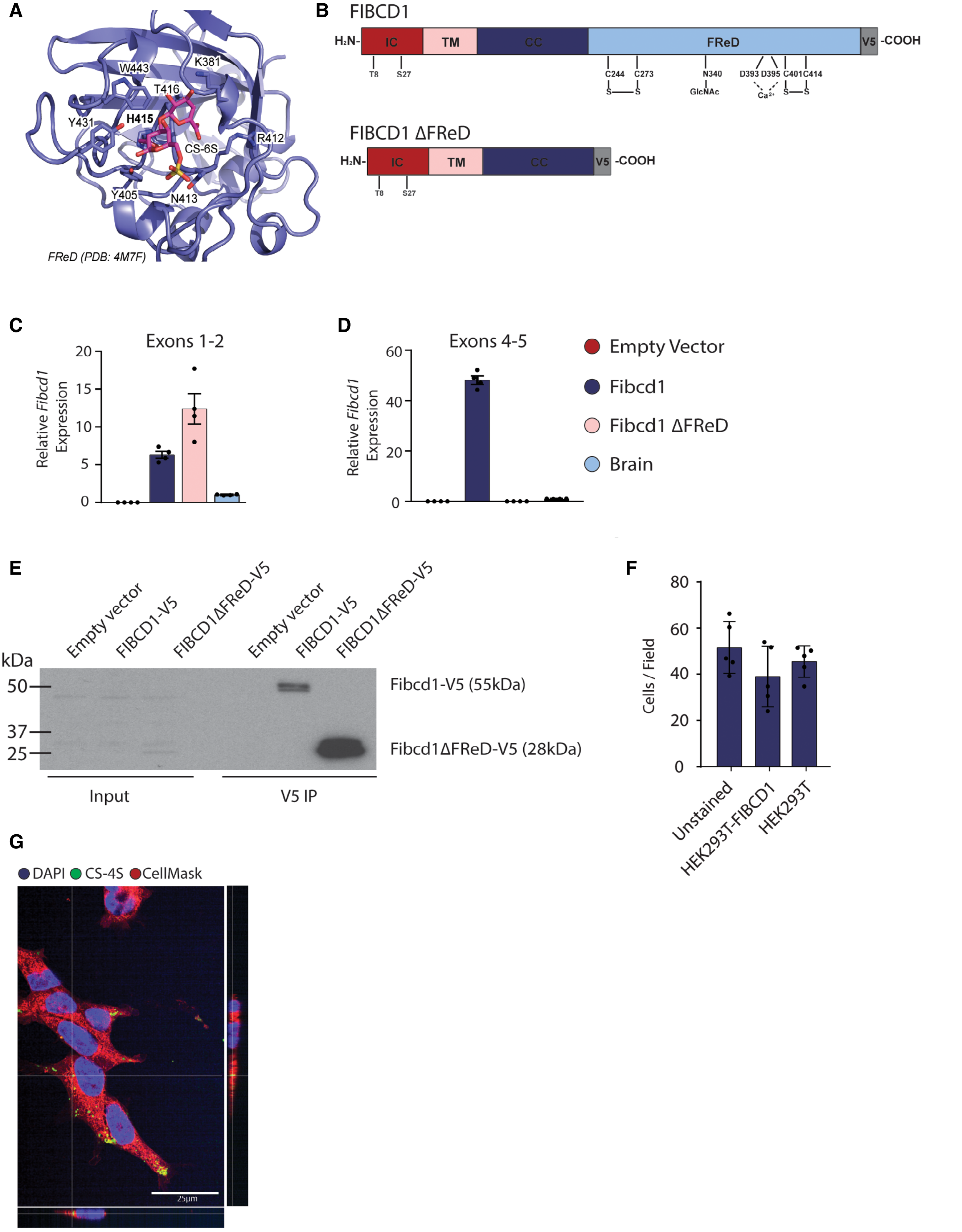

Figure EV4.

 © 2022 The Authors

Figure EV5. Additional human FIBCD1 data.

A Time course of the backbone root-mean-square deviation (RMSD) from the starting configuration for WT (blue), R406C (pink) and P456L (red) MD simulations.
B Missense, frameshift, splice region and stop gain variants extrapolated from the gnomAD database present in the population, colour code is indicated in the figure. Each dot represents one distinct variant, amino acid position and CADD score indicated on *x*- and *y*-axis. Denoted with arrows are the variants discussed in the present study.
C Validation of FLAG-FIBCD1 expression in stably expressing HEK293T cells by immunofluorescence. Note the absence of signal in untransduced cells and cells expressing truncated (W6*) FIBCD1. Shown are DAPI (blue), anti-FLAG (green) and merge. Scale bar = 50 μm. Representative of two independent experiments.

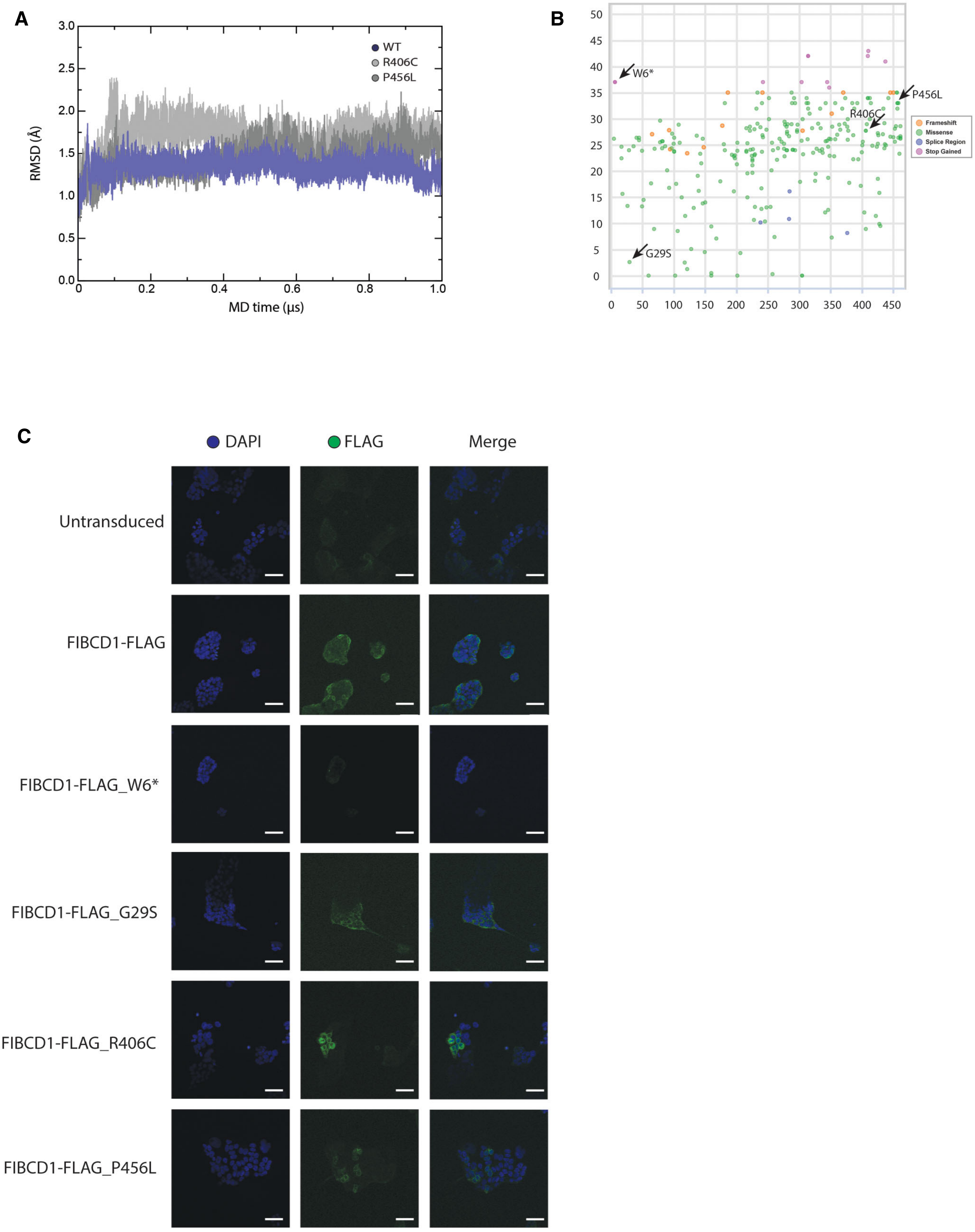

Figure EV5.

© 2022 The Authors
