# Supplementary material for: FIBCD1 is an endocytic GAG receptor associated with a novel neurodevelopmental disorder
Source: EMBO Mol Med. 2022 Aug 2;14(9):e15829. doi: 10.15252/emmm.202215829 (PMC9449597; doi:10.15252/emmm.202215829)
Supplement: Supplementary file 3 [file EMMM-14-e15829-s002.pdf]

EMBO Molecular Medicine

FIBCD1 is an endocytic GAG receptor associated with a novel neurodevelopmental disorder

Christopher Fell, Astrid Hagelkruys, Ana Cicvaric, Marion Horrer, Lucy Liu, Joshua Li, Johannes Stadlmann, Anton Polyansky, Stefan Mereiter, Miguel Tejada, Tomislav Kokotovic, Venkat Swaroop Achuta, Angelica Scaramuzza, Kimberly Twyman, Michelle Morrow, Jane Juusola, Huifang Yan, Jingmin Wang, Margit Burmeister, Biswa Choudhury, Thomas Andersen, Gerald Wirnsberger, Uffe Holmskov, Norbert Perrimon, Bojan Zagrovic, Francisco Monje, Jesper Moeller, Josef Penninger, and Vanja Nagy
DOI: 10.15252/emmm.202215829

Corresponding author: Vanja Nagy (vanja.nagy@rud.lbg.ac.at) Josef M.Penninger (josef.penninger@ubc.ca)

Review Timeline:

Submission Date:	4th Feb 22
Editorial Decision:	2nd Mar 22
Revision Received:	31st May 22
Editorial Decision:	27th Jun 22
Revision Received:	5th Jul 22
Accepted:	7th Jul 22

Editor: Jingyi Hou

Transaction Report:

(Note: With the exception of the correction of typographical or spelling errors that could be a source of ambiguity, letters and reports are not edited. Depending on transfer agreements, referee reports obtained elsewhere may or may not be included in this compilation. Referee reports are anonymous unless the Referee chooses to sign their reports.)

1st Editorial Decision **2nd Mar 2022**

3rd Mar 2022

Dear Dr. Nagy,

Thank you again for submitting your work to EMBO Molecular Medicine. We have now heard back from the three referees who agreed to evaluate your manuscript. As you will see from the reports below, the referees acknowledge the novelty and potential interest of the study. Still, they raise a series of concerns, which we would ask you to address in a revision of the manuscript.

The referees' recommendations are relatively straightforward, so there is no need to reiterate their comments. In particular, Referee #1 mentioned that the presentation of the manuscript needs to be improved to make the study more accessible to the general audience of EMBO Molecular Medicine. Further, in light of Referee #3's concern, additional analyses are required to better support the proposed endocytotic function for FIBCD1.

We would welcome the submission of a revised version within three months for further consideration. Please note that EMBO Molecular Medicine strongly supports a single round of revision. As acceptance or rejection of the manuscript will depend on another round of review, your responses should be as complete as possible.

EMBO Molecular Medicine has a "scooping protection" policy, whereby similar findings that are published by others during review or revision are not a criterion for rejection. Should you decide to submit a revised version, I do ask that you get in touch after three months if you have not completed it to update us on the status.

We are aware that many laboratories cannot function at full efficiency during the current COVID-19/SARS-CoV-2 pandemic and have therefore extended our "scooping protection policy" to cover the period required for a full revision to address the experimental issues. Please let me know should you need additional time, and also if you see a paper with related content published elsewhere.

Please read below for important editorial formatting and consult our author's guidelines for proper formatting of your revised article for EMBO Molecular Medicine.

I look forward to receiving your revised manuscript.

Use this link to login to the manuscript system and submit your revision: Link Not Available

Kind regards,
Jingyi

Jingyi Hou
Editor
EMBO Molecular Medicine

*****

***** Reviewer's comments *****

Referee #1 (Comments on Novelty/Model System for Author):

The data are novel and are embedded in a discovery from patient data. The data are extensive, make use of flies and mice, and the findings are exciting. These strengths are undermined by the presentation style--which needs some aggressive editing--but can be easily remedied.

Referee #1 (Remarks for Author):

This study identifies biallelic variants of FIBCD1 in patients suffering from neurodevelopmental disorders and having several shared phenotypes. Using multiple approaches across species, the investigators establish the role of FIBCD1 as a neuronal receptor for sulfated chondroitin sulfate glycosaminoglycans that modulates the extracellular matrix. This alone is exciting as several lines of data have associated CSPG with neurodevelopmental disorders, but mechanistic pathways are poorly understand. The data are mostly very strong and comprehensive and the findings are novel, providing insights into FIBCD1 structure, ligand binding properties and function within fly and mouse brain. However, impact is reduced due to a presentation style that is repetitive, not well synthesized, and dense with acronyms. Additionally the paper would benefit from a more in-depth discussion of the results and potential implications in relation to the current state of the field. There were only modest concerns with the science.

1. The manuscript was hard to read. The introduction would benefit from an increase in focus and an improved narrative that better outlines a story. The detailed presentation of the results in this section should be greatly abbreviated. The results section reads as a compilation of independent efforts, like entries in an encylopedia, and would benefit from a more uniform voice and some consolidation. The discussion is the best written section, but would be enhanced by better synthesis across results and with the literature.
2. Statistical approaches are incompletedly presented for most of the data. N's, replicates, means and error are all provided. But additionally, all of the data should cite the statistical test used, p values for ANOVA (or mixed models for groups having different N (e.g. Fig 2F), and the posthoc test used.
3. Fig. 1B shows MRI scans of patients, highlighting changes in brain architecture caused by the mutant FIBCD1. It is recommended to use a better contrast image along with arrowheads to point to the regions of interest.
4. In Fig. 2A and B, bouton numbers were quantified and show the most significant change with RNAi construct#3; however, the representative image for RNAi construct #3 in Fig. 2A shows little to no difference in appearance compared to control. The authors should pick a better representative image for RNAi #3 that better reflects the quantified results.
5. In Fig. 2E, HET mutants were not tested on the Y-maze as they were for other tasks- is there a reason for excluding this genotype for the Y-maze behavioral analysis?
6. Additional detail is required for how microscopy data (e.g Fig. 2, 3F, 4A,B) were sampled (fields of view, cells, segmented areas, axon), quantified (intensity, area, number), and reported (normalized or not). Within biological replicates, how many cells? Regions? Coverslips? For lower numbers of biological replicates, what was the range? How were "puncta" or cell clumps defined? (Fig 4B). What image analysis program (if any) and if by user, was this user blinded to treatment conditions? Some information is reported in legends, but it is incomplete and inconsistent between datasets.

7. In Fig. 4, the authors refer to each N as a biological replicate, is this referencing number of preps, or number of cells, or number of coverslips?

Referee #2 (Remarks for Author):

This study by Fell et al., found that mutations of FIBCD1 2 patients with neurodevelopmental disorders. The authors then characterised the spatiotemporal expression of FIBCD1 in human and mice samples. For functional characterization, they employed behavioural studies in flies knocked-down for a putative Fibcd1 orthologue, CG10359, and knock-out mice. Regarding the signaling mechanisms, they showed FIBCD1 is a GAG receptor and mediates GAG signalling in neurons. Importantly, they showed that patient FIBCD1 variants disrupt FIBCD1-CS-4S association, therefore are loss-of-function. Interesting, the synaptic deficits in KO hippocampal slices can be rescued by ChABC, an enzyme that digests CSPGs. In general, this study is interesting and provides a mechanism for a novel gene associated to neurodevelopmental disorder. However, one issue is that only parts of the patient phenotypes are observed in knockout mice. As the authors pointed out, some other mutations of other genes are found in patients. That may indicates that FIBCD1 mutation can only contribute to part of the clinical observations. I think the authors need to point that out more clearly in the discussion. Currently, it's more like a (long) description of the clinical observation.

Major comments:
1. The authors try to use 'neurodevelopmental disorder' as an umbrella term to describe the clinical features of both patient 1 and patient 2. However, as shown in table 1 the clinical presentation of patient 1 (P1) and patient 2 (P2) are quite different. P1 has normal sitting and walking while P2 has delay. P1 has (mild) borderline delayed cognition, and ADHD combined type. However, P2 at 3 y.o. showed more severe delayed social and cognitive abilities. P1 has motor deficits but P2 does not. The authors avoided these differences and tried to focus on the similarity between 2 patients. The authors should summarize these differences and discuss it in a more balanced manner. It's understandable that two patients showed difference clinical findings and P2 may contains other potential pathological mutations.

2. Neuronal knockdown of dFibcd1 in Drosophila showed abnormal morphology and reduced neuronal branching. Are there any morphological deficits in the hippocampal neurons of FIBCD1 KO mice? Structural deficits of dendritic spines or dendritic arborization?

3. P1 has fine motor deficits. Does the KO mice have fine motor deficit?

3. Why the WT slices treated with ChABC exhibit reduced long-term potentiation (LTP) while KO slices treated with ChABC exhibit increased LTP? The author can include an explanation in the text although they said it's consistent with previous literature.

4. The authors performed a series of hippocampal dependent behaviour tasks. However, only some of the tasks showed significant difference between WT and KO mice such as Y-maze. There is no difference in MWZ. While the expression of FIBCD1 is mainly in the hippocampus, can the authors provide more discussion why the behavioural deficits in mice is actually quite mild as compared to human.

5. Electrophysiological expt, as shown in Figure S7, the Input/Output curve has no difference. So the major deficit is on LTP (synaptic plasticity). Can the authors identified some DEG in RNAseq to explain the synaptic plasticity deficit ? The current DEG description mainly focuses on ECM and CSPG signaling.

Minor:

The method section for "Acute hippocampal slice preparation and electrophysiological recordings " should provide a brief summary, instead of merely citing previously published papers.

In Supple. Fig 3., the fonts in the x-axis look a bit distorted.

Referee #3 (Remarks for Author):

The manuscript by Fell et al. investigates the consequences of point mutations in FIBCD1 that they identified in two patients with

idiopathic neurodevelopmental disorders. Analysis of human and mouse tissue shows high FIBCD1 expression in the glands, testis, and CNS, more specifically in hypothalamus and hippocampus. Expression knockdown in fly nervous system results in reduced locomotion, while a mouse knock-out (KO) model result in impaired fear-associated learning. These mice show normal anxiety-like behavior, spatial learning, and response to acute pain. The authors convincingly show that FIBCD1 preferentially binds to chondroitin sulfate (CS) with 4-O sulfation (CS-4S), and that this binding is disrupted by mutations corresponding to the variants identified in the two patients. Primary hippocampal cultures derived from wild type (WT) or KO mice have different growth patterns when grown on CS proteoglycan (CSPG) coating. RNA sequencing of these cultured neurons ({plus minus} KO, {plus minus} CSPG coating) suggest that a consequence of FIBCD1 activity is the regulation of genes related to integrins and the extracellular matrix. Finally, they show that hippocampal slices from KO mice have impaired synaptic plasticity that is rescued by digestion of CSPGs. While a major claim made in this study (it's in the title) is that FIBCD1 mediates CS endocytosis, the endocytic activity of FIBCD1 is not convincingly shown. This major concern and other concerns need to be addressed.

Major concerns
1. The evidence for endocytotic function for FIBCD1 is provided by FACS experiments of N2a cell uptake of CS-4S-fitc and by immunocytochemistry (IHC) of HEK cell uptake of CS-4S-fitc. From the FACS experiments, it is not clear whether CS-4S-fitc is associated with cell surface or internalized. Control experiments are needed whereby endocytosis is inhibited/blocked and/or extracellular fitc is quenched. From the IHC experiments, it is very unclear whether CS-4S-fitc is endocytosed. The images (Fig 3F) show clumps (aggregates?) outside the cell, and similar clumps are seen within (on?) the cell. It is not clear what is being counted (and the graph in Fig 3F lacks units). Confocal microscopy or deconvolution is needed to show that the puncta are within the cell and have sizes consistent with endocytotic vesicles. Immunostaining for V5 (FIBCD1 WT and mutant constructs shown in Fig 3E and S4B) or FLAG (Fig 3H) and endocytotic vesicle markers in N2a (or HEK) cells should be shown.

2. The authors claim to show "evolutionar(il)y conserved" function for FIBCD1 based on behavior phenotypes in human, fly, and mouse. To support this claim, authors should perform phylogenetic analyses and perform similar molecular function experiments across species (e.g. CS levels in fly loss-of-function, molecular dynamics of docking CS-FIBCD from different species, etc). Apart from the phenotypic outcomes, no evidence is provided for conserved molecular function.

3. The normalization of LTP by ChABC of slices from Fibcd1 KO mice is an intriguing finding, and more so given that ChABC has the inverse effect on WT slices. However, the statement on lines 578-9 that "complete rescue of remodeling deficiencies by ChABC treatment supports our hypothesis that GAG accumulation lies at the centre" of the pathology, is misleading. While paired-pulse facilitation is rescued by ChABC on KO slices, the change in LTP is not in line with the effects of ChABC on WT slices. The rescue of LTP is discussed (lines 665-671) but is not put into the context of the effects observed on WT slices. Clearly the picture is more complex than simply "pathological accumulation", and further experiments are need for the issue to be reconciled. Apart from editorial changes, it may be of interest to treat the slices with various CS compounds. In vivo hippocampal infusion of ChABC and CS compounds to affect WT behavior or rescue KO behavior could also be used to address this issue of "accumulation".

Minor concerns
1. Statistics are missing in Figures 1G and 1F.

2. The peaks in Figure 3A should be quantified by providing ratios to an internal standard. What is the peak at ~19 minutes?

3. In Figures 4A and 4B, there are discrepancies in the N given in the legend and the number of points in the graph. The graph in Fig 4B lacks units.

4. Statistics are missing in Figure S1B-D.

Dear Dr. Hou,

We are grateful for the detailed and constructive suggestions from you as well as the reviewers for the improvement of our manuscript, no. EMM-2022-15829.

The improved versions (with and without tracked changes) of the manuscript have been uploaded to the submission forum. Please note that the responses below refer to the line numbers in the untracked version for simplicity. Below are the point-to-point responses to all suggestions, issues and criticisms.

EDITOR COMMENTS:

1- Referee #1 mentioned that the presentation of the manuscript needs to be improved to make the study more accessible to the general audience of EMBO Molecular Medicine.

Thank you for this suggestion. We have revised the manuscript in its entirety and have enlisted the help of several colleagues for text editing. Please see below for a more detailed description of the editing and stylistic manuscript changes.

2- Further, in light of Referee #3's concern, additional analyses are required to better support the proposed endocytotic function for FIBCD1.

We are in agreement with the reviewer and editor that additional experimental evidence of FIBCD1 endocytic function is necessary to support the proposed model of molecular pathology resulting from FIBCD1-deficiency. The first evidence of endocytic FIBCD1 function was published in 2009 (Schlosser et al, 2009). In the original submission, we have provided evidence in Fig. 3 A-F (now, Fig 4 A-F) of chondroitin sulfate dysregulation in mouse brain in the absence of FIBCD1, as well as in silico, cell-free and cell-based binding assays to demonstrate FIBCD1 binding to chondroitin sulfates.

To further substantiate the claims that FIBDC1 is facilitating endocytosis of its ECM ligand we performed the following experiments:

1. *Inhibition of endocytosis in a cellular assay of CS-4S binding visualized with immunofluorescence using 2 different compounds: PitStop2 (cell permeable clathrin inhibitor) and Dynole 34-2 (dynamin 1 and 2 inhibitor) (Fig. 4G). Briefly, we found treatment with either compound reduced intracellular CS-4S punctae in FIBCD1-expressing cells compared to their inert negative control, indicating that the puncta were internalized through endocytosis facilitated by FIBCD1.*
2. *Higher magnification and orthogonal slice analysis of cells expressing WT FIBCD1 revealed large, intracellular positive CS-4S inclusions demonstrating that FIBCD1 ligand CS-4S is internalized within the cell and not 'just stuck' on the cellular membrane (Fig. EV4G).*

3. *Co-localisation of FIBCD1 with markers of endocytic vesicles: namely, we demonstrate that FITC-labeled CS-4S colocalized with mCherry-labeled wild type FIBCD1 as well as with LysoTracker, a fluorescent dye used to label acidic organelles such as lysosomes concluding that FIBCD1 facilitates internalization of CS-4S into lysosomal vesicles (Fig. 4H). Detailed technical description and location of experiments and accompanying text in the revised version of the manuscript can be found in the point-to-point response to Reviewer #3.*

REFEREE COMMENTS:

REFEREE 1:

We are grateful to the referee for the overall positive review of our work and for the constructive and helpful suggestions. Please note that line numbers refer to the unmarked version of the revised manuscript. All suggested points and issues listed below are bolded and in chronological order with point-to-point comments in italics.

1-The data are novel and are embedded in a discovery from patient data. The data are extensive, make use of flies and mice, and the findings are exciting. These strengths are undermined by the presentation style--which needs some aggressive editing--but can be easily remedied.

…presentation style that is repetitive, not well synthesized, and dense with acronyms. Additionally the paper would benefit from a more in-depth discussion of the results and potential implications in relation to the current state of the field.

…the manuscript was hard to read. The introduction would benefit from an increase in focus and an improved narrative that better outlines a story. The detailed presentation of the results in this section should be greatly abbreviated. The results section reads as a compilation of independent efforts, like entries in an encylopedia, and would benefit from a more uniform voice and some consolidation. The discussion is the best written section, but would be enhanced by better synthesis across results and with the literature.

We thank the reviewer for the positive assessment of our manuscript and the constructive suggestions for its improvement. We agree the original manuscript required substantial revision and editing which were completed bearing in mind all the detailed editorial suggestions listed above. We have additionally enlisted the help of several colleagues for editing and stylistic advice. The major changes that were made take into account all the above mentioned suggestions, including:

1. *Reduction of acronyms, particularly in the introduction, which was also substantially reduced to focus only on the topic at hand.*
2. *Re-ordering of the results section for a more natural flow (figure 5 moved to figure 3).*
3. *More in-depth discussion embedded in the current state of the field was included.*
4. *Result section was edited to continuously reference and stress the point that we explain the patient symptoms via the animal models, and therefore the results are more intertwined as opposed presented as a list of separate series of experiments.*
5. *Discussion section was edited as per reviewer's suggestion.*

2-Statistical approaches are incompletedly presented for most of the data. N's, replicates, means and error are all provided. But additionally, all of the data should cite the statistical test used, p values for ANOVA (or mixed models for groups having different N (e.g. Fig 2F), and the posthoc test used.

We are grateful to the reviewer for noting the inconsistency in the data presentation. We have now edited the manuscript to provide a more detailed statistical representation: in addition to the statistical analysis statements in the Materials and Methods section where relevant , for each figure panel the legend now contains a clear definition of technical and biological replicates, each statistical analysis was noted and p values clearly defined.

3-Fig. 1B shows MRI scans of patients, highlighting changes in brain architecture caused by the mutant FIBCD1. It is recommended to use a better contrast image along with arrowheads to point to the regions of interest.

We thank the reviewer for pointing out the lack of clarity in the MRI images as this is a critical point for the clinical readership. The contrast was improved, and asterisks were added in the images and defined in the legend to improve the overall presentation of the structural brain anomalies associated with the disease. Please note the changes in Fig. 1B.

4-In Fig. 2A and B, bouton numbers were quantified and show the most significant change with RNAi construct#3; however, the representative image for RNAi construct #3 in Fig. 2A shows little to no difference in appearance compared to control. The authors should pick a better representative image for RNAi #3 that better reflects the quantified results.

We are very grateful for the reviewer noting the discrepancy of the quantification with the chosen representative images. In the original manuscript the panels for RNAi #2 and #3 were somehow swapped, and this mistake is now rectified.

5-In Fig. 2E, HET mutants were not tested on the Y-maze as they were for other tasks- is there a reason for excluding this genotype for the Y-maze behavioral analysis?

We are grateful to the reviewer for noting this lack of consistency in the behavioural data presentation pertaining to heterozygous animal cohort. Inhibitory avoidance was the very first behavioural experiment that was performed, and we had used all the animals available, which is why heterozygotes were included. Following identification of the patients and detailed description of the inheritance pattern that demonstrated that carriers (heterozygous family members) did not exhibit any neurological symptoms, in an attempt to adhere to the 3R policy of our institute (replace, reduce and refine) we omitted the heterozygotes from the other behavioural tests. To address the reviewer concern for consistency we have omitted the heterozygous data from the Inhibitory avoidance task.

6-Additional detail is required for how microscopy data (e.g Fig. 2, 3F, 4A, B) were sampled (fields of view, cells, segmented areas, axon), quantified (intensity, area, number), and reported (normalized or not). Within biological replicates, how many cells? Regions? Coverslips? For lower numbers of biological replicates, what was the range? How were "puncta" or cell clumps defined? (Fig 4B). What image analysis program (if any) and if by user, was this user blinded to treatment conditions? Some information is reported in legends, but it is incomplete and inconsistent between datasets.

We agree with the reviewer that additional details are needed to define the robustness of our immunocytochemical datasets and are now included in the Materials and methods section (pg. 15, lines 303-309 and pg. 16-17, lines 320-335) and/or the accompanying figure legends.

Specifically, for the HEK cells experiments (now in Fig. 4F-G), approximately 50 fields of view were acquired per well (i.e., per condition, per replicate) in an automated fashion at 63x

magnification. The analysis was done by an automated imaging pipeline in Perkin Elmer Harmony software (see Unpublished Figure 1 below). The pipeline first used the 'Find nuclei' module in the blue channel (Hoechst) to identify and segment nuclei. Then, the 'Find cytoplasm' module was used in the red channel (CellMask, staining cell membranes) to identify the cell boundaries and thus segment each cell. In the green channel, the images were 'masked,' by setting all pixels outside of the cell boundaries to black to reduce noise or false-positives coming from precipitate. Puncta were then identified using the 'Find spots' module in the masked green images which were then quantified. There were approximately 40-60 cells per field of view (Fig. EV4F). These details have now been added to the Materials and methods section (pg. 16, lines 326-335).

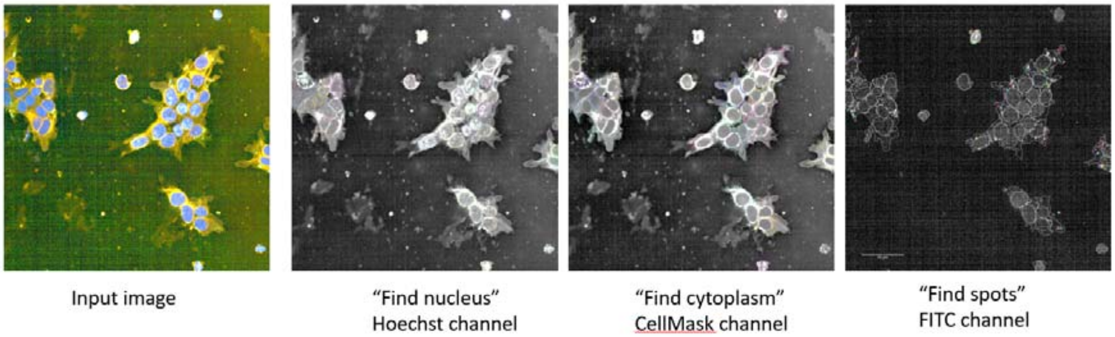

Unpublished Figure 1: Example of nuclei, cell boundary and CS-4S segmentation by our image analysis pipeline in Harmony. Note the coloured rings in each segment showing what the algorithm has segmented at each step.

For the primary neuron experiments (now Fig 5A-B), approximately 10 fields of view were acquired semi-randomly by an experimenter. Images were analysed manually by an experimenter blinded to condition (e.g. untreated vs +CSPG) and genotype of the cells. All cells in each of field of view were counted and defined as 'clumped' or 'not clumped.' The experimenter would define cells as 'clumped' if there were approximately 10 or more MAP2+ somas in direct contact or very close proximity to each other. The data in Fig. 5A is normalised to the untreated condition; for Fig. 5B the number of clumped neurons are expressed as a % of all neurons counted in each image. These details have been added in Materials and methods section (pg.16, lines 304-312).

7-In Fig. 4, the authors refer to each N as a biological replicate, is this referencing number of preps, or number of cells, or number of coverslips?

As per the reviewer suggestion, all legends were edited to ensure that the N numbers are clearly defined. In the case of Fig. 4 (now, Fig. 5) n is referring to the number of independent cell culture preparations.

REFEREE #2:

We are very grateful to the referee for the overall positive assessment of our manuscript and the constructive and helpful suggestions. Please note that line numbers refer to the unmarked version of the manuscript. All reviewer points and issues listed below are bolded and in chronological order with point-to-point comments in italics.

However, one issue is that only parts of the patient phenotypes are observed in knockout mice. As the authors pointed out, some other mutations of other genes are found in patients. That may indicates that FIBCD1 mutation can only contribute to part of the clinical observations. I think the

authors need to point that out more clearly in the discussion. Currently, it's more like a (long) description of the clinical observation.

The reviewer's comment is absolutely correct – due to molecular species differences between flies, mice and men often phenotypes don't perfectly align. Additionally, during genetic analysis of undiagnosed patients often a small number of other potentially pathogenic rare variants come up (we have listed those for each patient in the Results section (pg. 21, lines 432-444 and pg. 22, lines 450-461) and Discussion section (pg. 34, lines 752-777)). Without a larger cohort of patients with FIBCD1 variants to compare the symptoms with, it is not possible to dismiss other variants (even if they are unlikely due to reasons such as association with a different rare disease and symptoms, lack of expression in relevant tissue or cell type etc…) We have revised the Discussion section to highlight this important point (pg. 35-36, lines 791-803).

1-The authors try to use 'neurodevelopmental disorder' as an umbrella term to describe the clinical features of both patient 1 and patient 2. However, as shown in table 1 the clinical presentation of patient 1 (P1) and patient 2 (P2) are quite different. P1 has normal sitting and walking while P2 has delay. P1 has (mild) borderline delayed cognition, and ADHD combined type. However, P2 at 3 y.o. showed more severe delayed social and cognitive abilities. P1 has motor deficits but P2 does not. The authors avoided these differences and tried to focus on the similarity between 2 patients. The authors should summarize these differences and discuss it in a more balanced manner. It's understandable that two patients showed difference clinical findings and P2 may contains other potential pathological mutations.

Indeed, as the reviewer rightfully points out, patient 1 and 2 have differences as well as overlapping symptoms. We have rewritten the patients' clinical comparisons to present a more balanced view of these differences, as suggested, please note pg. 35, lines 767-777. Additionally, the reviewer will note throughout the text we have consistently noted differences of phenotypes between genetically modified animal models as well as in comparison to each of the patients.

2-Neuronal knockdown of dFibcd1 in Drosophila showed abnormal morphology and reduced neuronal branching. Are there any morphological deficits in the hippocampal neurons of FIBCD1 KO mice? Structural deficits of dendritic spines or dendritic arborization?

We thank the reviewer for highlighting this important point and for the experimental suggestion. To address this point, we analysed both developing neurons in culture, as well as adult neurons in vivo. First, we did not note any morphological differences in primary neuronal cultures prepared from E18.5 Fibcd1 WT and KO mouse hippocampi (see Unpublished Figure 2). Briefly, we have cultured the cells for 2 days, fixed and stained them with MAP2, neuronal specific marker that reveals the neuronal structure in its entirety. We then analyzed the cells for dendritic number and dendritic branch number and found no difference between the two cohorts (*see Unpublished Figure 2).*

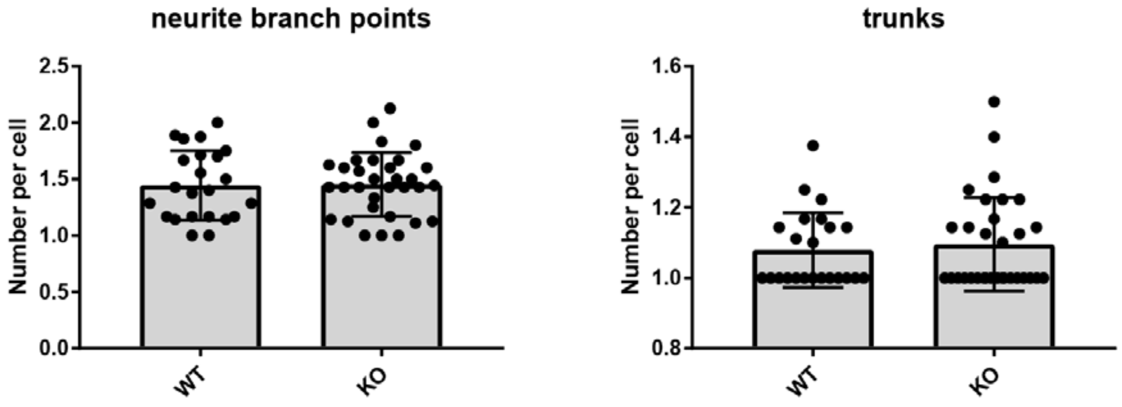

Unpublished Figure 2: Morphological parameters of *Fibcd1* WT and KO primary neurons at DIV2. Quantified here are the number of dendritic trunks from the cell soma (left) and the number of bifurcation points per dendrite per cell (right). n(*Fibcd1* WT) = 4; n(*Fibcd1* KO) = 2. Each point represents quantifications from one field of view.

To further investigate whether there are any morphological alterations of the hippocampal pyramidal cells in vivo in adults, we have performed a standard Golgi-Cox stain on adult Fibcd1 WT and KO mouse brains now included in Fig. 2E-K. We focused our analysis on the hippocampal pyramidal neurons, where FIBCD1 expression was highest. Following manual tracing in Neurolucida software (Fig. 2E) and analysis in Neurolucida Explorer, we determined a slight but significant increase in basal dendrite complexity (Fig. 2G-K) (pg. 24-25, lines 515-526), but no differences in number of basal dendrites, basal or apical total dendrite length or number of branches. Further manual analysis in ImageJ/Fiji software of proximal apical dendritic spines revealed no difference in spine density between Fibcd1 WT and KO neurons. The fact we did not detect any morphological deviations in neurons in culture as opposed to neurons in the adult brain can be explained by the difference in developmental stages of neuronal development and/or ECM composition of cell culture system compared to in vivo ECM. Nevertheless, together these data conclude that while neuronal morphological parameters are severely perturbed in the fly model of Fibcd1 deficiency, there is also slight, but significant difference in morphological parameters in the corresponding , mouse model, suggesting that Fibcd1 is important for neuronal development, likely by signalling cues from the ECM.

3-P1 has fine motor deficits. Does the KO mice have fine motor deficit?

We are grateful to the referee for the opportunity to highlight this important point. Indeed P1 has fine motor deficits, however, as is often the case with species (mouse and fly to human) comparisons, not all the phenotypes are recapitulated faithfully in the genetically modified animal models. Further, no motor deficits have been noted in P2. We remind the reviewer that the dFibcd1 knock-down flies exhibited significantly reduced climbing ability in a negative geotaxis assay as compared to controls (Fig. 2D). In this geotaxis assay, flies' innate instinct to climb is leveraged to determine whether dfibcd1 deficiency hinders general locomotion capacity. While no assay is currently available to determine "fine" motor deficits, the reduced climbing ability of the dFibcd1 flies strongly implicated a defect in locomotion, caused by the absence of functional dFibcd1 in line with the P1 motor deficits and P2 delayed sitting and walking milestones. Conversely, we did not note any overt motor deficits in the Fibcd1-KO mice as primarily concluded by the observation that the latency to reach the hidden platform in the Morris Water Maze (MWM) task that requires coordinated swimming movements was not different between the KO and control groups (Fig. EV3G). In fact, Fibcd1 KO animals were also able to increase their speed toward the escape platform as they learned the location exhibited by the reduced latency from the first day of trials to the 5th. Additionally, animals reacted similarly to the Hotplate assay, as well as capsaicin and acetone injections and the electric foot shock as their WT

counterparts (Fig. EV3L). Although these assays are directed towards determining pain thresholds and sensory function, ability to jump, lick, shake their feet and bite the affected area is required for the read-out of the task, and therefore can be used to ascertain the overall movement dysfunctions – which were not observed in any of the tests.

To confirm that animals are not affected motorically, we further re-analyzed already existing data and also performed additional tests. First, we examined the distance travelled and velocity of movement undertaken by Fibcd1 KO mice in the elevated plus maze (EPM) as compared to their WT littermates. While the percentage of time the animal spends in the open or closed arms is determinant of anxiety levels, the distance and speed travelled throughout the maze can be repurposed for determining the locomotion ability of the genetically manipulated cohort. We found no difference in either distance or speed between the two groups (Fig. EV3F). Next, to examine coordinated swimming abilities as a read-out of locomotion function in absence of FIBCD1 in more details, we re-examined the MWM data. We ascertained the latency to reach the visible platform at the start of MWM training and the distance animals swam in the short and long term probe trials at the end (Fig. EV3I) and found no differences in either, again suggesting Fibcd1-KO animals do not have deficits in coordinated swimming movements required for the task.

Finally, we subjected Fibcd1 WT and KO cohorts to an additional locomotion test, the Rotarod test, a gold standard assay for determining motor deficits in genetically modified mice. We performed several different trials to determine any balance, coordinated locomotion, or grip strength effects of FIBCD1 deficiency. We first measured the animals' ability to balance on the non-rotating beam, and found no significant difference in the two cohorts (Fig. EV3K). We next performed two trials on a rod that was rotating at a constant 4 rpm speed, and found no difference in the ability of the animals to balance on the rod between the two cohorts (Fig. EV3K). Finally, we set the animals to balance on an accelerating rod (4-40 rpm) for 5 minutes in 4 trials, and again found no significant difference between the cohorts (Fig. EV3J), suggesting no effect on balance, grip strength and coordinated locomotion ability due to absence of functional FIBCD1 (pg. 25-26, lines 527-555).

Taken together, these data suggest that in mice, unlike in flies, we did not detect any effects of motor skills in the absence of functional FIBCD1.

4-Why the WT slices treated with ChABC exhibit reduced long-term potentiation (LTP) while KO slices treated with ChABC exhibit increased LTP? The author can include an explanation in the text although they said it's consistent with previous literature.

We thank the reviewer for giving us the opportunity to discuss this very interesting experimental observation. The abrogation of LTP in WT slices by ChABC is consistent with previous literature (Bukalo et al, 2001) and is likely due to the disruption of molecules in the ECM that are supportive of synaptic structural remodelling and functional plasticity. On the other hand, others have utilised ChABC to rescue deficient hippocampal LTP in an Alzheimer's disease model, hypothesising that ChABC removed disease-associated accumulated CSPGs which were inhibiting LTP (Vegh et al, 2014). Additionally, ChABC injection was shown to promote spatial navigation (Saroja et al, 2014). However, the mechanism by which ChABC affects LTP or learning remains elusive. Functionality of ChABC treatment is generally defined by the loss of WFA positive perineuronal nets (PNNs) primarily consisting of CSPGs. However, it is likely that ChABC is also targeting monomeric CSPGs, not visualized by WFA, which are still to be identified. Indeed, aggrecan, versican, neurocan and other CSPGs are expressed in the brain and have distinct functions (Mencio et al, 2021). Further complexity confounding our understanding of CSPGs' role during synaptic activity is the lack of clarity of different sulfation patterns on the CSPGs glycosaminoglycan (GAG) chains (Rowlands et al, 2015). ChABC

treatment is likely releasing different GAGs in the perisynaptic area, which could be bioactive and could all have different signalling effects.
Nevertheless, we envision several possible explanations for our results, which are not necessarily mutually exclusive:

1. *The seemingly contradictory effect of ChABC on the Fibcd1 WT and KO slices suggests the mechanisms of FIBCD1 signalling and ChABC action to converge. This could be through chondroitin sulphates themselves, which are degraded (among other GAGs) by ChABC. We observed that FIBCD1 KO mice have increased levels of CS-4S content in hippocampal lysates (Fig 4B). The simplest explanation for our data is that the increased CS-4S in the KO hippocampi are inhibitory to LTP (as observed in KO+Pen slices) and ChABC treatment restored CS-4S levels to WT levels by "clearing away" the inhibitory GAG. However, this would mean that we serendipitously hit the right concentration and time of ChABC treatment in the KO slices to return to WT levels rather than overly degrade the GAGs, which would have presumably led to abrogated LTP as observed in WT+ChABC slices. Therefore, the right balance of different GAGs is required for proper synaptic function.*
2. *It is also possible that the products of ChABC treatment are stimulatory to LTP in certain conditions, though the mechanism of this is unclear (Vegh et al., 2014) (Saroja et al., 2014).*
3. *Another potential convergent mechanism is through integrin signalling. We and others (Graca et al, 2022) have shown that FIBCD1 regulates the expression of certain integrin subunits. ChABC has also been shown to stimulate β-1 integrin signalling in neurons (Senkov et al, 2014). It is possible that the contradictory effects of ChABC on WT and KO slices are due to different compositions of integrins on the neuronal surfaces caused by FIBCD1-deficiency.*

In summary, we stress that there are a number of factors wider than the scope of this manuscript to be elucidated before reaching an understanding of the mechanism/s that led to these results. It is clear, however, that our data open new lines of investigation in clarifying the role of ECM composition on synaptic plasticity and learning. Considering the number of possibilities and the extensive experiments that would be needed to substantiate them, we have not included this discussion in the text of the main manuscript.

5-The authors performed a series of hippocampal dependent behaviour tasks. However, only some of the tasks showed significant difference between WT and KO mice such as Y-maze. There is no difference in MWZ. While the expression of FIBCD1 is mainly in the hippocampus, can the authors provide more discussion why the behavioural deficits in mice is actually quite mild as compared to human.

We thank the reviewer for the opportunity to highlight this important observation. Indeed, as compared to the two patients we report, the Fibcd1 KO phenotypes were actually quite mild: spatial working memory tested by the Y-maze, fear conditioning tested by inhibitory avoidance and long-term potentiation of the Schaffer collateral pathway in acute hippocampal slices were all deficient. However, spatial learning as tested by Morris Water Maze, anxiety, sensory and motor learning and locomotion, including overall brain size and structure were all normal. Neurological symptoms are often difficult to model in mice, particularly those that affect complex behaviors included in ADHD and autism, as well as something as extreme as microcephaly. In addition, it is likely that FIBCD1 influences different molecular mechanism between species, best evident when comparing the severe locomotion deficiencies in flies and a lack of similar dysfunctions in mice. Additionally, FIBCD1 is a receptor for GAGs, which are highly diverse and have different functions in different species (Yamada et al, 2011). We have therefore included a more detailed discussion of species differences throughout the text, particularly pg. 36-37, lines 804-835.

6-Electrophysiological expt, as shown in Figure S7, the Input/Output curve has no difference. So the major deficit is on LTP (synaptic plasticity). Can the authors identified some DEG in RNAseq to explain the synaptic plasticity deficit ? The current DEG description mainly focuses on ECM and CSPG signaling.

We thank for the reviewer for the opportunity to discuss this in more detail. As the reviewer points out, RNA-seq data analysis identified dysregulation of a number of genes encoding ECM components as well as integrin subunits. Both the ECM as well as Integrins have long been known to be intimately linked to synaptogenesis and synaptic plasticity, including LTP which we discuss below and summarise in the main text (pg. 38, lines 853-868 and pg. 39, lines 876-884).

Integrins containing α5, α8 and αV subunits belong to the RGD family, as they recognise ECM molecules containing the peptide sequence Arg-Gly-Asp (R-G-D) such as fibronectin and thrombospondins. Various integrin heterodimers localise at the synapse, particularly at the post-synaptic density. Integrins are key to the highly dynamic morphological alterations observed at the synapse. In primary hippocampal neurons, RGD peptide treatment was shown to induce dendritic spine elongation (Shi & Ethell, 2006). In hippocampal slices, RGD peptides disrupted LTP. Initial potential was normal, but decay was observed to be faster, suggesting integrins to have a role in stabilising synaptic remodelling (Staubli et al, 1990). Blocking integrin signalling with antibodies and in KO mouse models have largely corroborated these findings (McGeachie et al, 2011). Integrins' effects on LTP are thought to be through regulating actin polymerisation and dynamics of post-synaptic AMPA channel insertion into the membrane (Kramar et al, 2003). Finally, activity-driven matrix metalloprotease mediated proteolysis of ECM components and subsequent regulation of functional and structural synaptic plasticity is again integrin dependent (Nagy et al, 2006). Therefore, we strongly believe the ECM/integrin DEGs could be involved in LTP phenotypes we have observed in hippocampal slices.

Nevertheless, we acknowledge dysregulation of ECM component/integrin expression alone is unlikely a complete explanation of the synaptic remodelling phenotypes we have observed. Therefore, as requested by the reviewer, we re-mined our transcriptome data, but were unsuccessful in identifying other additional DEGs that could shed light on another mechanism of LTP regulation. It is worth noting that the transcriptome data was collected from primary neuronal cultures at DIV3 (pre-synaptogenesis) and it is, therefore, difficult to look for explanations for observations in acute hippocampal slices from adult animals with a much more complex ECM composition than we could model in cell culture system.

7- The method section for "Acute hippocampal slice preparation and electrophysiological recordings " should provide a brief summary, instead of merely citing previously published papers.

We have added additional details in the Materials and methods section (pg. 18-20, lines 379-417).

8-In Supple. Fig 3., the fonts in the x-axis look a bit distorted.

Thank you for noticing this, we have fixed it.

REFEREE #3

We are grateful to the referee for the overall positive review of our work and for the detailed constructive and helpful suggestions. Please note that line numbers refer to the unmarked version of the revised manuscript for simplicity. Below find all suggested points and issues bolded and in chronological order with point-to-point comments in italics.

Major concerns

1. The evidence for endocytotic function for FIBCD1 is provided by FACS experiments of N2a cell uptake of CS-4S-fitc and by immunocytochemistry (IHC) of HEK cell uptake of CS-4S-fitc. From the FACS experiments, it is not clear whether CS-4S-fitc is associated with cell surface or internalized. Control experiments are needed whereby endocytosis is inhibited/blocked and/or extracellular fitc is quenched. From the IHC experiments, it is very unclear whether CS-4S-fitc is endocytosed. The images (Fig 3F) show clumps (aggregates?) outside the cell, and similar clumps are seen within (on?) the cell. It is not clear what is being counted (and the graph in Fig 3F lacks units). Confocal microscopy or deconvolution is needed to show that the puncta are within the cell and have sizes consistent with endocytotic vesicles. Immunostaining for V5 (FIBCD1 WT and mutant constructs shown in Fig 3E and S4B) or FLAG (Fig 3H) and endocytotic vesicle markers in N2a (or HEK) cells should be shown.

We are grateful to the reviewer for the helpful suggestions to strengthen our claim that FIBCD1 is an endocytic receptor of chondroitin sulfates.

We first attempted to repeat our FACS experiments but pre-treating the cells with inhibitors of endocytosis, Dynole and PitStop, which have distinct mechanisms of action. However, these experiments were unsuccessful as the cells were too stressed to acquire high-quality flow cytometry data. Instead, we pre-treated HEK293T-FIBCD1 cells with Dynole and PitStop, incubated them with FITC-CS-4S and immediately fixed them for confocal imaging. We observed that both Dynole and PitStop abrogated the internalisation of CS-4S in these cells (Fig 4G). We also re-imaged previously stained cells and acquired Z-stacks with large slice intervals and, when presented in orthogonal view, noted that the puncta appeared to be internalised, more specifically inside the cell as opposed to sitting on top of the cellular membrane (Fig. EV4G) (pg. 29, lines 629-645).

To estimate the size of the puncta, we re-imaged the HEK293T-FIBCD1 cells incubated with FITC-tagged CS-4S and attempted to acquire Z-stack images with the correct settings for subsequent deconvolution (no saturation, Nyquist sampling in XYZ) as per the reviewer's suggestion. We attempted the acquisition using different systems we have available – wide field microscope with sCMOS camera (Zeiss), Yokogawa spinning disk with SoRa unit on an (Olympus) and LSM880 with Airyscan confocal microscope (Zeiss). Unfortunately, due to rapid photobleaching of the FITC tag, we were unable to acquire any Z-stacks with satisfactory signal-to-noise ratio. Frustratingly, FITC is the only tag commercially available for CS-4S. Instead, we attempted to estimate the diameter of the vesicles using single-plane images. In this experiment we acquired single plane XY images with Airyscan detector on the LSM880 using the super resolution mode. We used this modality not only because of the possible lateral resolution gain, but also due to improved sensitivity of the Airyscan detector. Since confocal microscope delivers images with diffraction limited resolution, we expected that we would be unable to estimate diameter size <180nm nor distinguish individual vesicles that were clustering together. To test this and establish the resolution limits of our confocal system, alongside the CS-4S stained cells, we imaged fluorescent multispecs beads of diameters 100nm and 500nm (Invitrogen Tetraspeck microspheres 0.1um and 0.5um). The 100nm sub resolution beads are usually used to create point spread function (PSF) which describes the quality of an optical system

and can be used to estimate its resolution limits (Cole et al, 2011). We measured the full width at half maximum of the fluorescent signal (FWHM) to estimate the diameter of both puncta and beads in ImageJ. As expected, we could accurately estimate the diameter of the 500nm fluorescent beads, but the 100nm beads were overestimated approximately 2-fold. For the CS-4S puncta, we likewise were unable to resolve diameters <150nm (Unpublished Figure 3). Taken together, we unfortunately were unable to accurately estimate the size of the CS-4S puncta in our cells due to technical limitations of the experiment.

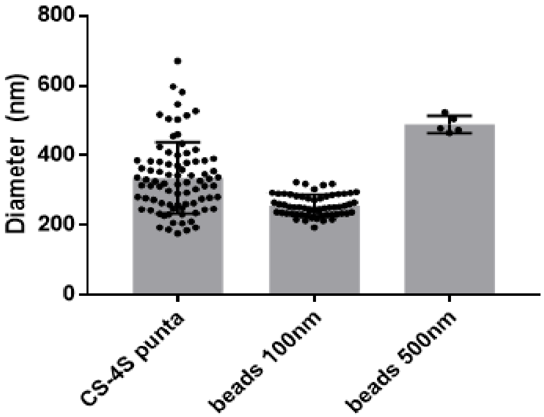

Unpublished Figure 3: Diameter estimation of CS-4S puncta in comparison with fluorescent multispecs beads with indicated diameters. n = 2 for all conditions, each data point represents a field of view.

Finally, as requested by the reviewer, we attempted to co-localise FLAG-FIBCD1 with CS-4S and markers of endocytic vesicles. Due to the rather diffuse staining with an antibody against FLAG (Fig. EV5C), we re-cloned FIBCD1 and overexpressed it as an mCherry-FIBCD1 fusion protein in HEK293T cells. We incubated with FITC-CS-4S and co-stained with LysoTracker Deep Red, which stains acidified vesicles. We noted co-localisation of mCherry-FIBCD1 and CS-4S with LysoTracker, suggesting entry into the lysosomal pathway. Additionally, we also noted co-localisation of mCherry-FIBCD1 with FITC-CS-4S (red arrow) but not with Lysotracker (blue arrow) which could represent pre-lysosomal internalized CS-4S (Fig. 4H) (pg. 29, lines 641-645). Together, these data strongly support the notion that FIBCD1 binds CS-4S and facilitates internalization, likely to lysosomal vesicles.

2. The authors claim to show "evolutionar(il)y conserved" function for FIBCD1 based on behavior phenotypes in human, fly, and mouse. To support this claim, authors should perform phylogenetic analyses and perform similar molecular function experiments across species (e.g. CS levels in fly loss-of-function, molecular dynamics of docking CS-FIBCD from different species, etc). Apart from the phenotypic outcomes, no evidence is provided for conserved molecular function.

We thank the reviewer for the helpful suggestions to strengthen our claim that Fibcd1 has evolutionarily conserved function. We have performed phylogenetic analysis and plotted a phylogenetic tree for the full length FIBCD1 and for the FReD domain only (Fig. EV2B). We estimated Fibcd1's structure using AlphaFold Protein Structure Database (https://alphafold.ebi.ac.uk/search/text/FIBCD1) (April 2022) which allowed us to determine

phylogenetic similarity between different species as well as to perform ligand docking predictions in different species. Indeed, we found that human, mouse and fly FReDs to fold almost identically (Fig. EV2C). Additionally, molecular docking studies of the different species' FReDs indicated similar binding affinities to CS-4S and -6S (Fig. EV2C) (pg.28 lines 609-622) strongly supporting the notion of conserved molecular function among species.

3. The normalization of LTP by ChABC of slices from Fibcd1 KO mice is an intriguing finding, and more so given that ChABC has the inverse effect on WT slices. However, the statement on lines 578-9 that "complete rescue of remodeling deficiencies by ChABC treatment supports our hypothesis that GAG accumulation lies at the centre" of the pathology, is misleading. While paired-pulse facilitation is rescued by ChABC on KO slices, the change in LTP is not in line with the effects of ChABC on WT slices. The rescue of LTP is discussed (lines 665-671) but is not put into the context of the effects observed on WT slices. Clearly the picture is more complex than simply "pathological accumulation", and further experiments are need for the issue to be reconciled. Apart from editorial changes, it may be of interest to treat the slices with various CS compounds. In vivo hippocampal infusion of ChABC and CS compounds to affect WT behavior or rescue KO behavior could also be used to address this issue of "accumulation".

We agree with the reviewer that the picture is likely more complex than simple pathological CS accumulation as the mode of action of ChABC on WTs is not fully understood. Therefore, we have amended the text accordingly (pg.38-39, lines 869-884) to include a more detailed discussion of potential mechanisms and reduce the overinterpretation of the LTP results. Indeed, Reviewer #2 has raised similar questions regarding the difference of ChABC effect on Fibcd1 WT and KO LTP. Please see a lengthy discussion in point #4, Reviewer 2 above.

To clarify this mechanism the reviewer states it may be of interest to perform i) further electrophysiological studies on hippocampal slices and ii) in vivo infusion of ChABC. We strongly agree that treating slices before electrophysiological recordings with various CS compounds may uncover very interesting and completely novel biology, however, we believe that this particular experiment would not address the concerns of the reviewer. As it is not clear what the mechanism of ChABC action on synaptic potentiation is (please see point #4 for Reviewer 2 for background details): whether it is releasing accumulated GAGs, which ones and what size GAG chains, incubating slices with purified and non-endogenous GAGs would not necessarily reflect mechanisms that would occur in vivo and during developmental or learning events. Delineating the functional complexity of differentially sulfated GAGs is of great interest to the field, however, it would require a plethora of additional control experiments that would not be possible in the provided time frame.

The second suggestion of the reviewer to infuse ChABC in vivo and perform behavioral experiments would support the LTP experiments in the manuscript similarly to the published rescue of the Alzheimer disease mouse model with ChABC treatment (Vegh et al., 2014), however, the proposed experiment is not achievable within the given 3-month time frame as it requires a large expansion of the mouse cohort, establishment of stereotaxic injection protocols followed by behavioral phenotyping of the mice, which would likely require closer to 12 months in total as it is not in the core expertise of our research group. More importantly, we feel that in vivo infusions of ChABC would not necessarily help to clarify the relationship between FIBCD1-deficiency and the behavioral deficits observed in the KO mice. ChABC injections would either rescue or fail to rescue the behavioral deficits. Crucially, even were the infusions to rescue the KO behavior, they would serve to only substantiate our findings in Figure 5, which is that ChABC can rescue KO deficiencies, but would not address how ChABC can rescue the deficiencies or clarify the mechanisms underlining the LTP observations. Therefore, the proposed experiment is unlikely to address the concern of the reviewer.

In summary, we feel the proposed experiments are beyond the scope of the current manuscript as it would delve deeply into unexplored biology of chondroitin sulphates and their

relationship to synaptic remodeling, which could detract from the focus of FIBCD1 as a novel disease gene in a rare neurodevelopmental disorder.

Minor concerns

4. Statistics are missing in Figures 1G and 1F.

We thank the reviewer for pointing this out. We have added statistics statement in the Methods section (pg. 8, line 116), and for all legends where it is relevant.

5. The peaks in Figure 3A should be quantified by providing ratios to an internal standard. What is the peak at ~19 minutes?

We thank the reviewer for the question. In line with the reviewer's suggestion, we now provide the relative abundances (i.e. ratios) of the individual chondroitin-sulfate related compounds in figure below (Unpublished Fig. 4) which we obtained by measuring the area under the curve of the presented representative trace (Fig. 4A). Due to the technical limitations of our experimental set up, we were unable to provide quantitative data from the HPLC experiments, only the identity of the peaks and their relative abundance changes.

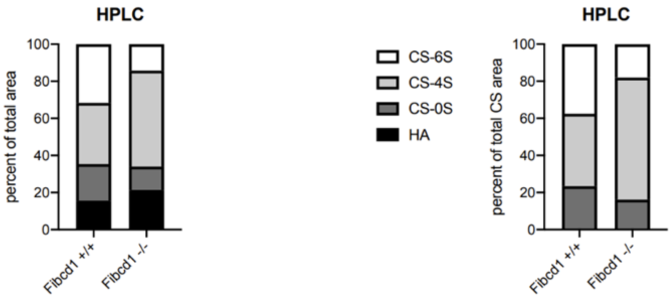

Unpublished Figure 4: Relative abundances (ratios) of the chondroitin sulfate content in the CA1 region of the hippocampus, presented as percent of total area, left, and precent of total CS area, right. Note the relative increase of CS-4S in both graphs, and a slight decrease of CS-6S and CS-0S in *Fibcd1 KOs vs. WT controls*.

Note the relative increase of CS-4S and decrease of CS-6S and CS-0S in both graphs due to FIBCD1 deficiency. To ascertain quantitative data, we had therefore, measured the abundance of the chondroitin sulfates in Fibcd1 WT and KO hippocampi by Western blot analysis from hippocampal lysates of 3 individual mice per cohort. We determined that indeed CS-4S abundance is significantly increased in Fibcd1 KO animals, while CS-6S and CS-0S did not change (Fig. 4B).

As the analysis of ChABC released and 2-AB labelled disaccharides was performed by HPLC analysis coupled to a fluorescence detection system (rather than e.g. a mass-spectrometer), we can unfortunately not comment on the identity of the peak at ~19 min. CS related compounds indicated in Fig 4A (i.e. CS-0S, CS-4S and CS-6S, as well as HA) were identified based on their elution time-points with regard to the respective reference compounds.

6. In Figures 4A and 4B, there are discrepancies in the N given in the legend and the number of points in the graph. The graph in Fig 4B lacks units.

We thank the reviewer for pointing out the discrepancy. We have corrected the error in the legend.

7. Statistics are missing in Figure S1B-D.

We thank the reviewer for pointing this out. We have added statistics in the Methods section (pg. 8, line 116), as well as in all the legends where it is relevant. Please note that Fig. EV1C is from a public repository (brainrnaseq.org) where N numbers are unknown, and therefore should be treated as qualitative rather than quantitative data.

We hope we have sufficiently addressed all the concerns and suggestions raised by the Editor and the 3 Reviewers. We are grateful for your consideration and are available shall any additional information be required.

Best Regards,

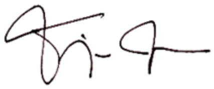

Vanja Nagy, PhD

Principal Investigator
LBI-RUD; Ludwig Boltzmann Institute for Rare and Undiagnosed Diseases, Vienna, Austria
Email: Vanja.nagy@rud.lbg.ac.at
Website: www.vanjanagylab.com

References:

Bukalo O, Schachner M, Dityatev A (2001) Modification of extracellular matrix by enzymatic removal of chondroitin sulfate and by lack of tenascin-R differentially affects several forms of synaptic plasticity in the hippocampus. *Neuroscience* 104: 359-369
Cole RW, Jinadasa T, Brown CM (2011) Measuring and interpreting point spread functions to determine confocal microscope resolution and ensure quality control. *Nat Protoc* 6: 1929-1941
Graca FA, Rai M, Hunt LC, Stephan A, Wang YD, Gordon B, Wang R, Quarato G, Xu B, Fan Y *et al* (2022) The myokine Fibcd1 is an endogenous determinant of myofiber size and mitigates cancer-induced myofiber atrophy. *Nat Commun* 13: 2370
Kramar EA, Bernard JA, Gall CM, Lynch G (2003) Integrins modulate fast excitatory transmission at hippocampal synapses. *J Biol Chem* 278: 10722-10730

McGeachie AB, Cingolani LA, Goda Y (2011) Stabilising influence: integrins in regulation of synaptic plasticity. *Neurosci Res* 70: 24-29
Mencio CP, Hussein RK, Yu P, Geller HM (2021) The Role of Chondroitin Sulfate Proteoglycans in Nervous System Development. *J Histochem Cytochem* 69: 61-80
Nagy V, Bozdagi O, Matynia A, Balcerzyk M, Okulski P, Dzwonek J, Costa RM, Silva AJ, Kaczmarek L, Huntley GW (2006) Matrix metalloproteinase-9 is required for hippocampal late-phase long-term potentiation and memory. *J Neurosci* 26: 1923-1934
Rowlands D, Sugahara K, Kwok JC (2015) Glycosaminoglycans and glycomimetics in the central nervous system. *Molecules* 20: 3527-3548
Saroja SR, Sase A, Kircher SG, Wan J, Berger J, Hoger H, Pollak A, Lubec G (2014) Hippocampal proteoglycans brevican and versican are linked to spatial memory of Sprague-Dawley rats in the morris water maze. *J Neurochem* 130: 797-804
Schlosser A, Thomsen T, Moeller JB, Nielsen O, Tornoe I, Mollenhauer J, Moestrup SK, Holmskov U (2009) Characterization of FIBCD1 as an acetyl group-binding receptor that binds chitin. *J Immunol* 183: 3800-3809
Senkov O, Andjus P, Radenovic L, Soriano E, Dityatev A (2014) Neural ECM molecules in synaptic plasticity, learning, and memory. *Prog Brain Res* 214: 53-80
Shi Y, Ethell IM (2006) Integrins control dendritic spine plasticity in hippocampal neurons through NMDA receptor and Ca2+/calmodulin-dependent protein kinase II-mediated actin reorganization. *J Neurosci* 26: 1813-1822
Staubli U, Vanderklish P, Lynch G (1990) An inhibitor of integrin receptors blocks long-term potentiation. *Behav Neural Biol* 53: 1-5
Vegh MJ, Heldring CM, Kamphuis W, Hijazi S, Timmerman AJ, Li KW, van Nierop P, Mansvelder HD, Hol EM, Smit AB *et al* (2014) Reducing hippocampal extracellular matrix reverses early memory deficits in a mouse model of Alzheimer's disease. *Acta Neuropathol Commun* 2: 76
Yamada S, Sugahara K, Ozbek S (2011) Evolution of glycosaminoglycans: Comparative biochemical study. *Commun Integr Biol* 4: 150-158

1st Revision - Editorial Decision 27th Jun 2022

27th Jun 2022

Dear Dr. Nagy,

Thank you for the submission of your revised manuscript to EMBO Molecular Medicine. We have now received the enclosed report from the three referees who were asked to re-assess it. As you will see, the referees are now supportive, and I am pleased to inform you that we will be able to accept your manuscript pending the following amendments:

1. Please address the remaining minor concerns of Referee #3.

On a more editorial level:

1. Please reorder the individual manuscript sections in the correct order (please refer to any published papers)
- Also, "Figure titles and Legends" should be corrected to "Figure Legends".
- "SUPPLEMENTAL FIGURES TITLES AND LEGENDS" should be corrected to "Expanded View Figure Legends".

2. Author contribution: Venkat Swaroop Achuta, Margit Burmeister, Biswa Choudhury are missing. Please fix it.

3. We updated our journal's competing interests policy in January 2022 and request authors to consider both actual and perceived competing interests. Please review the policy https://www.embopress.org/competing-interests and update your competing interests if necessary.
Please use the heading "Disclosure statement and competing interests".

4. Please list up to 10 co-authors of a paper before adding et al. to the reference list.

5. Appendix: Please add a table of content to the 1st page. The legend for the Appendix figure should be added to the Appendix and removed from the main manuscript file.

6. I have slightly shortened and modified the synopsis text (see attached). Please let me know if it is fine as is or if you would like to introduce further modifications.

7. Our data editors have seen the manuscript, and they have made some comments and suggestions that need answering (in track change mode, see attached). Please send back a track changes file as we will need to go through the changes.

8. As part of the EMBO Publications transparent editorial process initiative (see our Editorial at http://embomolmed.embopress.org/content/2/9/329), EMBO Molecular Medicine will publish online a Review Process File (RPF) to accompany accepted manuscripts.

In the event of acceptance, this file will be published in conjunction with your paper and will include the anonymous referee reports, your point-by-point response, and all pertinent correspondence relating to the manuscript. Let us know if you DISAGREE with this and if you want to remove or keep any figures from it prior to publication.

Please note that the Authors checklist will be published at the end of the RPF.

Please submit your revised manuscript within two weeks. I look forward to seeing a revised form of your manuscript as soon as possible.

Kind regards,
Jingyi

Jingyi Hou
Editor
EMBO Molecular Medicine

***** Reviewer's comments *****

Referee #1 (Comments on Novelty/Model System for Author):

I found this to be a thorough and thoughtful revision. Much was done to address the concerns and the manuscript is very much improved. I find the study to novel and exciting--and the breadth contained within is unusual.

Referee #1 (Remarks for Author):

This revised manuscript has fully addressed the concerns and questions that were raised in the previous version. It reads well, and I find it to be an original, very thorough, and important contribution to the field.

Referee #2 (Remarks for Author):

The authors addressed my concerns.

Referee #3 (Remarks for Author):

The manuscript by Fell et al. investigates the consequences of point mutations in FIBCD1 identified in two patients with idiopathic NDDs. Analysis of human and mouse tissue shows high FIBCD1 expression in the glands, testis, and CNS, more specifically in hypothalamus and hippocampus. Expression knockdown in fly nervous system results in reduced locomotion, while a mouse knock-out (KO) model result in impaired fear-associated learning. Hippocampal slices from KO mice have impaired synaptic plasticity that is rescued by digestion of CSPGs. The authors convincingly show that FIBCD1 preferentially binds to chondroitin sulfate (CS) with 4-O sulfation (CS-4S), and that this binding is disrupted by mutations corresponding to the variants identified in the two patients. They also show that FIBCD1 mediates CS-4S endocytosis into lysosomal compartments. Analysis of primary hippocampal cultures suggest that a consequence of FIBCD1 activity is the regulation of genes related to integrins and the extracellular matrix. The implications of their findings are thoroughly discussed and provide new insight into NDDs.

Minor comments

1. Line 586 refers to comparison of red and pink traces, but Fig 3F shows statistical significance between light blue and pink; it would seem this stat is misplaced.

2. Fig 4H is difficult to visualize. It would be helpful if the 4 smaller panels were zoomed-in rather than zoomed-out.

3. Fig EV5C is very difficult to visualize; consider improving contrast.

The authors performed the requested editorial changes.

7th Jul 2022

Dear Dr. Nagy,

We are pleased to inform you that your manuscript is accepted for publication and is now being sent to our publisher to be included in the next available issue of EMBO Molecular Medicine.

We would like to remind you that as part of the EMBO Publications transparent editorial process initiative, EMBO Molecular Medicine will publish a Review Process File online to accompany accepted manuscripts. If you do NOT want the file to be published or would like to exclude figures, please immediately inform the editorial office via e-mail.

Please read below for additional IMPORTANT information regarding your article, its publication and the production process.

Congratulations on your interesting work,

Jingyi

Jingyi Hou
Editor
EMBO Molecular Medicine

Follow us on Twitter @EmboMolMed
Sign up for eTOCs at embopress.org/alertsfeeds

EMBO Press Author Checklist

Corresponding Author Name: Josef M Penninger and Vanja Nagy	
Journal Submitted to: EMBO Molecular Medicine	
Manuscript Number: EMM-2022-15829	

USEFUL LINKS FOR COMPLETING THIS FORM
The EMBO Journal - Author Guidelines
EMBO Reports - Author Guidelines
Molecular Systems Biology - Author Guidelines
EMBO Molecular Medicine - Author Guidelines

Reporting Checklist for Life Science Articles (updated January 2022)
This checklist is adapted from Materials Design Analysis Reporting (MDAR) Checklist for Authors. MDAR establishes a minimum set of requirements in transparent reporting in the life sciences (see Statement of Task: 10.31222/osf.io/9sm4x). Please follow the journal's guidelines in preparing your manuscript.
Please note that a copy of this checklist will be published alongside your article.

Abridged guidelines for figures
1. Data
The data shown in figures should satisfy the following conditions:
- the data were obtained and processed according to the field's best practice and are presented to reflect the results of the experiments in an accurate and unbiased manner.
- ideally, figure panels should include only measurements that are directly comparable to each other and obtained with the same assay.
- plots include clearly labeled error bars for independent experiments and sample sizes. Unless justified, error bars should not be shown for technical replicates.
- if n<5, the individual data points from each experiment should be plotted. Any statistical test employed should be justified.
- Source Data should be included to report the data underlying figures according to the guidelines set out in the authorship guidelines on Data Presentation.

2. Captions
Each figure caption should contain the following information, for each panel where they are relevant:
- a specification of the experimental system investigated (eg cell line, species name).
- the assay(s) and method(s) used to carry out the reported observations and measurements.
- an explicit mention of the biological and chemical entity(ies) that are being measured.
- an explicit mention of the biological and chemical entity(ies) that are altered/varied/perturbed in a controlled manner.
- the exact sample size (n) for each experimental group/condition, given as a number, not a range;
- a description of the sample collection allowing the reader to understand whether the samples represent technical or biological replicates (including how many animals, litters, cultures, etc.).
- a statement of how many times the experiment shown was independently replicated in the laboratory.
- definitions of statistical methods and measures:
  - common tests, such as t-test (please specify whether paired vs. unpaired), simple χ2 tests, Wilcoxon and Mann-Whitney tests, can be unambiguously identified by name only, but more complex techniques should be described in the methods section;
  - are tests one-sided or two-sided?
  - are there adjustments for multiple comparisons?
  - exact statistical test results, e.g., P values = x but not P values < x;
  - definition of 'center values' as median or average;
  - definition of error bars as s.d. or s.e.m.

Please complete ALL of the questions below.
Select "Not Applicable" only when the requested information is not relevant for your study.

Materials

Newly Created Materials	Information included in the manuscript?	In which section is the information available? (Reagents and Tools Table, Materials and Methods, Figures, Data Availability Section)
New materials and reagents need to be available; do any restrictions apply?	Not Applicable	

Antibodies	Information included in the manuscript?	In which section is the information available? (Reagents and Tools Table, Materials and Methods, Figures, Data Availability Section)
For **antibodies** provide the following information:<br>- Commercial antibodies: RRID (if possible) or supplier name, catalogue number and or/clone number<br>- Non-commercial: RRID or citation	Yes	Table 2

DNA and RNA sequences	Information included in the manuscript?	In which section is the information available? (Reagents and Tools Table, Materials and Methods, Figures, Data Availability Section)
**Short novel DNA or RNA including primers, probes**: provide the sequences.	Yes	Table 2

Cell materials	Information included in the manuscript?	In which section is the information available? (Reagents and Tools Table, Materials and Methods, Figures, Data Availability Section)
**Cell lines:** Provide species information, strain. Provide accession number in repository **OR** supplier name, catalog number, clone number, and/**OR** RRID.	Yes	Materials and methods
**Primary cultures:** Provide species, strain, sex of origin, genetic modification status.	Yes	Materials and methods
Report if the cell lines were recently **authenticated** (e.g., by STR profiling) and tested for mycoplasma contamination.	Yes	Materials and methods

Experimental animals	Information included in the manuscript?	In which section is the information available? (Reagents and Tools Table, Materials and Methods, Figures, Data Availability Section)
**Laboratory animals or Model organisms:** Provide species, strain, sex, age, genetic modification status. Provide accession number in repository **OR** supplier name, catalog number, clone number, **OR** RRID.	Yes	Materials and methods
**Animal observed in or captured from the field:** Provide species, sex, and age where possible.	Not Applicable	
Please detail **housing and husbandry conditions**.	Yes	Materials and methods

Plants and microbes	Information included in the manuscript?	In which section is the information available? (Reagents and Tools Table, Materials and Methods, Figures, Data Availability Section)
**Plants:** provide species and strain, ecotype and cultivar where relevant, unique accession number if available, and source (including location for collected wild specimens).	Not Applicable	
**Microbes:** provide species and strain, unique accession number if available, and source.	Not Applicable	

Human research participants	Information included in the manuscript?	In which section is the information available? (Reagents and Tools Table, Materials and Methods, Figures, Data Availability Section)
If collected and within the bounds of privacy constraints report on age, sex and gender or ethnicity for all study participants.	Not Applicable	

Core facilities	Information included in the manuscript?	In which section is the information available? (Reagents and Tools Table, Materials and Methods, Figures, Data Availability Section)
If your work benefited from core facilities, was their service mentioned in the acknowledgments section?	Yes	Acknowledgments

Design

Study protocol	Information included in the manuscript?	In which section is the information available? (Reagents and Tools Table, Materials and Methods, Figures, Data Availability Section)
If study protocol has been **pre-registered, provide DOI in the manuscript**. For clinical trials, provide the trial registration number **OR** cite DOI.	Not Applicable	
Report the **clinical trial registration number** (at ClinicalTrials.gov or equivalent), where applicable.	Not Applicable	

Laboratory protocol	Information included in the manuscript?	In which section is the information available? (Reagents and Tools Table, Materials and Methods, Figures, Data Availability Section)
Provide DOI OR other citation details if **external detailed step-by-step protocols** are available.	Not Applicable	

Experimental study design and statistics	Information included in the manuscript?	In which section is the information available? (Reagents and Tools Table, Materials and Methods, Figures, Data Availability Section)
Include a statement about **sample size** estimate even if no statistical methods were used.	Not Applicable	
Were any steps taken to minimize the effects of subjective bias when allocating animals/samples to treatment (e.g. **randomization procedure**)? If yes, have they been described?	Not Applicable	
Include a statement about **blinding** even if no blinding was done.	Yes	Materials and methods
Describe **inclusion/exclusion criteria** if samples or animals were excluded from the analysis. Were the criteria pre-established?<br><br>If sample or data points were omitted from analysis, report if this was due to attrition or intentional exclusion and provide justification.	Not Applicable	
For every figure, are **statistical tests** justified as appropriate? Do the data meet the assumptions of the tests (e.g., normal distribution)? Describe any methods used to assess it. Is there an estimate of variation within each group of data? Is the variance similar between the groups that are being statistically compared?	Yes	Figure legends

Sample definition and in-laboratory replication	Information included in the manuscript?	In which section is the information available? (Reagents and Tools Table, Materials and Methods, Figures, Data Availability Section)
In the figure legends: state number of times the experiment was **replicated** in laboratory.	Yes	Figure legends
In the figure legends: define whether data describe **technical or biological replicates**.	Yes	Figure legends

Ethics

Ethics	Information included in the manuscript?	In which section is the information available? (Reagents and Tools Table, Materials and Methods, Figures, Data Availability Section)
Studies involving **human participants**: State details of **authority granting ethics approval** (IRB or equivalent committee(s), provide reference number for approval.	Not Applicable	
Studies involving **human participants**: Include a statement confirming that **informed consent** was obtained from all subjects and that the experiments conformed to the principles set out in the WMA Declaration of Helsinki and the Department of Health and Human Services Belmont Report.	Not Applicable	
Studies involving **human participants:** For publication of **patient photos**, include a statement confirming that consent to publish was obtained.	Not Applicable	
Studies involving experimental **animals**: State details of **authority granting ethics approva**l (IRB or equivalent committee(s), provide reference number for approval. Include a statement of compliance with ethical regulations.	Yes	Materials and methods
Studies involving **specimen and field samples:** State if relevant **permits** obtained, provide details of authority approving study; if none were required, explain why.	Not Applicable	

Dual Use Research of Concern (DURC)	Information included in the manuscript?	In which section is the information available? (Reagents and Tools Table, Materials and Methods, Figures, Data Availability Section)
Could your study fall under dual use research restrictions? Please check biosecurity documents and list of **select agents and toxins** (CDC): https://www.selectagents.gov/sat/list.htm	Not Applicable	
If you used a select agent, is the security level of the lab appropriate and reported in the manuscript?	Not Applicable	
If a study is subject to dual use research of concern regulations, is the name of the **authority granting approval and reference number** for the regulatory approval provided in the manuscript?	Not Applicable	

Reporting

The MDAR framework recommends adoption of discipline-specific guidelines, established and endorsed through community initiatives. Journals have their own policy about requiring specific guidelines and recommendations to complement MDAR.

Adherence to community standards	Information included in the manuscript?	In which section is the information available? (Reagents and Tools Table, Materials and Methods, Figures, Data Availability Section)
State if relevant guidelines or checklists (e.g., **ICMJE, MIBBI, ARRIVE, PRISMA**) have been followed or provided.	Yes	ARRIVE - materials and methods
For **tumor marker prognostic studies,** we recommend that you follow the **REMARK** reporting guidelines (see link list at top right). See author guidelines, under 'Reporting Guidelines'. Please confirm you have followed these guidelines.	Not Applicable	
For **phase II and III randomized controlled trials**, please refer to the **CONSORT** flow diagram (see link list at top right) and submit the CONSORT checklist (see link list at top right) with your submission. See author guidelines, under 'Reporting Guidelines'. Please confirm you have submitted this list.	Not Applicable	

Data Availability

Data availability	Information included in the manuscript?	In which section is the information available? (Reagents and Tools Table, Materials and Methods, Figures, Data Availability Section)
Have **primary datasets** been deposited according to the journal's guidelines (see 'Data Deposition' section) and the respective accession numbers provided in the Data Availability Section?	Yes	GEO omnibus, link in materials and methods
Were **human clinical and genomic datasets** deposited in a public access-controlled repository in accordance to ethical obligations to the patients and to the applicable consent agreement?	Not Applicable	
Are **computational models** that are central and integral to a study available without restrictions in a machine-readable form? Were the relevant accession numbers or links provided?	Not Applicable	
If publicly available data were reused, provide the respective **data citations in the reference list**.	Yes	References
